# Photoactive Thiophene‐Enriched Tetrathienonaphthalene‐Based Covalent Organic Frameworks

**DOI:** 10.1002/smll.202511000

**Published:** 2025-11-07

**Authors:** Tianhao Xue, Marcello Righetto, Roman Guntermann, Shizhe Wang, Dominic Blätte, Zehua Xu, Andreas Weis, Ignacio Munoz‐Alonso, Dana D. Medina, Achim Hartschuh, Laura M. Herz, Thomas Bein

**Affiliations:** ^1^ Department of Chemistry and Center for NanoScience (CeNS) Ludwig‐Maximilians‐Universität (LMU) Butenandtstraße 5–13 81377 Munich Germany; ^2^ Department of Physics Clarendon Laboratory University of Oxford Oxford OX1 3PU UK

**Keywords:** covalent organic frameworks, optical‐pump terahertz‐probe spectroscopy, photoactive, tetrathienonaphthalene

## Abstract

The optoelectronic properties of covalent organic frameworks (COFs) can be controlled by the design of their molecular building blocks and assembly. Here, a facile and efficient synthetic route is reported for the novel thiophene‐enriched tetrathienonaphthalene (TTN)‐based node 4,4′,4″,4′″‐(naphtho[1,2‐*b*:4,3‐*b*′:5,6‐*b*″:8,7‐*b*″′]tetrathiophene‐2,5,8,11‐tetrayl)tetraaniline (TTNTA) for constructing imine‐linked COFs. Utilizing TTNTA, highly crystalline, thiophene‐enriched donor–donor (D–D) and donor–acceptor (D–A) COFs, denoted as TT COF and BDT(BT)_2_ COF, are synthesized using two distinct aldehyde‐functionalized linear linkers: [2,2′‐bithiophene]‐5,5′‐dicarbaldehyde (TT) and 7,7′‐(4,8‐diethoxybenzo[1,2‐*b*:4,5‐*b*′]dithiophene‐2,6‐diyl)bis(benzo[*c*][1,2,5]thiadiazole‐4‐carbaldehyde) (BDT(BT)_2_), respectively. Highly crystalline and oriented TTNTA COF films on various substrates via a solvothermal method enabled further comprehensive optical and electronic characterizations. Optical‐pump terahertz‐probe spectroscopy revealed effective charge‐carrier mobility values *φμ* = 0.34 ± 0.04  and 0.18 ± 0.02 cm^2^V^−1^s^−1^ for TT and BDT(BT)_2_ COF films, respectively. These results reveal distinct charge‐transport characteristics and provide mechanistic insights into their ultrafast charge‐carrier dynamics. The COFs are demonstrated to be photoactive, showing promising potential as photocathodes without co‐catalysts in photoelectrochemical water splitting, with notable photocurrent densities of 10 and 15.3 µA cm^−2^ after 1 h illumination, respectively. This work highlights the potential of TTNTA‐based COFs in optoelectronic applications and provides insights into the design of thiophene‐enriched COFs with high crystallinity and photoactive behavior.

## Graphical abstract

Highly crystalline tetrathienonaphthalene‐based covalent organic frameworks are synthesized in both bulk and thin‐film forms using a novel thiophene‐rich tetraaniline‐functionalized node (TTNTA). These COFs exhibit remarkable charge carrier mobilities in the field of 2D COFs and intrinsic photoactivity. The introduction of the TTNTA node provides a versatile platform for designing thiophene‐enriched COFs for light‐driven applications.

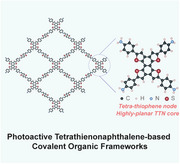

## 1 Introduction

Covalent organic frameworks (COFs) featuring precisely defined structures and topologies have captured significant attention since first reported by Yaghi et al. in 2005.^[^ ^1^ ^]^ The integration of diverse building blocks via different molecular linkages enables the synthesis of COFs with a wide range of topologies and tunable optical and electronic properties and functionalities.^[^ ^2^, ^3^, ^4^, ^5^ ^]^ Consequently, a critical imperative emerges for the development of novel building blocks aimed at achieving specific properties, underscoring the importance of molecular design in advancing COF research. Thiophene and its derivatives, well known for their electron‐rich nature and favorable light‐harvesting capabilities, have been successfully incorporated into conjugated polymers, demonstrating their significant promise in a wide range of applications such as organic solar cells (OSCs),^[^ ^6^, ^7^ ^]^ organic field‐effect transistors (OFETs),^[^ ^8^, ^9^ ^]^ and organic light‐emitting diodes (OLEDs).^[^ ^10^, ^11^ ^]^ Notably, numerous studies have revealed that the precise arrangement and orientation of thiophene‐based conjugated polymers significantly influence their optical and electronic properties.^[^ ^12^, ^13^ ^]^ 2D COFs provide an ideal platform for achieving highly ordered spatial thiophene arrangements and control of electronic interactions due to their crystalline structure and the stacking behavior of COF layers. Various thiophene‐containing 2D COFs have been reported to date, showcasing their considerable potential in photocatalysis,^[^ ^14^, ^15^ ^]^ photovoltaics,^[^ ^16^, ^17^ ^]^ optoelectronics,^[^ ^18^, ^19^ ^]^ and energy storage.^[^ ^20^, ^21^ ^]^ For instance, *C*_3_‐symmetric benzotrithiophene (BTT)‐based 2D COFs have demonstrated considerable promise in light‐driven applications ever since BTT was first incorporated as a node into 2D COFs.^[^ ^22^, ^23^, ^24^ ^]^ Very recently, thiophene‐extended BTT‐based 2D COFs have emerged as notable candidates due to their strongly directional, defect‐dominated charge transport characteristics when fabricated as oriented films.^[^ ^25^ ^]^ Besides BTT‐based 2D COFs, tetrathiafulvalene (TTF)‐based 2D COFs known for their electroactivity due to two reversible oxidation processes of the TTF moiety,^[^ ^26^ ^]^ have shown the potential in energy storage.^[^ ^20^, ^21^ ^]^ Although a variety of thiophene‐based building blocks have been successfully incorporated into COFs,^[^ ^27^, ^28^, ^29^ ^]^ the overall availability of diverse thiophene‐based building blocks, particularly as nodes, remains highly restricted. The integration of thiophene derivatives continues to present significant challenges, primarily attributed to the unique angular geometries of five‐membered rings and the intricacies of synthetic methodologies. Consequently, the design and development of novel thiophene‐containing building blocks are essential for the efficient synthesis of thiophene‐enriched COFs, enabling potential light‐driven applications.

Tetrathienonaphthalene (TTN) featuring four thiophenes fused onto a naphthalene unit shows excellent chemical and thermal stability, is known as p‐type organic semiconductor and has been studied for potential applications in OFETs,^[^ ^30^ ^]^ and OSCs.^[^ ^31^ ^]^ Notably, the highly planar structure of TTN benefits the enhancement of intermolecular *π–π* stacking and charge carrier transport, yielding a high hole mobility.^[^ ^32^ ^]^ However, despite its outstanding properties, practical implementations have been limited, due to its highly planar structure and resulting low solubility. Long alkyl chains are generally required to increase solubility, leading to complex synthetic methods often involving photoirradiation.^[^ ^33^, ^34^ ^]^

Herein, we present a facile synthetic approach for a novel TTN‐based node, namely 4,4′,4″,4′″‐(naphtho[1,2‐*b*:4,3‐*b*′:5,6‐*b*″:8,7‐*b*′″]tetrathiophene‐2,5,8,11‐tetrayl)tetraaniline (TTNTA). For the first time, we successfully incorporated a TTN moiety into 2D COFs, enabling the formation of highly crystalline thiophene‐enriched donor–donor (D–D, TT COF) and donor–acceptor (D–A, BDT(BT)_2_ COF) imine‐linked COFs. This was achieved via acid‐catalyzed Schiff base condensation reactions with two linear monomers, [2,2′‐bithiophene]‐5,5′‐dicarbaldehyde (TT) and 7,7′‐(4,8‐diethoxybenzo[1,2‐*b*:4,5‐*b*′]dithiophene‐2,6‐diyl)bis(benzo[*c*][1,2,5]thiadiazole‐4‐carbaldehyde) (BDT(BT)_2_), respectively. Moreover, the synthesis of crystalline and highly oriented TTNTA COF films on various substrates was achieved by means of a solvothermal method.^[^ ^25^, ^29^, ^35^ ^]^ The terahertz photoconductivity and charge‐carrier dynamics of both COF films were studied by using optical‐pump terahertz‐probe spectroscopy. Furthermore, TTNTA COF films were evaluated as potential photocathodes for photoelectrochemical hydrogen production from water.

## 2 Results and Discussion

**Scheme 1:**
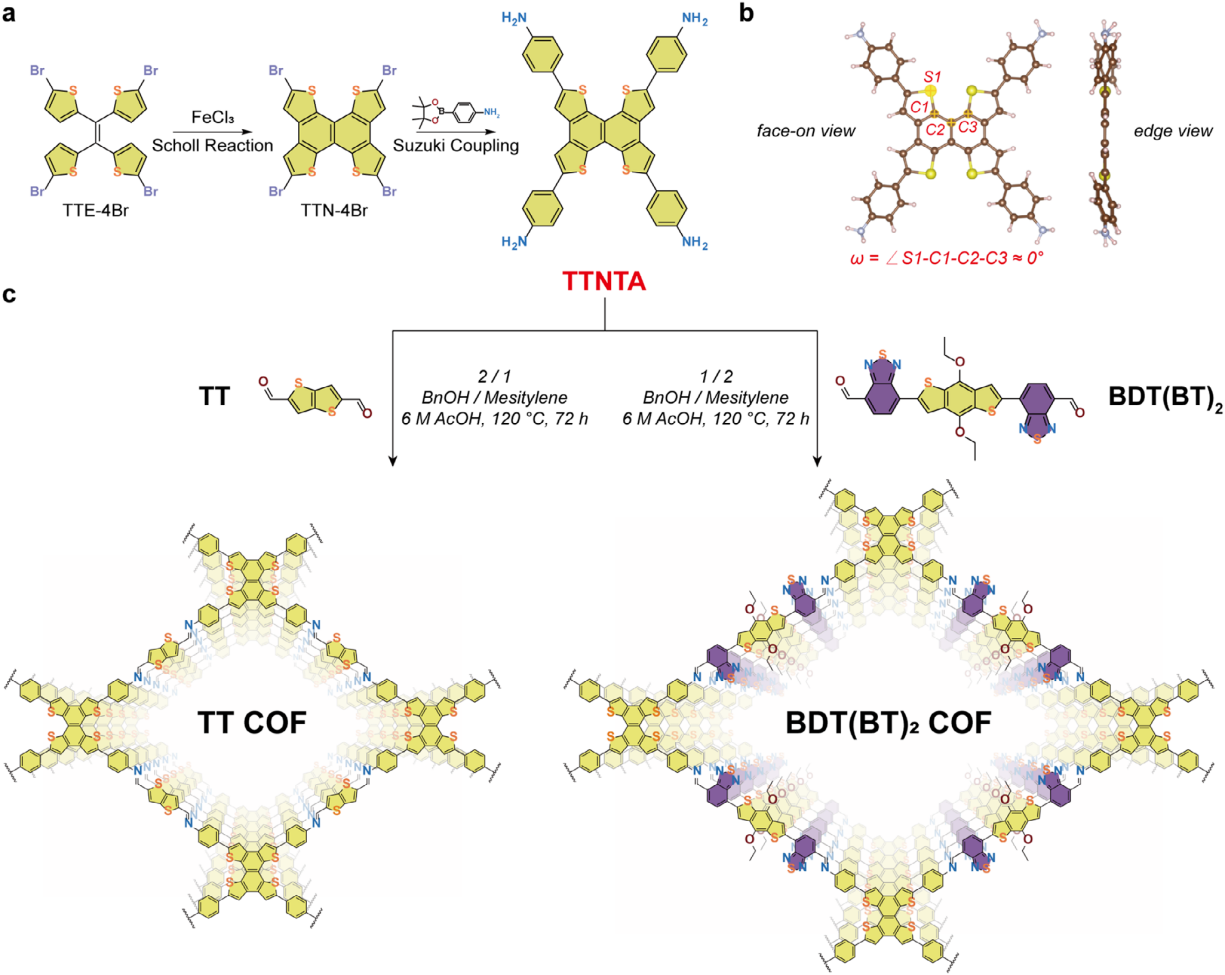
a) Key steps for the synthesis of TTNTA via a Scholl reaction to induce ring closure, followed by a Suzuki coupling to functionalize the TTN core with an aniline moiety, to enable the subsequent COF synthesis. b) DFT calculated crystal structure of TTNTA, showing face‐on (left) and edge (right) views. c) Synthetic procedures for the formation of TT and BDT(BT)_2_ COFs and structural view of the resulting 2D COFs with rhombic pore architectures.

To ultimately incorporate the TTN moiety with its highly planar structural features into the synthesis of COFs, we pursued a facile approach toward creating a reactive node (**Scheme** **1**a; Scheme S1, Supporting Information). First, 1,1,2,2‐tetrakis(5‐bromothiophen‐2‐yl)ethene (TTE‐4Br) was synthesized through classical McMurry coupling with subsequent Wohl‐Ziegler bromination, following previously established procedures.^[^ ^30^, ^36^ ^]^ Departing from the well‐established and complex photoinduced 6π‐electrocyclization‐dehydrogenation reaction, Scholl reaction conditions, commonly used in the synthesis of nanographenes, were employed for the ring‐closure reaction for the first time to yield 2,5,8,11‐tetrabromonaphtho[1,2‐*b*:4,3‐*b*′:5,6‐*b*″:8,7‐*b*′″]tetrathiophene (TTN‐4Br). Herein, the elimination of four aryl‐bound hydrogens from thiophene rings accompanied by the formation of two aryl–aryl bonds, was achieved under the influence of FeCl_3_ as a Friedel‐Crafts catalyst. Subsequent Suzuki coupling reactions facilitated the formation of the TTN‐based node with four terminal amino groups pre‐designed for the further construction of imine‐COFs, abbreviated as TTNTA. The successful synthesis of the TTNTA node in high purity was confirmed by ^1^H NMR and ^13^C NMR spectroscopy (Figure S1e,f, Supporting Information). Density functional theory (DFT) calculations revealed the remarkable planarity of the TTN core (edge‐view in Scheme 1b), attributed to the absence of steric hindrance from four hydrogen atoms, in contrast to a structurally similar dibenzochrysene(DBC)‐based node (Figure S2, Supporting Information).^[^ ^37^ ^]^ The DBC‐based core exhibits a pronounced torsional distortion (ω ≈ 9.5°), whereas TTN adopts a nearly coplanar geometry (ω ≈ 0°), which facilitates extended π‐conjugation. This increased planarity is further reflected in the slightly reduced bandgap (3.25 eV for TTN‐based node vs 3.38 eV for DBC‐based node), supporting its favorable optoelectronic properties. Thus, only the attached phenyl rings can rotate out of plane, but this has been shown to have negligible impact on the interlayer stacking of the resulting COFs, as supported by our previous findings.^[^ ^37^ ^]^

The optical properties of the TTNTA node were characterized by ultraviolet‐visible (UV–vis) and photoluminescence (PL) spectroscopy on 50 µm TTNTA solutions in DMSO (Figure S3, Supporting Information). The solution exhibits a strong UV–vis absorption peak at ≈406 nm, yielding a vivid golden‐yellow color in solution as observed by the naked eye. A green fluorescence emission can be observed under a standard 365 nm UV lamp, in accordance with the observed PL peak at 512 nm. Cyclic voltammetry (CV) of TTNTA (Figure S4, Supporting Information) exhibited a pronounced oxidative response with an onset potential of ≈+0.38 V versus the ferrocene/ferrocenium (Fc/Fc^+^) redox couple. The relatively low oxidation onset potential facilitates oxidation at moderate electrochemical conditions, consistent with its electron‐rich nature.

To construct imine‐linked COFs, two linear aldehyde‐functionalized building blocks, TT and BDT(BT)_2_, were employed to connect the TTNTA nodes (Scheme 1c). Notably, this work introduces a linear linker with an acceptor–donor–acceptor (A–D–A) configuration, which features an electron‐rich benzo[1,2‐*b*:4,5‐*b*']dithiophene (BDT) core with ethoxy substituents, coupled with two electron‐deficient benzo[*c*][1,2,5]thiadiazole (BT) units. The synthesis of this novel linear building block was achieved via Stille coupling of a 2,8‐stannylated BDT unit with two 4‐bromo‐7‐(5,5‐dimethyl‐1,3‐dioxan‐2‐yl)benzo[*c*][1,2,5]thiadiazole units, followed by acetal deprotection under acidic conditions to yield the desired BDT(BT)_2_ building block, similar to our previous work (Scheme S2, Supporting Information).^[^ ^38^ ^]^ The successful synthesis of the BDT(BT)_2_ building block was confirmed by ^1^H NMR spectroscopy of acetal protected BDT(BT)_2_ as intermediate (Figure S1m, Supporting Information) as well as HRMS‐EI spectra of the final product, due to its extremely low solubility (details in the Supporting Information).

**Figure 1:**
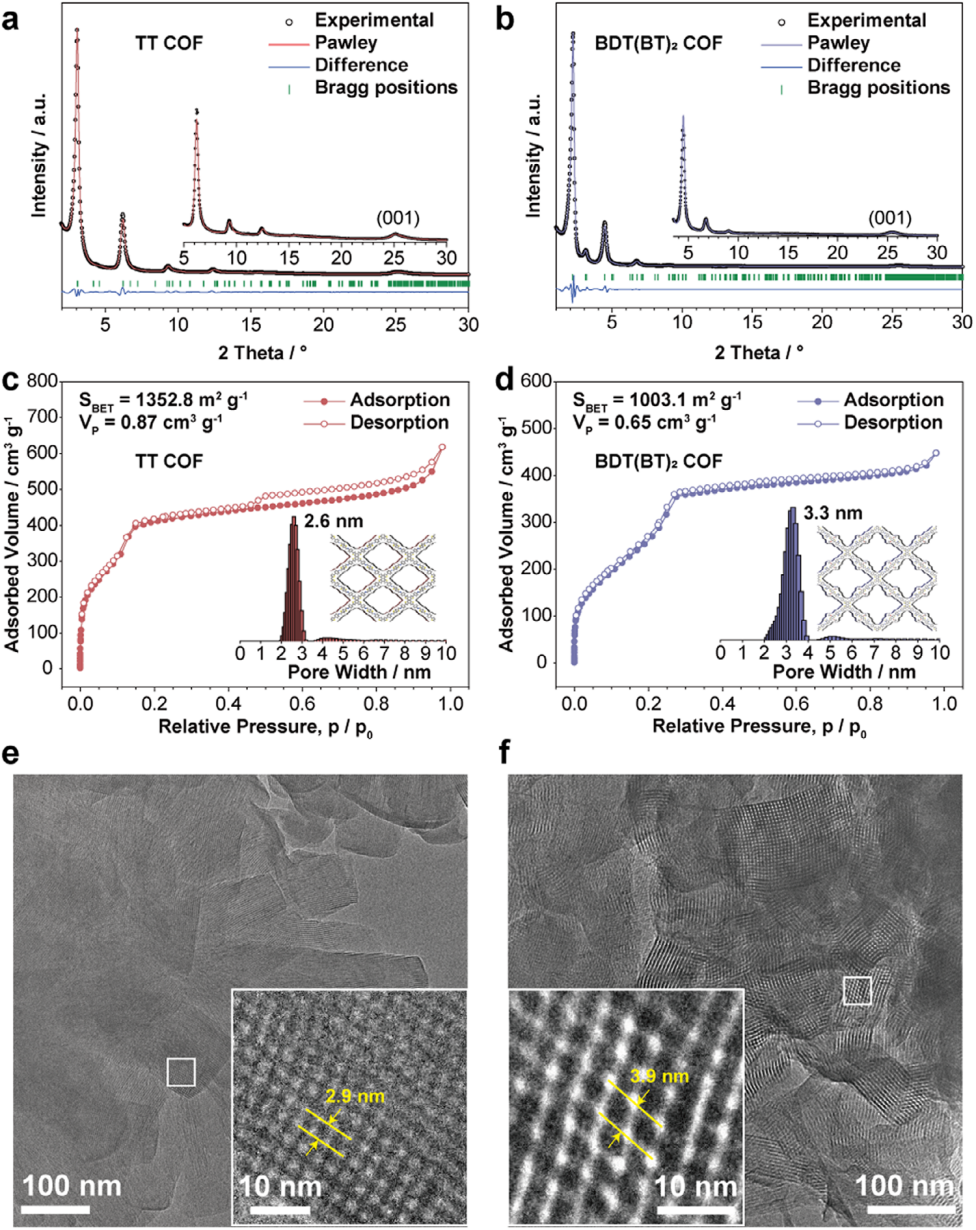
a,b) PXRD patterns of the highly crystalline TT and BDT(BT)_2_ COFs, with simulations based on Pawley refinements. The insets represent Pawley‐refined structure models of TT and BDT(BT)_2_ COFs viewed perpendicular to the crystallographic *a‐b* plane. c,d) Nitrogen sorption isotherms with their corresponding pore size distribution of TT and BDT(BT)_2_ COFs. e,f) HRTEM images of TT and BDT(BT)_2_ COFs. The insets show magnified regions highlighting the clearly resolved lattice fringes of the corresponding frameworks.

The TTNTA‐based COFs synthesis was carried out via acetic acid catalyzed Schiff‐base polycondensation of the respective linear building blocks with TTNTA in different solvent combinations of benzyl alcohol/mesitylene, to afford highly crystalline TT and BDT(BT)_2_ COFs upon heating at 120 °C for 3 days (details in Supporting Information). The crystal structures of the TTNTA COFs were first characterized by powder X‐ray diffraction (PXRD) (**Figure** **1**a,b). The PXRD patterns of both COFs feature sharp and narrow reflections with first reflections at 3.1° and 2.2° 2*θ* for TT and BDT(BT)_2_ COFs, respectively. Simulation of possible crystal structures for both COFs were carried out by applying *C2/m* symmetry according to the symmetry of TTNTA. Pawley refinements using the Forcite module of Materials Studio software provide both very good fits to the experimental data and simulated lattice parameters. The unit cell parameters of a = 46.0 Å, b = 37.9 Å, c = 3.9 Å, α = γ = 90°, β = 64.1° for TT COF and a = 60.8 Å, b = 53.0 Å, c = 3.9 Å, α = γ = 90°, β = 63.6° for BDT(BT)_2_ COF were obtained. For TT COF, the PXRD patterns revealed Bragg reflections centered at low 2*θ* angles of 3.1°, 4.6°, 6.2°, 9.4°, and 12.5° 2*θ* which were indexed as *hkl* (110), (020), (220), (330), and (440). BDT(BT)_2_ COF exhibited shifts in PXRD signals to lower diffraction angles due to its larger unit cell, 2.2°, 3.2°, 4.5°, 6.8°, and 9.0° 2*θ* corresponding to *hkl* (110), (020), (220), (330), and (440). Notably, the peaks observed at 2*θ* >25° in both PXRD patterns corresponding to the interlayer d‐spacings in the *hkl* (001) direction indicate the formation of a more tightly layered structure due to the planar TTN core, in contrast to the related DBC‐based COFs, which show a peak around 2*θ* ≈24.5° (Figure S5, Supporting Information).^[^ ^37^, ^39^, ^40^, ^41^ ^]^

The chemical structures of both COFs were further characterized by Fourier transform infrared (FT‐IR) and solid‐state ^13^C cross‐polarization magic angle spinning (CP‐MAS) NMR measurements. The FT‐IR spectra of TT and BDT(BT)_2_ COFs showed the disappearance of the C═O vibrational band at 1658 and 1680 cm^−1^ with new stretching vibrations at 1580 and 1500 cm^−1^, which can be attributed to the C═N formation (Figure S6, Supporting Information).^[^ ^42^, ^43^ ^]^ Solid‐state ^13^C CP‐MAS NMR further confirmed the formation of imine bonds by the occurrence of characteristic C═N bond signals at ≈150 ppm (Figure S7, Supporting Information).^[^ ^44^, ^45^ ^]^

Scanning electron microscopy (SEM) analysis revealed that both COFs exhibited typical well‐defined crystallites forming large intergrown agglomerates (Figure S8, Supporting Information). In the case of TT COF, the agglomerated nanorods consist of faceted prismatic crystals with quadrilateral cross‐sections. High resolution transmission electron microscope (HRTEM) images of both COFs indicate the exceptionally high structural order of both TT and BDT(BT)_2_ COFs (Figure 1e,f; Figure S9, Supporting Information). As shown in the magnified image insets of Figure 1e,f, the tetragonal pore structures of TT and BDT(BT)_2_ COFs with the view along the crystallographic *c*‐axis show a periodicity of 2.9 and 3.9 nm. These values are in good agreement with the respective structure models.

The porosity of both COFs was examined by nitrogen sorption isotherm measurements at 77 K. Both COFs exhibited typical type‐IV sorption isotherms, which are characteristic of mesoporous materials (Figure 1c,d).^[^ ^46^ ^]^ The desorption hysteresis observed for TT COF (Figure 1c) indicates additional interparticle textural porosity, consistent with its rod‐like morphology with interparticle spacing, in contrast to the smoother and more closely packed crystallites of BDT(BT)_2_ COF (Figure S8, Supporting Information). The Brunauer–Emmett–Teller (BET) surface areas were determined to be 1352 m^2^ g^−1^ for TT COF and 1003 m^2^ g^−1^ for BDT(BT)_2_ COF, and the total pore volumes were estimated to be 0.87 and 0.65 cm^3^ g^−1^, respectively. The pore size distribution analysis based on quenched solid density functional theory (QSDFT) revealed pore sizes of 2.6 and 3.3 nm with a narrow pore size distribution for TT and BDT(BT)_2_ COFs. Additionally, both TTNTA COFs demonstrated high solvent stability, tested via exposing to various solvent conditions (Figure S10, Supporting Information). The thermal stability of both COFs was investigated using thermogravimetric analysis (TGA), revealing that TT and BDT(BT)_2_ COFs are thermally stable up to 410 and 380 °C under the dynamic TGA conditions, respectively (10% weight loss) (Figure S11, Supporting Information).

To elucidate the photophysical and electronic properties of these intriguing COFs, we prepared thin films of the TTNTA‐COFs on glass or indium tin oxide (ITO)‐coated glass substrates using a solvothermal synthesis method. A slide holder with a horizontal placement of the substrate was put into a solution mixture of the precursors and 6 m AcOH as a catalyst, which was sealed and heated at 120 °C for different times depending on the desired thickness of the COF film. The synthetic details can be found in the Supporting Information. Here, TT COF achieves successful growth on both glass and ITO‐coated substrates, thereby demonstrating versatile substrate compatibility. In contrast, BDT(BT)_2_ COF displayed a pronounced preference for growth on ITO‐coated substrates, but with inferior growth quality on glass. We attribute this disparity in growth behavior to differences in the substrate affinity of the linear monomers, which may influence crystal nucleation and film formation processes.

**Figure 2:**
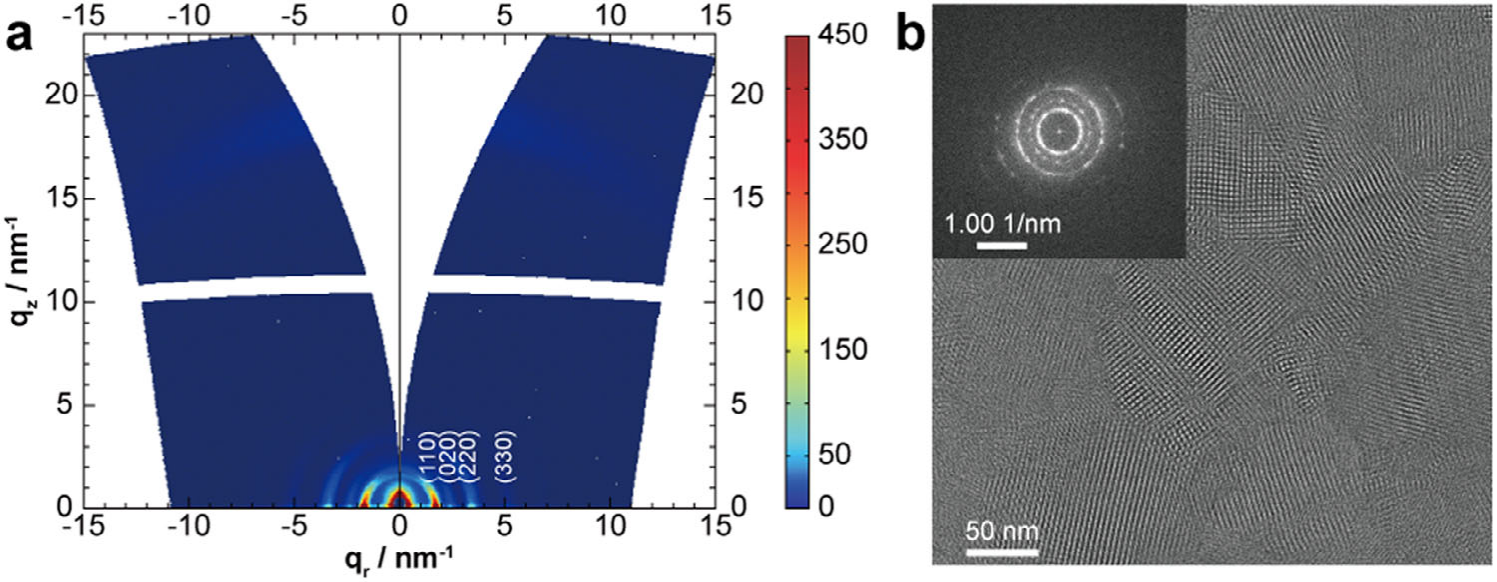
a) GIWAXS 2D pattern of a BDT(BT)_2_ COF thin‐film grown on an ITO‐coated glass substrate. b) HRTEM image and electron diffraction pattern of the BDT(BT)_2_ COF film, obtained by removing it from an ITO‐coated substrate.

Grazing‐incidence wide‐angle X‐ray scattering (GIWAXS) analysis revealed the crystalline structure and preferred orientation of both COF films on the substrates (**Figure** **2**a; Figure S12a, Supporting Information). The strong intensities in the sample horizon (along *q_z_* = 0 nm^−1^) indicate the strongly preferred orientation of the COF 2D lattice planes aligned parallel to and the pore channels tilted away from the substrate surface. Considering morphology, top‐view SEM images of the obtained films display uniform growth on the substrate (Figures S12b and S13a, Supporting Information). TT COF shows a morphology of rod‐like crystals with quadrilateral cross section, ranging from 50 to 100 nm in diameter, growing nearly perpendicular to the substrate. Although the BDT(BT)_2_ COF film reveals a similar morphology, its crystallites are larger in diameter (≈150 nm) and more densely intergrown compared to the TT COF film. Cross‐section SEM images show that the films have a thickness of ≈400 nm for the TT COF and 220 nm for the BDT(BT)_2_ COF (Figures S12c and S13b, Supporting Information). The thickness of the BDT(BT)_2_ COF film can be easily tuned ranging from 120 to 220 nm with reaction times extending from 1 to 3 days. Furthermore, two distinct film growth approaches were employed: a one‐step (8 h) growth and a secondary nucleation (4 + 4 h) growth (experimental details in the Supporting Information). GIWAXS analysis shows that both films display a similar preferred orientation (Figure S14a,b, Supporting Information). Notably, this secondary nucleation strategy also provides an effective route to obtain thicker and more densely packed films (Figure S14c, Supporting Information). HRTEM analysis was further carried out for both COF films by removing them from the substrates, which verified the homogenous growth and highly crystalline nature of the films. For TT COF, a projection with quadrilateral shapes and domain dimensions of ≈80 nm can be observed along the *z*‐direction, revealing its characteristic rhombic pore structure (Figure S15, Supporting Information). In comparison, BDT(BT)_2_ COF film exhibits well‐defined domains with larger lateral sizes ranging from 80 to 150 nm, along with distinct grain boundaries. Notably, well‐resolved rhombic pores are clearly observed within individual domains, confirming the highly ordered COF structure with preferred orientation. (Figure 2b; Figure S16, Supporting Information).

**Figure 3:**
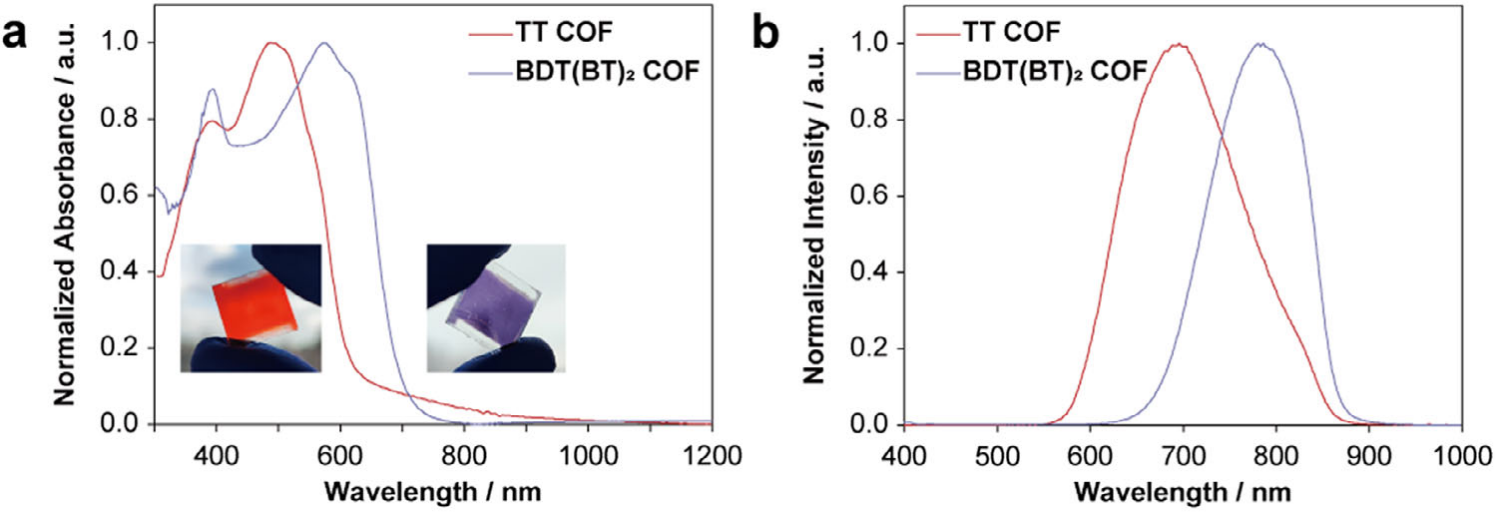
a) UV–vis absorbance and b) PL spectra with 375 nm excitation at room‐temperature of TT COF (red line) and BDT(BT)_2_ COF (purple line) films. The insets represent photographs of both COF films in daylight.

Having succeeded in synthesizing well‐defined thin films of the two TTNTA COFs, we proceeded to elucidate their photophysical properties by means of UV–vis and PL spectroscopy (**Figure** **3**a,b). The homogenous and transparent films of TT and BDT(BT)_2_ COFs exhibit significantly different colors, i.e., bright red and indigo, respectively (inserts in Figure 3a). Both COFs exhibit broad absorption in the ultraviolet and visible regions. Specifically, the TT COF demonstrates a red‐shifted absorption peak, with respect to TTNTA units, at 490 nm, which confirms the electronic interaction between the TTNTA nodes and the TT linkers. Furthermore, the BDT(BT)_2_ COF shows a significantly more red‐shifted absorption peak at ≈580 nm. This shift can be attributed to the electron push‐pull interactions within the framework. Furthermore, both COFs display an absorption band ≈390 nm, which is likely due to *π–π* ^*^ transitions. Assuming a direct transition, the optical band gaps were estimated from the absorption onsets using Tauc plot analysis, yielding values of 2.09 eV for TT COF and 1.85 eV for BDT(BT)_2_ COF (Figure S17, Supporting Information). Notably, the bandgap of the BDT(BT)_2_ COF is reduced by 0.24 eV relative to the TT COF, reflecting the significant influence of the D–A units in enhancing optical absorption in the visible range and effectively narrowing the bandgap. Furthermore, the BDT(BT)_2_ COF exhibits a red‐shifted PL emission by ≈120 nm upon UV excitation at 375 nm (Figure 3b), attributable to the push‐pull interactions within the framework. The high spatial homogeneity of the COF films was also confirmed by observing uniform PL intensity distributions in both films with 476 nm excitation (Figure S18b, Supporting Information). Time‐correlated single photon counting (TCSPC) measurements, fitted using a stretched‐exponential model, further revealed that the BDT(BT)_2_ COF has longer photoluminescence lifetimes compared to the TT COF, likely due to the slower electron–hole recombination dynamics in the D–A system. This can be attributed to the formation of charge‐transfer excitons in the donor–acceptor framework, which are typically more stabilized and thus exhibit extended lifetimes. (Figure S19, Supporting Information).

To further explore the electronic properties of the COF films, interdigitated gold electrodes were evaporated onto the COF films, which had been grown on pre‐cleaned quartz glass substrates. This enables measuring the in‐plane electrical conductivity, both in the dark and under simulated AM 1.5G sunlight at room‐temperature. The device configuration in which Au electrodes were thermally evaporated onto the TT COF thin‐film is depicted in Figure S20a (Supporting Information), and the detailed measurement procedures are described in the Supporting Information. Due to the unique surface morphology of the COF films (Figure S12b,c, Supporting Information), accurately determining the effective cross‐sectional area through which the current flows is challenging. Therefore, instead of reporting an absolute conductivity value, the highest measured current values at an applied bias of −1 V under both dark and illuminated conditions are reported in Figure S20a and Table S1 (Supporting Information). Notably, a substantial increase in current was observed under illumination, corresponding to an on/off ratio of 2.34, calculated from the slopes of the *I*–*V* curves. To validate the reproducibility of this photoactive behavior, an alternative device architecture was employed in which Au electrodes were first evaporated onto insulating glass substrates, followed by growth of the COF film. This reversed configuration yielded comparable results, further confirming the intrinsic photoactive behavior of the TT COF (Figure S20b and Table S1, Supporting Information). These results showcase the favorable electrical DC conductivity and photoactivity of TT COF. Attempts to undertake analogous conductivity measurements for the BDT(BT)_2_ COF proved unsuccessful, as this COF did not grow uniformly on the quartz glass substrate, as previously discussed.

**Figure 4:**
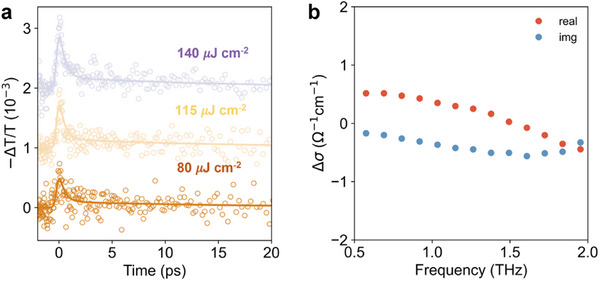
a) Fluence‐dependent OPTP transients in TT COF film, measured following a 3.1 eV pulsed photoexcitation with a pump fluence of 80, 115, and 140 µJ cm^−2^. Solid lines represent fits to a bi‐exponential model. b) Frequency‐resolved complex photoconductivity of TT COF film after photoexcitation (real (red dots) and imaginary (blue dots) components).

To further elucidate charge‐carrier dynamics in these COF films, we measured the time‐resolved photoconductivity using optical‐pump terahertz‐probe (OPTP) spectroscopy. Different from conventional photoconductivity probes, which monitor long‐range photoconductivity between metallic contacts, OPTP provides a noncontact and short‐range measurement of THz photoconductivity with an ultrafast time resolution.^[^ ^47^ ^]^ OPTP transients measured for both TT (**Figure** **4**a) and BDT(BT)_2_ (Figure S21, Supporting Information) COF thin films following 400 nm excitation show a rapid decay of the photoconductivity within ≈1 ps from the photoexcitation. As previously reported for different 2D and 3D COFs,^[^ ^48^, ^49^ ^]^ such ultrafast decay (≈2 ps^−1^ for both COFs, see Table S2, Supporting Information) is associated with the ultrafast formation of excitons. Here, the highly energetic pulsed excitation (400 nm) creates incipient free electron–hole pairs with excess energy, which contribute to the observed photoconductivity. Upon fast cooling, these electron–hole pairs form excitonic states, which are the predominant photophysical species in these materials,^[^ ^50^ ^]^ yet do not contribute to the measured THz photoconductivity. This hypothesis is further confirmed by the significant deviations of the recorded THz photoconductivity spectrum (Figure 4b) from the conventional Drude model. The presence of a non‐zero imaginary photoconductivity component, quantified by a Drude factor^[^ ^51^ ^]^ (see Supporting Information) significantly lower than 1 – here, DF ∼ 0.5 for TT COF – is compatible with the presence of bound electron–hole pairs.^[^ ^52^ ^]^

From the early‐time photoconductivity values (see Supporting Information), we estimated effective charge‐carrier mobility values *φμ*  =  0.34  ±  0.04 cm^2^V^−1^s^−1^ and *φμ*  =  0.18  ±  0.02 cm^2^V^−1^s^−1^for TT and BDT(BT)_2_ COF thin films, respectively (*φ* is the photon‐to‐free‐charge branching ratio for charge carrier generation, *μ* the electron–hole sum mobility). These effective mobility values are consistent with previous reports for other COFs, and their difference can be attributed to a combination of intrinsic factors such as band dispersion, and extrinsic factors, such as packing density and film quality.^[^ ^48^, ^49^ ^]^ We note that the presence of donor–acceptor type moieties in the BDT(BT)_2_ COF does not yield an increase in photoconductivity, thus suggesting that these units do not significantly stabilize charge‐separated states. Although the changes in absorption spectra and photoluminescence dynamics are indicative of push‐pull electronic interactions between D–A moieties, the observed photoconductivity suggests that these changes originate from a change in the nature of excitons (e.g., charge‐transfer character of excitons) rather than from the separation of these excitons into charge‐separated states, therefore not yielding higher free charge‐carrier densities.

**Figure 5:**
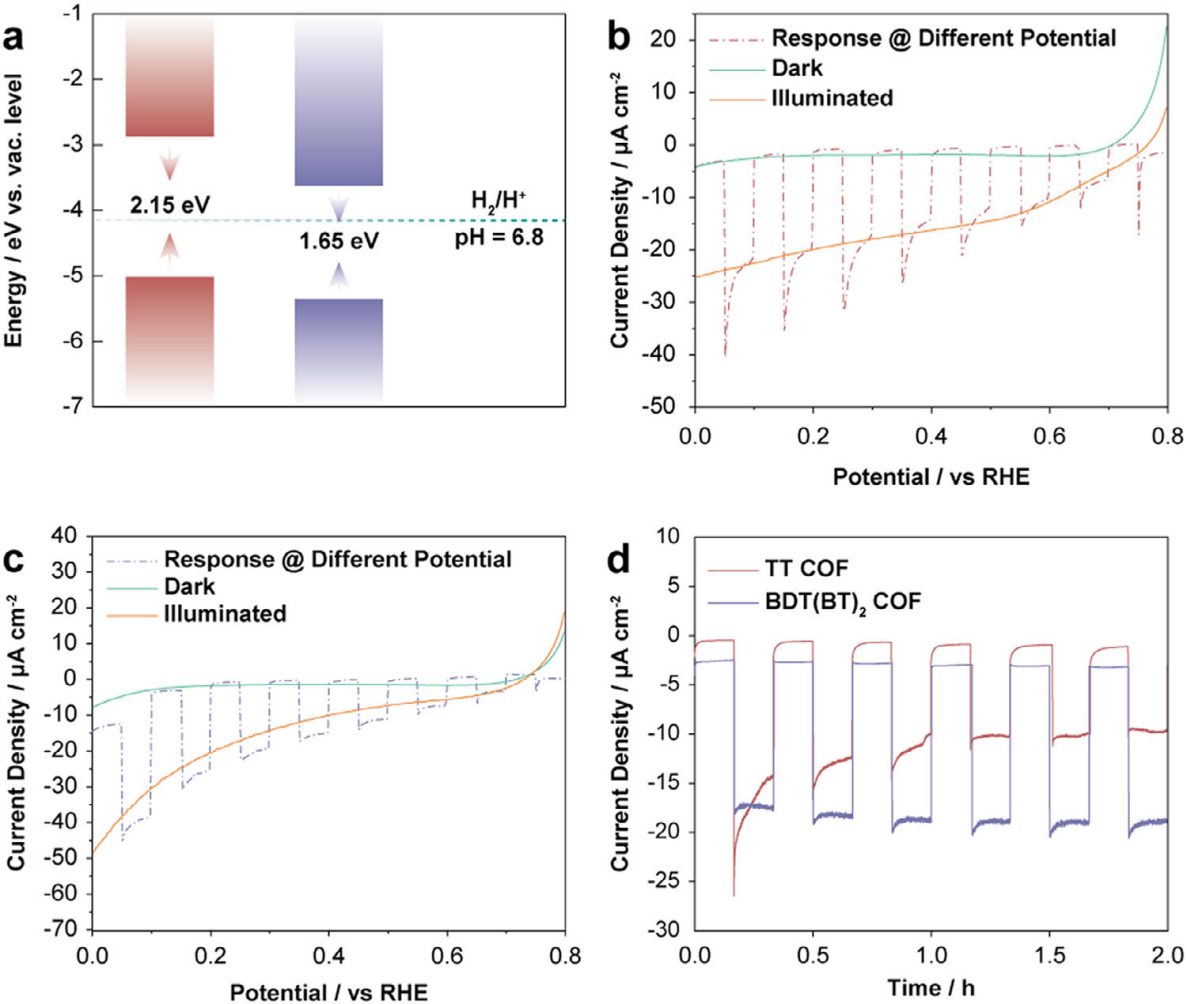
a) Energy levels of the TT and BDT(BT)_2_ COFs versus vacuum. The energy level of H_2_/H^+^ was adapted at pH 6.8 given the Nernstian behavior under standard conditions. Linear sweep voltammetry (LSV) of b) TT and c) BDT(BT)_2_ COF photocathode films on ITO, performed in the dark (green line) and under AM 1.5 G illumination through the substrate (orange line). The red and purple dashed lines inserted in each plot represent the chronoamperometric data of the COF film at different potentials versus RHE. d) Chronoamperometric data of TT and BDT(BT)_2_ COF films at 0.3 V versus RHE.

Based on the optical and electronic properties of the TTN‐based COFs, particularly their strong absorption in the visible region and high charge carrier mobility, we were interested in exploring their electrochemical behavior to harness this potential for photoelectrochemical applications. Cyclic voltammetry (CV) was utilized to determine their HOMO and LUMO energy levels, thereby the onset oxidation and reduction potentials were identified and converted to energy levels relative to the vacuum (Figure S22, Supporting Information). The measurements were conducted in a deoxygenated, anhydrous acetonitrile solution containing 0.1 m NBu_4_PF_6_ as the supporting electrolyte. The HOMO energy levels were determined to be at −5.0 eV for the TT COF and at −5.2 eV for the BDT(BT)_2_ COF, while the corresponding LUMO levels were found to be at −2.85 and at −3.55 eV versus vacuum, respectively. These values suggest that both COFs possess band gaps thermodynamically suitable for photoelectrochemical water splitting (**Figure** **5**a). To assess the photoelectrochemical performance of these COFs, COF films were prepared as electrodes on ITO coated substrate, and linear sweep voltammetry (LSV) as well as photocurrent responses at different potentials were measured in a 0.2 m Na_2_SO_4_ (pH 7) solution. The measurements were conducted using a three‐electrode system under simulated AM 1.5G sunlight, with the potential scanning from 0.8 to 0 V versus the reversible hydrogen electrode (RHE) (Figure 5b,c). Both COF films exhibited an increase in current density as the applied potential decreased over the entire potential range. For further comparison, the photocurrent densities were recorded at a fixed potential of 0.3 V versus RHE, with chopped illumination applied at 10 min intervals. The photocurrent densities of both TT COF and BDT(BT)_2_ COF exhibited periodic on‐off response under illumination, indicating good photoresponse stability over time. The TT COF achieved a photocurrent density of ≈10 µA cm^−2^ after stabilization for intermittent light illumination for 30 min, whereas the BDT(BT)_2_ COF exhibited a significantly higher and more stable photocurrent density, reaching up to 15.3 µA cm^−2^ when compared to the current density in the dark, both of which are among the highest values reported for pure COF‐based photoelectrodes without co‐catalysts.^[^ ^35^, ^50^ ^]^ Notably, the photocurrent of the TT COF decreases with increasing illumination time and stabilizes after 30 min of intermittent illumination. In contrast, the BDT(BT)_2_ COF film exhibited a more stable photocurrent generation over the whole period. Presumably, the initial photocurrent decay of the TT COF originates from the accumulation of photogenerated charge carriers or excitons that fail to separate effectively due to recombination processes or trap states within the material. Prolonged illumination might then have led to the saturation of these trap states, causing the photocurrent to stabilize over time. In contrast, as a photocathode the BDT(BT)_2_ COF demonstrated more efficient charge separation and transport due to its enhanced electronic donor–acceptor character. Apparently, this optimized electronic structure and level alignment minimizes recombination and trap‐state effects, resulting in a more stable photocurrent under prolonged illumination. Notably, both TTNTA‐based COFs exhibit structural stability under prolonged illumination, as confirmed by GIWAXS measurement after intermittent light illumination over a total duration of 1 h (Figure S23, Supporting Information).

Electrochemical impedance spectroscopy (EIS) was conducted to probe the charge‐transfer kinetics at the electrode‐electrolyte interface. The measurements were performed by applying an AC voltage over a frequency range of 1000–0.1 Hz in a 0.5 m Na_2_SO_4_ solution. The Nyquist plots revealed a smaller semicircle for the BDT(BT)_2_ COF, indicative of a lower interfacial charge‐transfer resistance compared to the TT COF (Figure S24, Supporting Information). This reduction in charge‐transfer resistance is consistent with the presence of D–A moieties, which facilitate more efficient charge transport within the BDT(BT)_2_ COF. Notably, this trend appears opposite to the OPTP results, where the TT COF exhibited higher charge carrier mobility. This discrepancy may arise from the different spatial probing dimensions of the two techniques–electrochemical measurements probe across longer transport pathways involving interparticle interfaces, while OPTP primarily reflects local carrier mobility within individual crystallites. Lastly, hydrophilicity is of vital importance for photoelectrochemical hydrogen evolution, in order to optimize wetting of the electrode with water. The BDT(BT)_2_ COF exhibits a smaller water contact angle compared to the TT COF with 89° and 125°, respectively, indicating its more hydrophilic nature and enhanced affinity for water, presumably due to the BT unit's facile hydrogen bond formation with water (Figure S25, Supporting Information). Altogether, these results establish that both TTNTA‐based COFs offer significant potential for water splitting and photocatalysis due to their photoresponsive properties, which facilitate light absorption and charge separation and transfer. While the TT COF may require optimization to mitigate photocurrent decay, the stable photocurrent of BDT(BT)_2_ COF highlights its robustness for sustained photocatalytic applications.

## 3 Conclusion

Summarizing, this work presents a novel and facile synthetic strategy toward incorporating thiophene‐rich TTN as a versatile node for the synthesis of 2D COFs. The successful integration of the multiple thiophene‐containing units into 2D COF structures opens up new possibilities for the design and synthesis of thiophene‐based COFs with promising optoelectronic properties. Through the utilization of these nodes, we synthesized an almost purely thiophene‐based TT COF as well as a BDT(BT)_2_ COF with an integrated D–A push‐pull electronic architecture, both of which demonstrate favorable interlayer stacking characteristics due to the highly planar TTN core. Furthermore, we successfully fabricated homogeneous, highly crystalline, and oriented thin films of both COFs, enabling a detailed investigation of their optical and electronic properties. Ultrafast OPTP measurements reveal that both COF films exhibit notable photoactivity and remarkable charge carrier mobilities in the field of 2D COFs, highlighting their potential for electronic and optoelectronic applications. In addition, both COF films demonstrated exceptionally high photocurrent densities in photoelectrochemical water splitting, ranking among the highest reported for pure COF films. Thereby, the D–A type BDT(BT)_2_ COF exhibited enhanced performance, likely due to its improved charge separation and transport properties. These findings underscore the potential of thiophene‐enriched COFs as promising candidates for photo(electro)chemical catalysis and other light‐driven applications.

## Conflict of Interest

The authors declare no conflict of interest.

## Supporting information



Supporting Information

## Data Availability

The data that support the findings of this study are available in the supplementary material of this article.
