# Supplementary material for: Photoactive Thiophene‐Enriched Tetrathienonaphthalene‐Based Covalent Organic Frameworks
Source: Small. 2025 Nov 7;21(49):e11000. doi: 10.1002/smll.202511000 (PMC12696777; doi:10.1002/smll.202511000)
Supplement: Supplementary file 1 — Supporting Information [file SMLL-21-e11000-s001.docx]

**Supporting Information**

**Photoactive Thiophene-enriched Tetrathienonaphthalene-based Covalent Organic Frameworks**

Tianhao Xue,^a^ Marcello Righetto,^b^ Roman Guntermann,^a^ Shizhe Wang,^a^ Dominic Blätte,^a^ Zehua Xu,^a^ Andreas Weis,^a^ Ignacio Munoz-Alonso,^a^ Dana D. Medina,^a^ Achim Hartschuh,^a^ Laura M. Herz^b^ and Thomas Bein^a^*

^a^Department of Chemistry and Center for NanoScience (CeNS), Ludwig-Maximilians-Universität (LMU), Butenandtstraße 5-13, 81377 Munich, Germany

^b^Department of Physics, Clarendon Laboratory, University of Oxford, Oxford OX1 3PU, U.K.

**Table of contents**

1. Methods 3

2. Materials and Building block synthesis 8

2.1 Materials 8

2.2 Building block synthesis 9

2.3 COF Synthesis 15

3. Characterization 18

3.1 NMR Spectroscopy of Building Blocks 18

3.2 Density Functional Theory (DFT) Calculations and Optical Properties of TTNTA 25

3.3 Cyclic Voltammograms (CV) of TTNTA 26

3.4 FT-IR Spectra 27

3.5 Solid-state ^13^C Cross-Polarization Magic Angle Spinning (CP-MAS) NMR 27

3.6 Scanning Electron Microscopy (SEM) of COF Powders 28

3.7 High-Resolution Transmission Electron Microscopy (HRTEM) of COF Powders 28

3.8 Solvent Stability Evaluation of COF Powders 29

3.9 Thermogravimetric Analysis 29

3.10 Grazing-Incidence Wide-Angle X-ray Scattering (GIWAXS) and SEM of COF Films 30

3.11 HRTEM of TT and BDT(BT)_2_ COF Films 32

3.12 Optical Properties of COF Films Characterized by UV-Vis and PL Spectroscopy 33

3.13 In-Plane Electrical Conductivity Measurement of TT COF Film 35

3.14 Extraction of charge-carrier mobility from OPTP measurements 36

3.15 CV Analysis of COF Films 39

3.16 GIWAXS Measurement of COF Films After Illumination 40

3.17 Electrochemical Impedance Spectroscopy (EIS) Analyses of COF Films 40

3.18 Surface Wettability of COF Films Assessed by Water Contact Angle Measurements 41

3.19 Structural analysis 41

4. References 44

1. Methods

**Nuclear magnetic resonance** (NMR) spectra were recorded on Bruker AV 400 and AV 400 TR spectrometers. Proton chemical shifts are expressed in parts per million (δ scale) and are calibrated using residual undeuterated solvent peaks as an internal reference (CDCl_3_: 7.26, DMSO-*d_6_*: 2.50). Data for ^1^H NMR spectra are reported in the following way: chemical shift (*δ* ppm) (multiplicity, coupling constant/Hz, integration). Multiplicities are reported as follows: s = singlet, d = doublet, t = triplet, q = quartet, m = multiplet, br = broad, or combinations thereof. Magic angle spinning (MAS) solid-state nuclear magnetic resonance (ssNMR) spectra were recorded using a Bruker Avance III-500 spectrometer.

High resolution electron ionization (EI) **mass spectra** (MS) were recorded with a Thermo Finnigan MAT 95 instrument.

**Powder X-ray diffraction** (PXRD) measurements were performed using a Bruker D8 Discover instruments with Ni-filtered Cu Kα radiation and a LynxEye position-sensitive detector.

The **structure models** **of the TT COF and BDT(BT)_2_ COF** were built using the Forcite module of the Accelrys Materials Studio software package. We applied the space group *C2/m* as the possible symmetry. Using this initial coarse model, we determined the unit cell parameters *via* Pawley refinement of our experimental PXRD data.

**Transmission electron microscopy (TEM)** was performed on an FEI Titan Themis equipped with a field emission gun operated at 300 kV.

**Scanning electron microscopy (SEM)** images were recorded with an FEI HeliosNanoLab G3 UC scanning electron microscope equipped with a field emission gun operated at 3-5 kV.

**Fourier-transform infrared spectroscopy** (FT-IR) measurements were performed with a Bruker Vertex 70 FTIR instrument by focusing light of a globar (silicon carbide) as MIR light source through a KBr beam splitter with integrated gold mirrors and an ATR sample stage with a Ge crystal. The spectra were recorded by a N_2_ cooled MCT detector with a resolution of 1 cm^−1^ and averaged over 1000 scans.

The **nitrogen sorption** isotherms were recorded on a Quantachrome Autosorb 1 at 77 K in a pressure range from *p/p_0_* = 0.001 to 0.98. Prior to the measurement of the sorption isotherm the samples were outgassed for 24 h at 120 °C under high vacuum. The calculations for obtaining the pore size distributions were performed using the QSDFT equilibrium model with a carbon kernel for cylindrical pores.

**Thermogravimetric analysis** (TGA) measurements were performed using a Netzsch Jupiter ST 449 C instrument equipped with a Netzsch TASC 414/4 controller. The samples were heated from room temperature to 800 °C under a synthetic air flow (25 ml min^-1^) at a heating rate of 10 K min^-1^.

**2D grazing‐incidence wide angle** **X‐ray scattering** data were recorded with an Anton Paar SAXSpoint 2.0 system equipped with a Primux 100 micro Cu K_α_ source and a Dectris EIGER R 1M 2D detector. The COF films were positioned at a sample-detector distance of 140 mm and were measured with an incidence angle of 0.2°.

**UV-Vis-NIR** **spectra** were recorded using a Perkin-Elmer Lambda 1050 spectrometer equipped with a 150 mm integrating sphere, photomultiplier tube (PMT) and InGaAs detector.

**Photoluminescence (PL)** data were obtained with a FluoTime 300 instrument from PicoQuant GmbH. The samples were photo-excited using a laser with 375 nm wavelength (LDH-P-C-375 from PicoQuant GmbH) pulsed at 500 kHz, with a pulse duration of ~100 ps and fluence of ~300 nJcm^−2^/pulse. The samples were exposed to the pulsed light source set at 3 μJ cm^−2^/pulse fluence for ~10 minutes prior to measurement to ensure stable sample emission. The PL was collected using a high-resolution monochromator and photomultiplier detector assembly (PMAC 192-N-M, PicoQuant GmbH).

For **confocal photoluminescence (PL) measurements**, hyperspectral images and time resolved PL images, a home-built confocal laser scanning microscope was used. It is based on a microscope body (NIKON) which is combined with an xyz-piezo-scanning stage (PHYSIK INSTRUMENTE). The samples were measured upside down in epi-direction with an air objective (0.85 NA, NIKON). A beamsplitter (MELLES GRIOT 03BTL005) and a spectral 490 nm long pass filter was used to separate the laser from the PL emission. A sub picosecond laser (ichrome TOPTICA), which is tunable from 476 nm to 645 nm, was used for excitation. Here we measured only with the 476 nm laser light that was additionally filtered by a band pass 473/10 nm (CHROMA) in the excitation arm. The detection side consists of two parts, which are separated by a flippable mirror. One has an avalanche photo diode (APD, type: MPD PDM, detector size 50 x 50 µm), which can be combined with a Time Correlated Single Photon Counting (TCSPC) electronics (BECKER UND HICKEL) measuring time resolved PL-transients. The second part consists of a spectrometer (ANDOR SHAMROCK SRi303) combined with an open electrode CCD camera (ANDOR NEWTON DU920) recording the spectrum. The data were recorded using a customized LABVIEW (NATIONAL INSTRUMENTS) program that combines the manufacturers’ software to control our desired measurements. Further processing and analysis were carried out using a MATLAB (MATHWORKS) program to obtain the PL spectra, TCSPC transients and the images.

**In-Plane Conductivity Measurements**

The in-plane electrical conductivity of thin films deposited on insulating glass substrates was measured. Gold (Au) contacts were deposited onto the films *via* thermal evaporation through a shadow mask featuring an interdigitated electrode pattern (Figure S20 (a)). For the reverse device configuration, Au was first deposited through the same interdigitated shadow mask onto the pre-cleaned insulating glass substrates, and subsequently, the COF thin film was synthesized on top *via* a solvothermal method (Figure S20 (b)). Measurements were taken using a Newport OrielSol 2A solar simulator with a Keithley 2401 source meter, calibrated to the intensity of 1 sun, or under dark conditions through a voltage sweep between -1 V and 1 V.

**Optical pump terahertz probe (OPTP)** measurements were performed using a setup described in full detail elsewhere.^[1]^ Briefly, an amplified Ti:sapphire laser system (Spitfire ACE, SpectraPhysics) provides 800-nm pulses with 5-kHz repetition rate and 35-fs pulse duration. This fundamental output is used to generate single-cycle THz radiation pulses in a spintronic emitter (W/Co_40_Fe_40_B_20_/Pt multilayer film on quartz) through the inverse spin Hall effect.^[2]^ Furthermore, 400-nm pulses used for photoexciting the thin film samples are generated in a beta-barium-borate (BBO) crystal by second-harmonic generation. In the measurements, fractional changes in the THz transmission (0.5-2.5 THz range of interest) following the 400-nm photoexcitation are monitored by using free-space electro-optic sampling (EOS) in a 1-mm-thick (110)-ZnTe crystal. We measured COF thin films deposited onto 2 mm thick z-cut quartz. During OPTP measurements, the THz emission and detection optics and samples are kept under vacuum at pressures below 0.1 mbar.

**Electrochemical measurements** were performed with a PGSTAT potentiostat/galvanostat equipped with an FRA32M module. **Electrochemical impedance spectroscopy** (EIS) analyses were performed in a three-electrode configuration with a reversible hydrogen electrode (RHE) as reference electrode, Pt wire as counter electrode and COF films on ITO substrate as working electrode over the frequency range of 1000 - 0.1 Hz with an applied perturbation voltage of 10 mV. The electrolyte was 0.5 M Na_2_SO_4_ in deionized water. **Cyclic voltammetry** (CV) scans for determining HOMO and LUMO energy levels were recorded in a three-electrode configuration with Ag/Ag^+^ as reference electrode, Pt wire as counter electrode and a COF coated ITO substrate as working electrode. Anhydrous acetonitrile, containing 0.1 M tetrabutylammonium hexafluorophosphate, was used as supporting electrolyte. All potentials are referenced to a measured fc/fc^+^ redox pair (*E*_fc/fc+_ = +0.55 V vs. Ag/Ag^+^) and were calibrated against fc/fc^+^ (–5.10 eV versus vacuum level).

**Photoelectrochemical measurements** were carried out in a custom build, airtight cell with quartz glass window under constant nitrogen purging to remove any dissolved oxygen in the electrolyte.^[3]^ The electrolyte was 0.2 M Na_2_SO_4_ in deionized water. Linear sweep voltammetry (LSV) scans were performed against reversible hydrogen electrode (RHE) and with a scan rate of 10 mV s^–1^ in the dark as well as under AM1.5 G illumination through the substrate by a solar simulator at 100 mW cm^2^. **Chronoamperometric** measurements on COF photoelectrodes were performed in the same airtight cell filled with 30 mL of the same aqueous Na_2_SO_4_ solution that was used for LSV. The potential was set to constant 0.3 V vs. RHE for all measurements.

2. Materials and Building block synthesis

2.1 Materials

All reagents and solvents were obtained from in-house supply or commercial suppliers and used as received. 1,4-dioxane (anhydrous, Sigma Aldrich), tetrahydrofuran (THF, anhydrous, Sigma Aldrich), chlorobenzene (for synthesis, Sigma Aldrich), acetic acid (in-house supply), benzyl alcohol (BnOH, anhydrous, Sigma-Aldrich), mesitylene (anhydrous, Sigma-Aldrich), di-2-thienyl ketone (98%, ABCR), titanium(IV) chloride solution (1.0 M in methylene chloride, Sigma Aldrich), zinc dust (<10 μm, ≥98%, Sigma Aldrich), pyridine (anhydrous, Sigma Aldrich), N-bromosuccinimide (Sigma Aldrich), nitromethane (Sigma-Aldrich), iron(III) chloride (anhydrous, 98%, Alfa Aesar), 4-aminophenylboronic acid pinacol cyclic ester (>98%, TCI), potassium carbonate (K_2_CO_3_, 99.995% trace metals basis, Sigma-Aldrich), tetrakis(triphenylphosphine)palladium(0) (Pd(PPh_3_)_4_, >99%, Sigma-Aldrich), benzo[1,2-*b*:4,5-*b’*]dithiophene-4,8-dione (>97%, BLD pharm), ethyl p-toluenesulfonate (Sigma-Aldrich), lithium diisopropylamide (2.0 M in heptane/THF/ethylbenzene, Sigma-Aldrich), trimethyltin chloride solution (1.0 M in THF, Sigma-Aldrich), 7-bromo-2,1,3-benzothiadiazole-4-carboxaldehyde (>97%, BLDpharm), bis(dibenzylideneacetone)palladium(0) (Sigma-Aldrich), tri(o-tolyl)phosphine (>97%, TCI), thieno[3,2-*b*]thiophene-2,5-dicarboxaldehyde (TT, >96%, Sigma-Aldrich).

2.2 Building block synthesis

The synthesis procedures for **TTNTA** and **BDT(BT)_2_-CHO** are shown in Scheme S1 and Scheme S2, respectively. Synthesis of tetra(thien-2-yl)ethene (**TTE**), 2,7,10,15-1,1,2,2-tetrakis(5-bromothiophen-2-yl)ethene (**TTE-4Br**), and 4,8-diethoxybenzo[1,2-*b*:4,5-*b*']dithiophene (**BDT-OEt**) were modified according to literature procedures.^[4-6]^


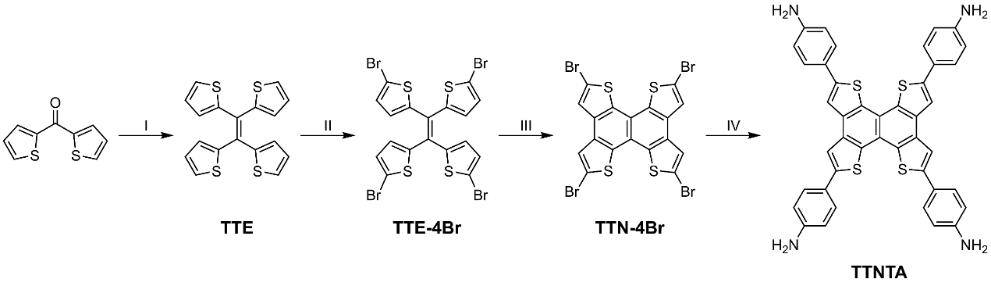


**Scheme S1**. The synthesis route for TTNTA.

**Tetra(thien-2-yl)ethene (TTE).^[4]^**

**
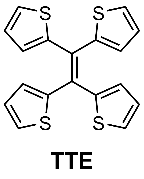
**

A 250 mL two-necked flask equipped with anhydrous tetrahydrofuran (THF, 100 mL) was kept in an ice-water bath while vigorously stirring under nitrogen atmosphere. A solution of 1.0 M TiCl_4_ in dichloromethane (15 mL, 15 mmol) was added dropwise into the above THF solution, resulting in a clear pale orange solution. After 15 min, small portions of zinc powder (1.8 g, 27.6 mmol) and pyridine (1.2 mL, 15.0 mmol) were sequentially added into the above solution. After stirring for 2 hours at room temperature, a solution of di-2-thienyl ketone (2.5 g, 12.9 mmol) in 20 mL THF was added. The reaction mixture was refluxed at 80 °C overnight, resulting in a brick-red reaction mixture. After cooling to room temperature, the reaction was quenched with saturated aqueous K_2_CO_3_ solution and extracted with ethyl acetate and further washed with saturated NaCl solution. The organic layer was collected and dried over anhydrous MgSO_4_, then the solvent was removed under reduced pressure. Further purification was carried out with column chromatography (silica gel, pure DCM) to afford the orange powder of TTE (1.2 g, 3.4 mmol). Yield: 52%.

**^1^H NMR** (400 MHz, CDCl_3_) *δ /* ppm: 7.31 (dd, 4H), 6.94 (dd, 4H), 6.87 (dd, 4H).

**1,1,2,2-tetrakis(5-bromothiophen-2-yl)ethene (TTE-4Br).^[5]^**


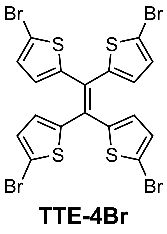


A solution of TTE (1.2 g, 3.4 mmol, 1.0 eq) in 100 mL anhydrous THF was kept in an ice-water bath under nitrogen atmosphere. N-Bromosuccinimide powder (15.1 mmol, 2.7 g, 4.5 eq) was added and the mixture was stirred in the dark at room temperature overnight. The reaction was quenched with H_2_O, extracted with CHCl_3_ and further washed with saturated NaCl solution. The organic layer was dried over anhydrous MgSO_4_, then the solvent was removed under reduced pressure. Further purification was carried out with column chromatography (silica gel, n-hexane/DCM 6:1) to afford the red-orange powder TTE-4Br (2.1 g, 3.2 mmol). Yield: 95%.

**^1^H NMR** (400 MHz, CDCl_3_) *δ /* ppm: 6.93 (d, 4H), 6.66 (d, 4H).

**^13^C NMR** (100 MHz, CDCl_3_) *δ /* ppm: 143.91, 131.08, 129.96, 126.86, 115.87.

**2,5,8,11-tetrabromonaphtho[1,2-*b*:4,3-*b*':5,6-*b*'':8,7-*b*''']tetrathiophene (TTN-4Br).**

**
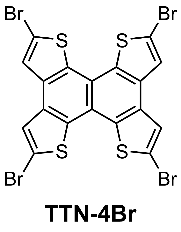
**

A solution of **TTE-4Br** (1 g, 1.50 mmol, 1.0 eq) in 100 mL anhydrous chlorobenzene was kept at 0 °C under nitrogen atmosphere. A solution of FeCl_3_ (3.0 g) in 30 mL nitromethane was prepared and added dropwise to the above solution while vigorously stirring. The reaction was kept at 0 °C for 1 hour, then allowed to warm to room temperature and kept for three days. After the reaction was completed, a 2 M aqueous HCl solution was added dropwise under continuous stirring until an off-white precipitation occurred. The crude product was collected by ﬁltration and washed with water, methanol and CHCl_3_ respectively to afford the off-white product (940 mg, 1.41 mmol). Yield: 95%. Due to the extremely poor solubility, CS_2_ was used as solvent and CDCl_3_ as the reference for the ^1^H NMR measurement to avoid π-π stacking. ^13^C NMR measurements could not be carried out because of the poor solubility.

**^1^H NMR** (400 MHz, CDCl_3_, CS_2_) *δ* / TMS, ppm: 7.88 (s, 4H).

**HRMS-EI:** calculated (m/z): 667.5888, measured (m/z): 667.5752.

**4,4',4'',4'''-(naphtho[1,2-*b*:4,3-*b*':5,6-*b*'':8,7-*b*''']tetrathiophene-2,5,8,11-tetrayl)tetraaniline (TTNTA).**


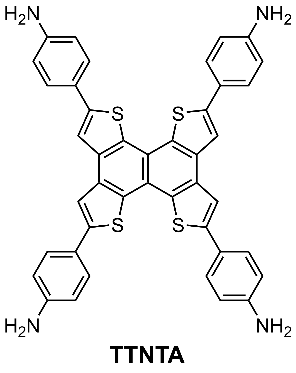


A reaction mixture containing TTN-4Br (powder, 500 mg, 0.75 mmol, 1.0 eq), 4-aminophenylboronic acid pinacol cyclic ester (738 mg, 3.4 mmol, 4.5 eq), K_2_CO_3_ (520 mg, 3.7 mmol, 5 eq), Pd(PPh_3_)_4_ (90 mg, 0.1 mmol, 0.1 eq) in 40 mL 1,4-dioxane and 10 mL H_2_O was refluxed at 115 °C for 72 h with vigorous stirring. After cooling to room temperature, 40 mL H_2_O was added to the solution. The resulting precipitate was collected by filtration and washed with H_2_O and hot methanol to afford the brownish product TTNTA (480 mg, 0.67 mmol). Yield: 90%.

**^1^H NMR** (400 MHz, DMSO-*d_6_*) *δ /* ppm: 8.43 (s, 4H), 7.72 (d, *J* = 8.6 Hz, 8H), 6.75 (d, *J* = 8.6 Hz, 8H), 5.57 (s, 8H).

**^13^C NMR** (101 MHz, DMSO-*d_6_*) *δ /* ppm: 149.63, 145.80, 134.14, 129.74, 127.13, 120.90, 118.75, 116.01, 114.15.

**HRMS-EI:** calculated (m/z): 716.1197, measured (m/z): 716.1181.


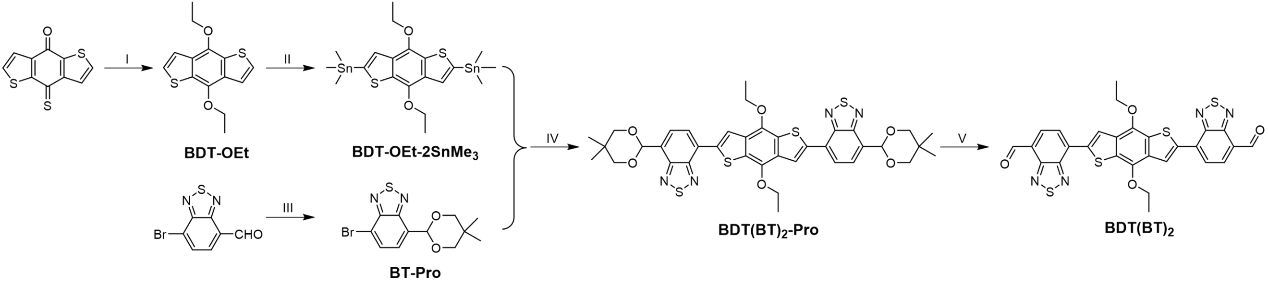


**Scheme S2**. The synthesis route for **BDT(BT)_2_**.

**4,8-Diethoxybenzo[1,2-*b*:4,5-*b′*]dithiophene (BDT-OEt).^[6]^**

**
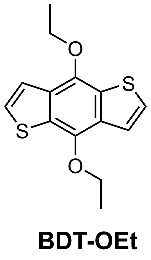
**

Benzo[1,2-*b*:4,5-*b'*]dithiophene-4,8-dione (1.50 g, 6.81 mmol, 1.0 eq) was suspended in a mixture of an aqueous sodium hydroxide solution (20%) (20 mL) and ethanol (20 mL). Under argon atmosphere, 1.34 g (20.43 mmol, 3.0 eq) of zinc dust was added to the above solution and heated at 95 °C for 5 h. 3.50 mL (4.10 g, 20.43 mmol, 3.0 eq) of ethyl p-toluenesulfonate was slowly added and the reaction solution was kept at 95 °C overnight. After cooling to room temperature, 100 mL H_2_O was added, the mixture extracted with CHCl_3_ and further washed with saturated NaCl solution for 3 times. The organic layer was collected and dried over anhydrous MgSO_4_, then the solvent was removed under reduced pressure. Further purification was carried out with column chromatography (silica gel, n-hexane/DCM = from 5:1 to 3:1) to afford the white powder BDT-OEt (1.14 g, 4.08 mmol). Yield: 60%.

**^1^H NMR** (400 MHz, CDCl_3_) *δ /* ppm: 7.48 (d, *J* = 5.5 Hz, 2H), 7.37 (d, *J* = 5.5 Hz, 2H), 4.37 (q, *J* = 7.1 Hz, 4H), 1.49 (t, *J* = 7.0 Hz, 6H).

**^13^C NMR** (101 MHz, CDCl_3_) *δ /* ppm: 144.43, 131.95, 130.52, 126.17, 120.50, 69.60, 16.27.

**(4,8-diethoxybenzo[1,2-*b*:4,5-*b'*]dithiophene-2,6-diyl)bis(trimethylstannane) (BDT-OEt-2SnMe_3_).**


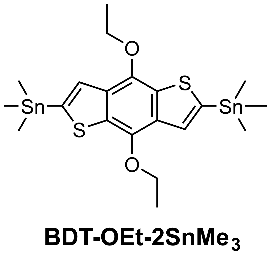


**BDT-OEt** (500 mg, 1.80 mmol, 1.0 eq) was dissolved in 30 mL of anhydrous THF and cooled to -78 °C under an argon atmosphere using standard Schlenk techniques. Lithium diisopropylamide (LDA), (2.0 M in heptane/THF/ethylbenzene, 1.89 mL, 3.78 mmol, 2.1 eq) was added slowly and the resulting mixture was stirred for 1 h at -78 °C after which it was brought to 0 °C for another 30 minutes. Afterwards, trimethyltin chloride (1.0 M in THF, 4.5 mL, 4.5 mmol, 2.5 eq) was added and the solution was stirred overnight, and simultaneously allowed to warm to room temperature. The reaction was quenched with H_2_O, extracted with CHCl_3_ and further washed with saturated NaCl solution. The organic layer was collected and dried over anhydrous MgSO_4_, then the solvent was removed under reduced pressure. The crude product was washed with MeOH and afforded a light yellow solid without further purification. (976.37 mg, 1.62 mmol). Yield: 90%.

**^1^H NMR** (400 MHz, CDCl_3_) *δ /* ppm: 7.52 (s, 2H), 4.39 (q, *J* = 7.0 Hz, 4H), 1.50 (t, *J* = 7.0 Hz, 6H), 0.45 (s, 18H).

**^13^C NMR** (101 MHz, CDCl_3_) *δ /* ppm: 142.99, 140.75, 134.38, 133.28, 128.18, 69.27, 16.31, -8.16.

**HRMS-EI:** calculated (m/z): 603.9725, measured (m/z): 603.9567.

**4-bromo-7-(5,5-dimethyl-1,3-dioxan-2-yl)benzo[*c*][1,2,5]thiadiazole (BT-Pro).**

**
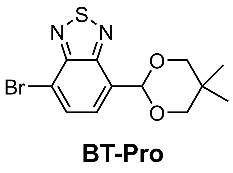
**

7-Bromo-2,1,3-benzothiadiazole-4-carboxaldehyde (1 g, 4.12 mmol, 1.0 eq), 2,2-dimethyl-1,3-propanediol (856.92 mg, 8.23 mmol, 2 eq) and *p*-toluenesulfonic monohydrate (78 mg, 0.41 mmol, 0.1 equiv.) was dissolved in 50 mL anhydrous toluene and refluxed at 110 °C for 4 h under nitrogen atmosphere. After completion of the reaction, the above solution was extracted with DCM. The organic layer was collected and then all the solvent was removed under reduced pressure. n-Hexane was further used to precipitate out and wash the crude to afford the yellow solid as the product. (1200 mg, 3.65 mmol). Yield: 88%. Without further purification, the obtained product was used for the next synthesis step.

**^1^H NMR** (400 MHz, CDCl_3_) *δ /* ppm: 7.89 (d, *J* = 7.6 Hz, 1H), 7.80 (dd, *J* = 7.5, 0.8 Hz, 1H), 6.12 (d, *J* = 0.7 Hz, 1H), 3.87 - 3.78 (m, 4H), 1.35 (s, 3H), 0.84 (s, 3H).

**^13^C NMR** (101 MHz, CDCl_3_) *δ /* ppm: 153.57, 152.54, 132.03, 130.59, 127.50, 115.01, 97.94, 78.07, 30.61, 23.17, 21.99.

**HRMS-EI:** calculated (m/z): 327.9881, measured (m/z): 327.9877.

**7,7'-(4,8-diethoxybenzo[1,2-*b*:4,5-*b'*]dithiophene-2,6-diyl)bis(4-(5,5-dimethyl-1,3-dioxan-2-yl)benzo[*c*][1,2,5]thiadiazole) (BDT(BT)_2_-Pro):**

**
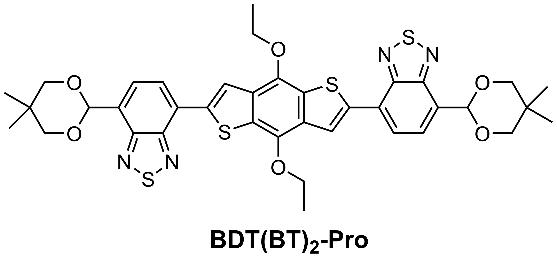
**

In a glove box with argon atmosphere, a mixture of **BDT-OEt-2SnMe_3_** (300 mg, 0.50 mmol, 1.0 eq), **BT-Pro** (360 mg, 1.10 mmol, 2.2 eq), bis(dibenzylideneacetone)palladium(0) (29 mg, 0.05 mmol, 0.1 eq) and tri(o-tolyl)phosphine (61 mg, 0.20 mmol, 0.4 eq) was dissolved in 25 mL anhydrous toluene and refluxed at 110 °C for 3 days. The reaction was quenched with H_2_O, extracted with CHCl_3_ and washed with saturated NaCl solution. Further purification was carried out with column chromatography (silica gel, Pure CHCl_3_) to afford the product as a dark red powder (290 mg, 0.38 mmol). Yield: 75%.

**^1^H NMR** (400 MHz, CDCl_3_,) *δ /* ppm: 8.73 (s, 2H), 7.99 (d, *J* = 0.9 Hz, 4H), 6.22 (s, 2H), 4.53 (q, *J* = 7.0 Hz, 4H), 3.92 - 3.81 (m, 8H), 1.61 (t, *J* = 7.0 Hz, 6H), 1.40 (s, 6H), 0.88 (s, 6H).

**HRMS-EI:** calculated (m/z): 774.1674, measured (m/z): 774.1682.

**7,7'-(4,8-diethoxybenzo[1,2-*b*:4,5-*b'*]dithiophene-2,6-diyl)bis(benzo[*c*][1,2,5]thiadiazole-4-carbaldehyde) (BDT(BT)_2_-2CHO):**

**
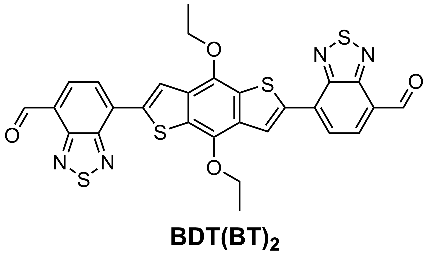
**

To a solution of **BDT(BT)_2_-Pro** (400 mg, 0.52 mmol, 1.0 eq) dissolved in 20 mL CHCl_3_, trifluoroacetic acid (10 mL) and H_2_O (2 mL) were added dropwise subsequently under stirring and left to stir at room temperature for 6 h under argon atmosphere. The reaction mixture was slowly added into saturated NaHCO_3_ solution (60 mL). The resulting precipitate was collected by filtration, followed by washing with MeOH (50 mL) and CHCl_3_ (20 mL) to afford a black powder as the product. After this deprotection procedure, ^1^H and ^13^C NMR measurements could not be carried out due to poor solubility. However, the ^1^H NMR and HRMS-EI results of **BDT(BT)_2_-Pro** from the previous step, as well as the HRMS-EI result shown below provide sufficient evidence for the structure of **BDT(BT)_2_-2CHO**.

**HRMS-EI:** calculated (m/z): 602.0211, measured (m/z): 602.0216.


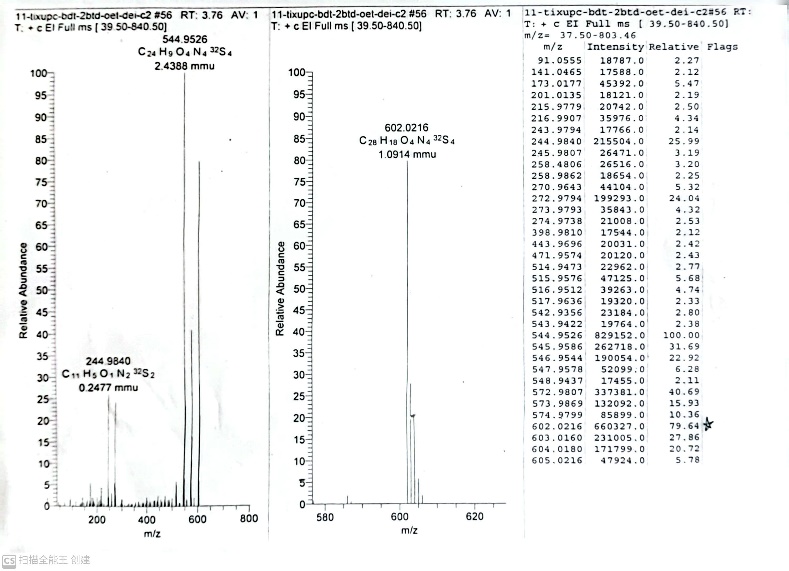


2.3 COF Synthesis


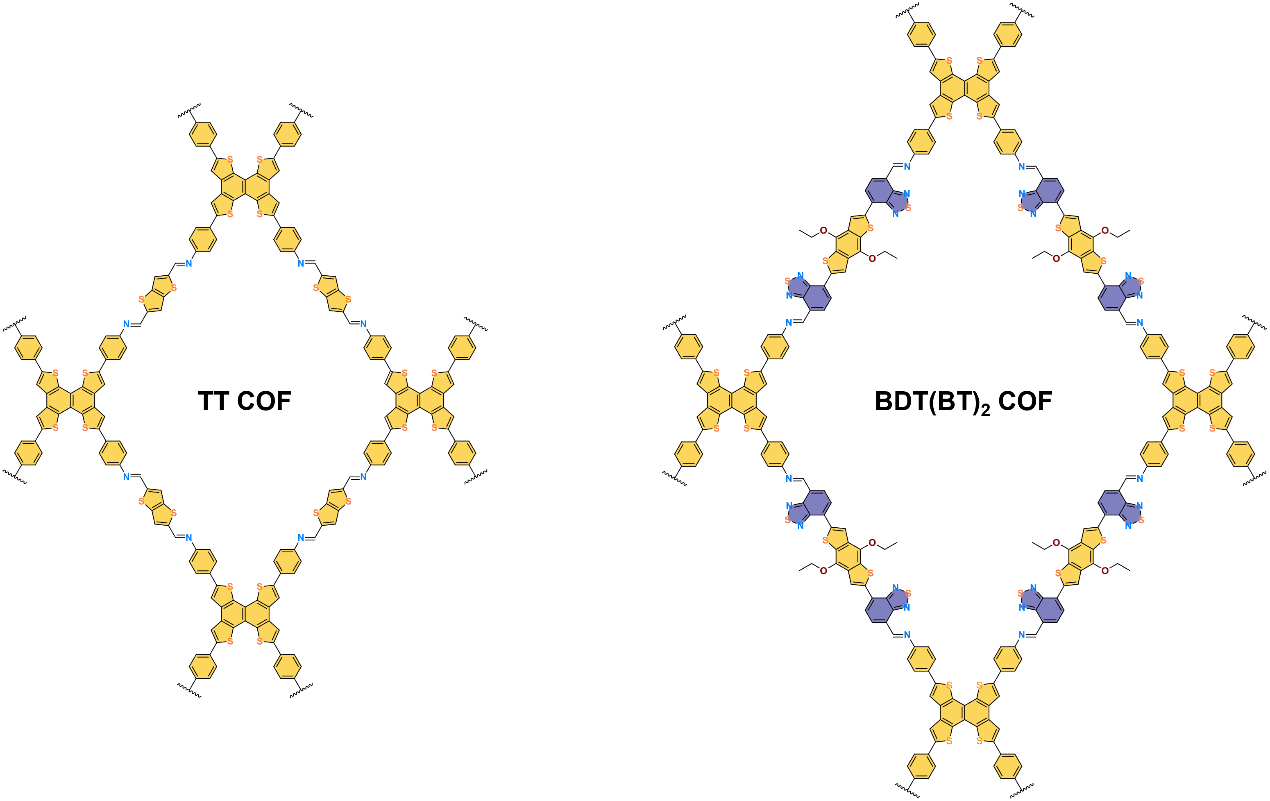


All COF powder syntheses and COF film syntheses were conducted in a glove box with argon atmosphere. Solvents were obtained in high purity grade from commercial suppliers.

**Synthesis of TT COF.** TTNTA (5.4 mg, 7.5 μmol) and thieno[3,2-*b*]thiophene-2,5-dicarboxaldehyde (2.95 mg, 15 μmol) were filled into a 6 mL pyrex tube, followed by addition of BnOH (334 μL), mesitylene (166 μL), and 6 M acetic acid (50 μL). The tube was sealed and heated at 120 °C for 3 days. After cooling to room temperature, the precipitate was collected by filtration and washed with THF before further Soxhlet extraction with THF overnight. After drying under reduced pressure and supercritical CO_2_ extraction, the final product was obtained as dark red powder. (7.1 mg, Yield: 90%)

**Synthesis of BDT(BT)_2_ COF.** TTNTA (5.4 mg, 7.5 μmol) and BDT(BT)_2_-2CHO (9.02 mg, 15 μmol) were filled into a 6 mL pyrex tube, followed by addition of BnOH (167 μL), mesitylene (334 μL), and 6 M acetic acid (50 μL). The tube was sealed and heated at 120 °C for 3 days. After cooling to room temperature, the precipitate was collected by filtration and washed with THF before further Soxhlet extraction with THF overnight. After drying under reduced pressure and supercritical CO_2_ extraction, the final product was obtained as black powder. (12.2 mg, Yield: 88%)

**Synthesis of TT COF film.** TTNTA (5.4 mg, 7.5 μmol) and thieno[3,2-*b*]thiophene-2,5-dicarboxaldehyde (2.95 mg, 15 μmol), were filled into a 25 mL reaction tube, followed by addition of BnOH (1333 μL), mesitylene (667 μL), and 6 M acetic acid (100 μL). Then a glass slide holder with a parallel placement of a glass/ITO substrate was put into this reaction tube, which was sealed and heated at 120 °C for 3 h to 2 days. The slide facing up was cleaned with a cotton swab and then washed with anhydrous THF and sonicated for 5 seconds to remove the residuals. Finally, the film was dried under high vacuum prior to further investigation.

**Synthesis of BDT(BT)_2_ COF film.** TTNTA (1.2 mg, 1.67 μmol) and BDT(BT)_2_ (2 mg, 3.34 μmol), were filled into a 25 mL reaction tube, followed by addition of BnOH (667 μL), mesitylene (1333 μL), and 6 M acetic acid (20 μL). Then a glass slide holder with a parallel placement of a glass/ITO substrate was put into this reaction tube, which was sealed and heated at 120 °C for 1 to 3 days. The slide facing up was cleaned with a cotton swab and then washed with anhydrous THF and sonicated for 5 seconds to remove the residuals. Finally, the film was dried under high vacuum prior to further investigation.

3. Characterization

3.1 NMR Spectroscopy of Building Blocks


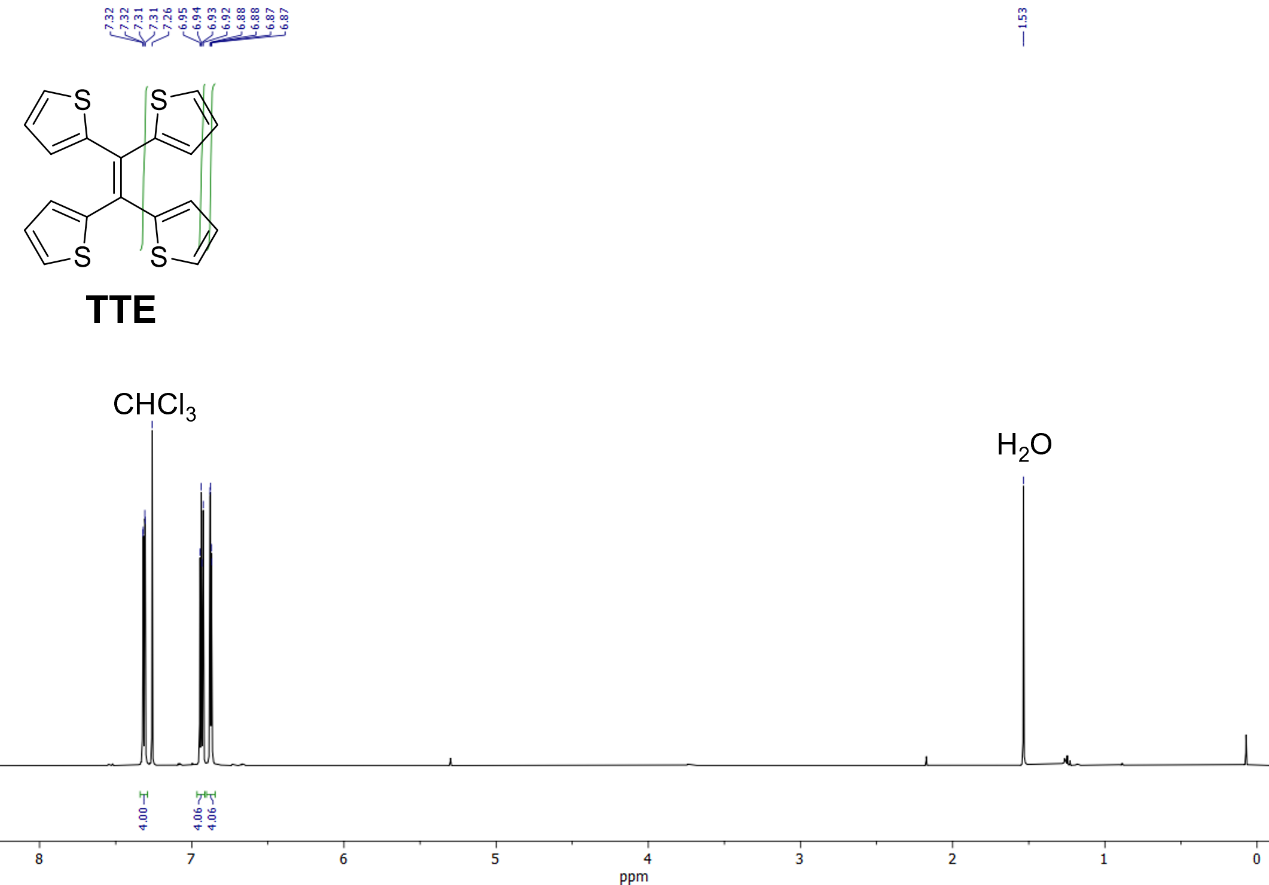


**(a)** ^1^H NMR spectra of **TTE** in CDCl_3_.


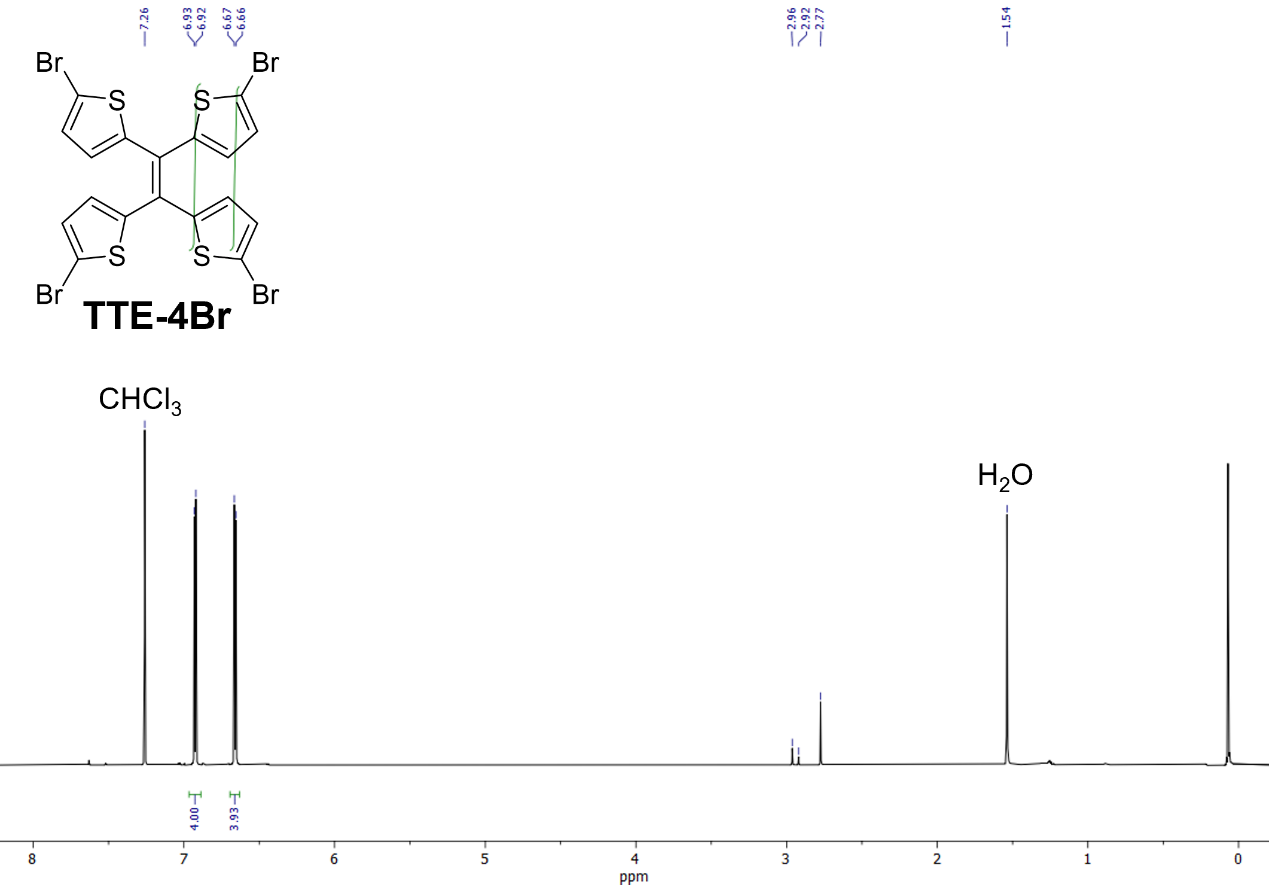


**(b)** ^1^H NMR spectra of **TTE-4Br** in CDCl_3_.


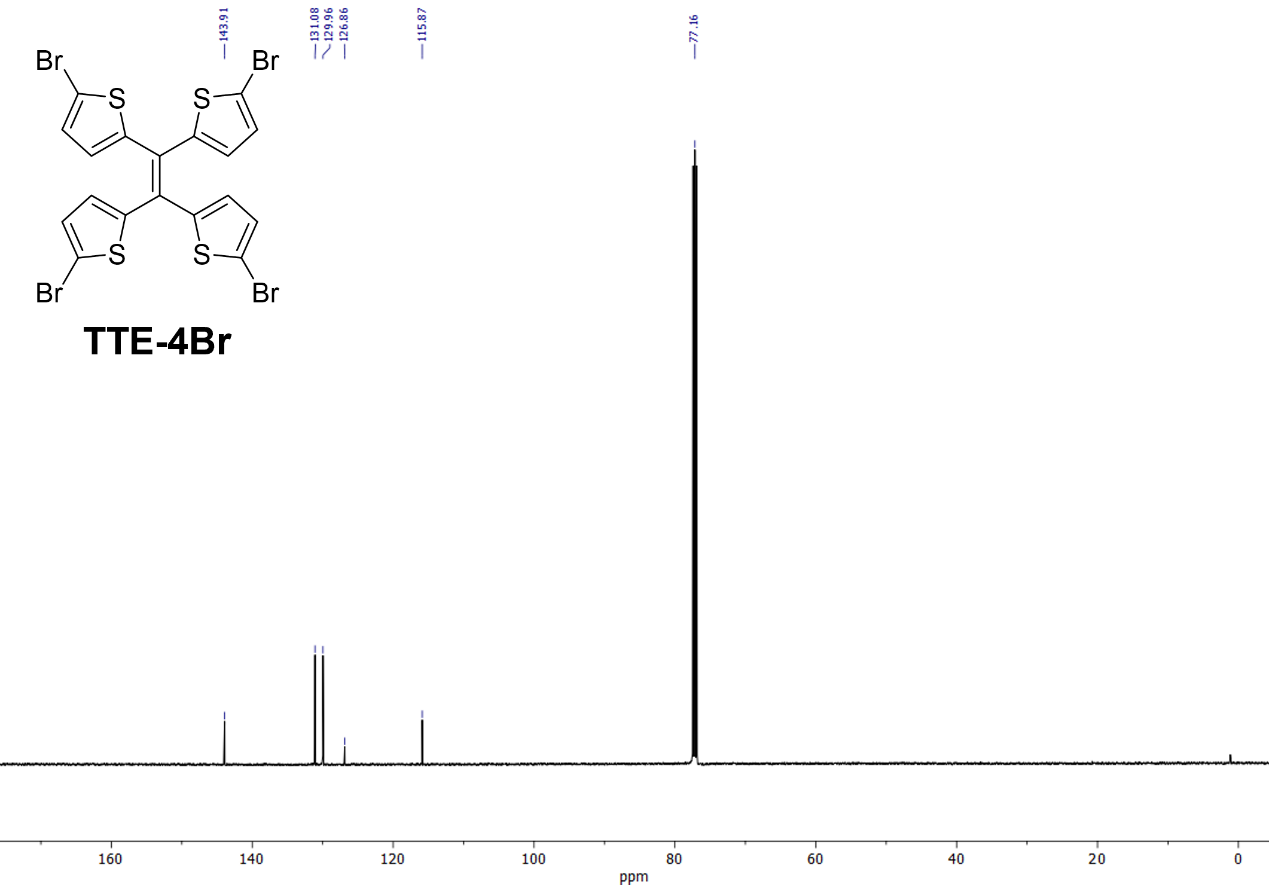


**(c)** ^13^C NMR spectra of **TTE-4Br** in CDCl_3_.


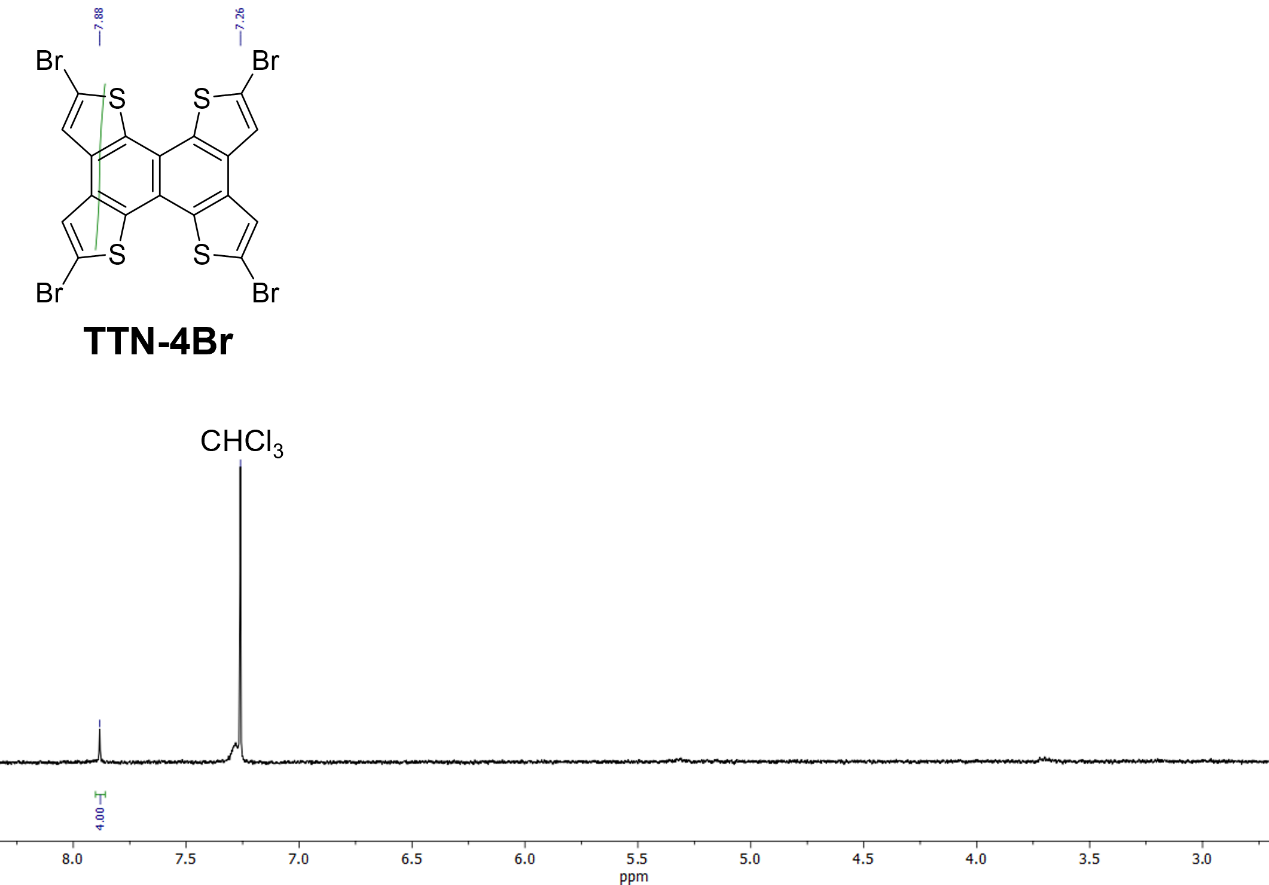


**(d)** ^1^H NMR spectra of **TTN-4Br** in the mixture of CDCl_3_ and CS_2_.


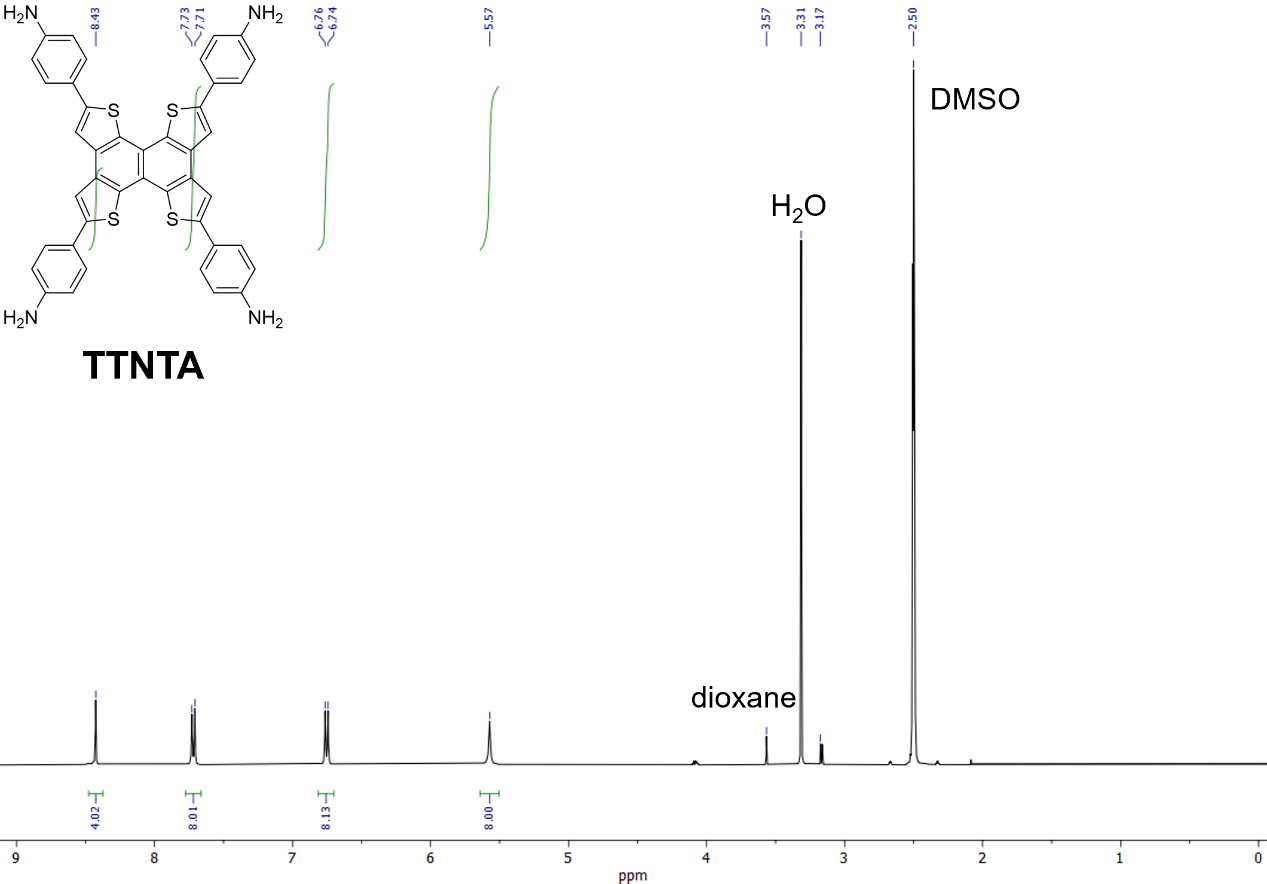


**(e)** ^1^H NMR spectra of **TTNTA** in DMSO-*d_6_*.


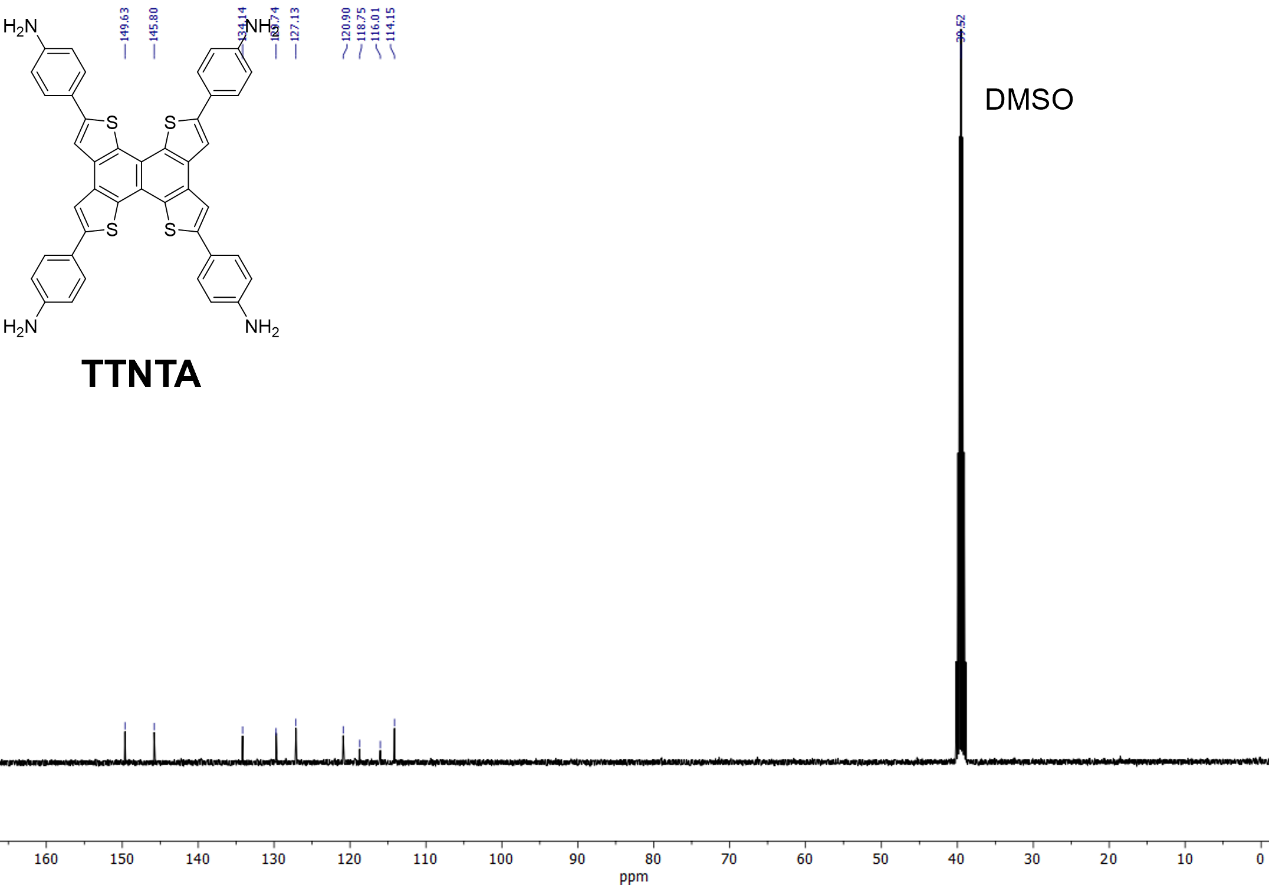


**(f)** ^13^C NMR spectra of **TTNTA** in DMSO-*d_6_*.

**
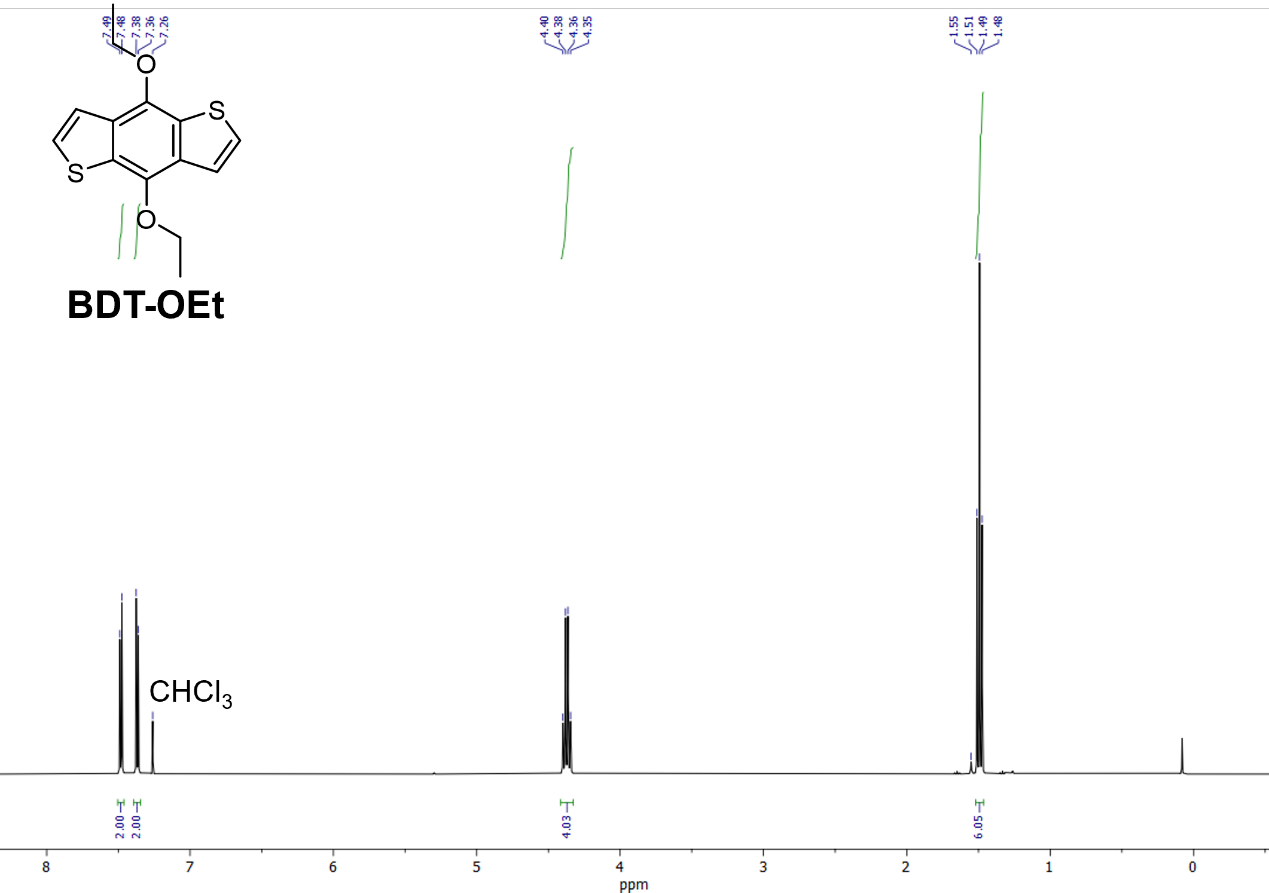
**

**(g)** ^1^H NMR spectra of **BDT-OEt** in CDCl_3_.

**
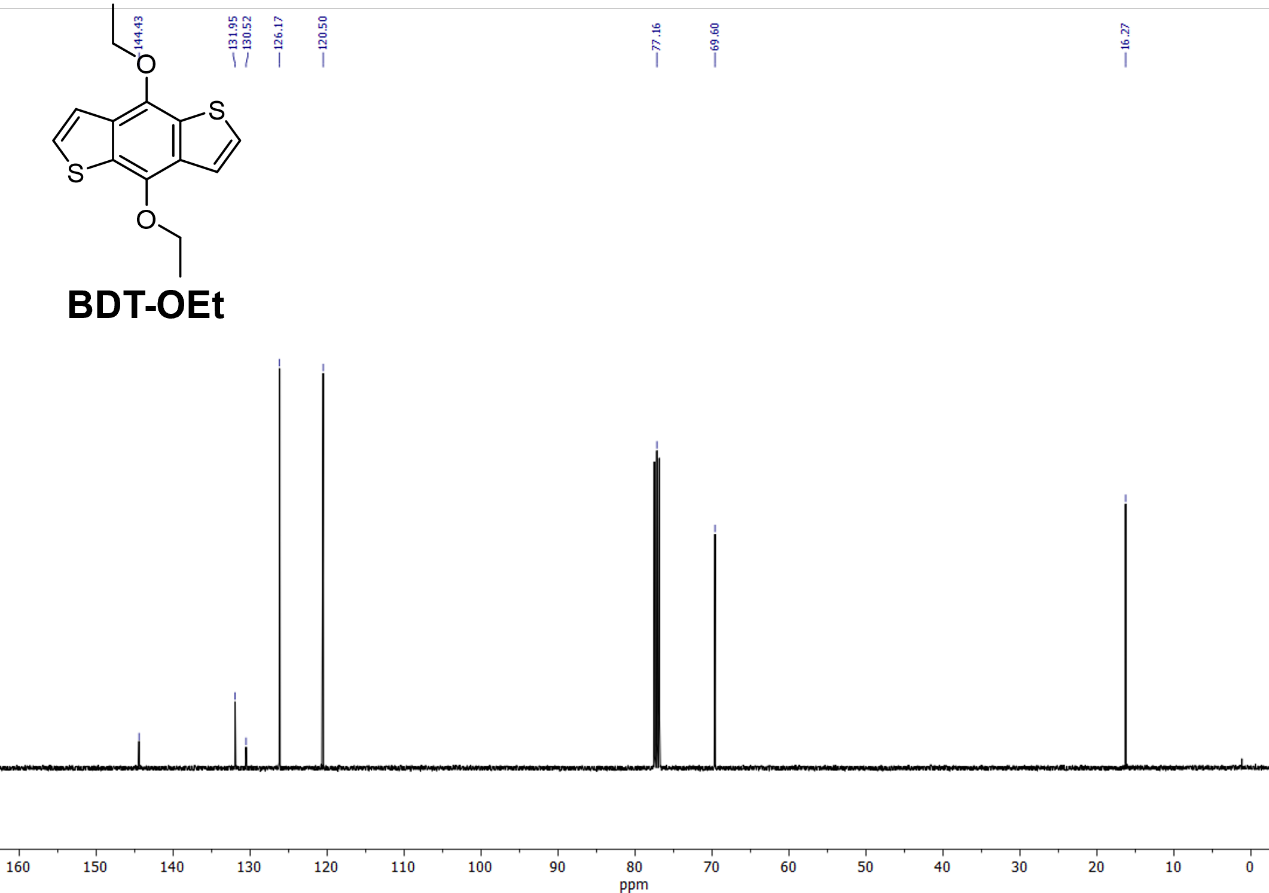
**

**(h)** ^13^C NMR spectra of **BDT-OEt** in CDCl_3_.

**
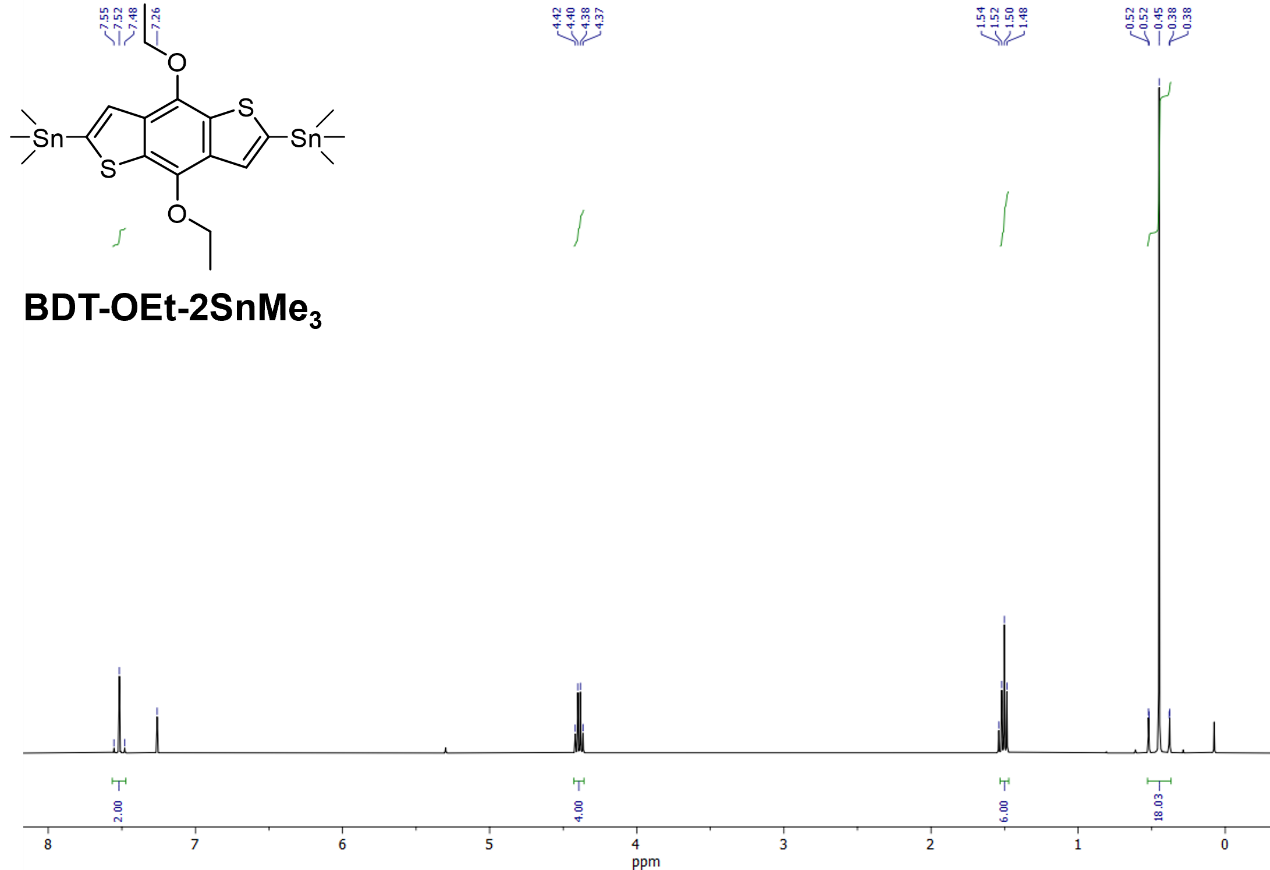
**

**(i)** ^1^H NMR spectra of **BDT-OEt-2SnMe_3_** in CDCl_3_.

**
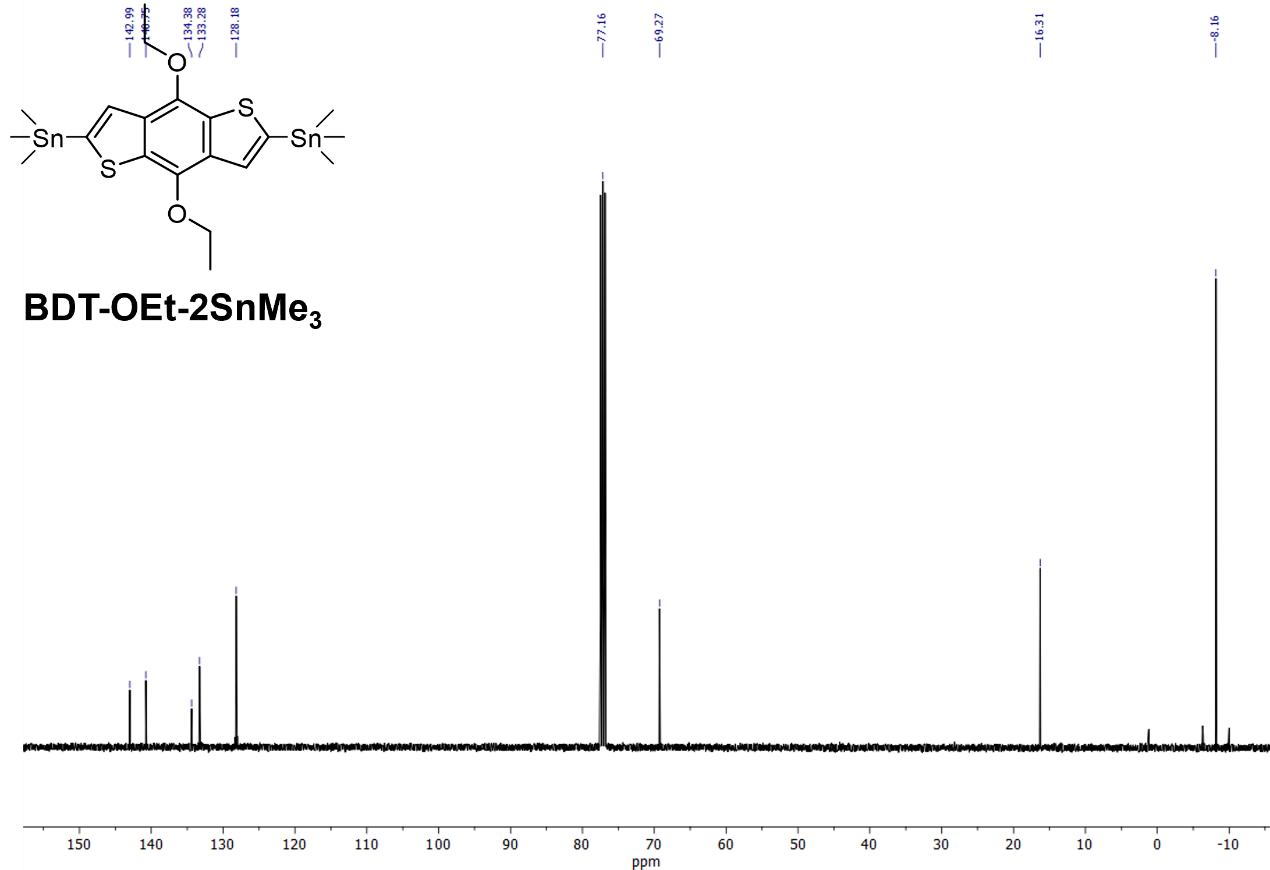
**

**(j)** ^13^C NMR spectra of **BDT-OEt-2SnMe_3_** in CDCl_3_.

**
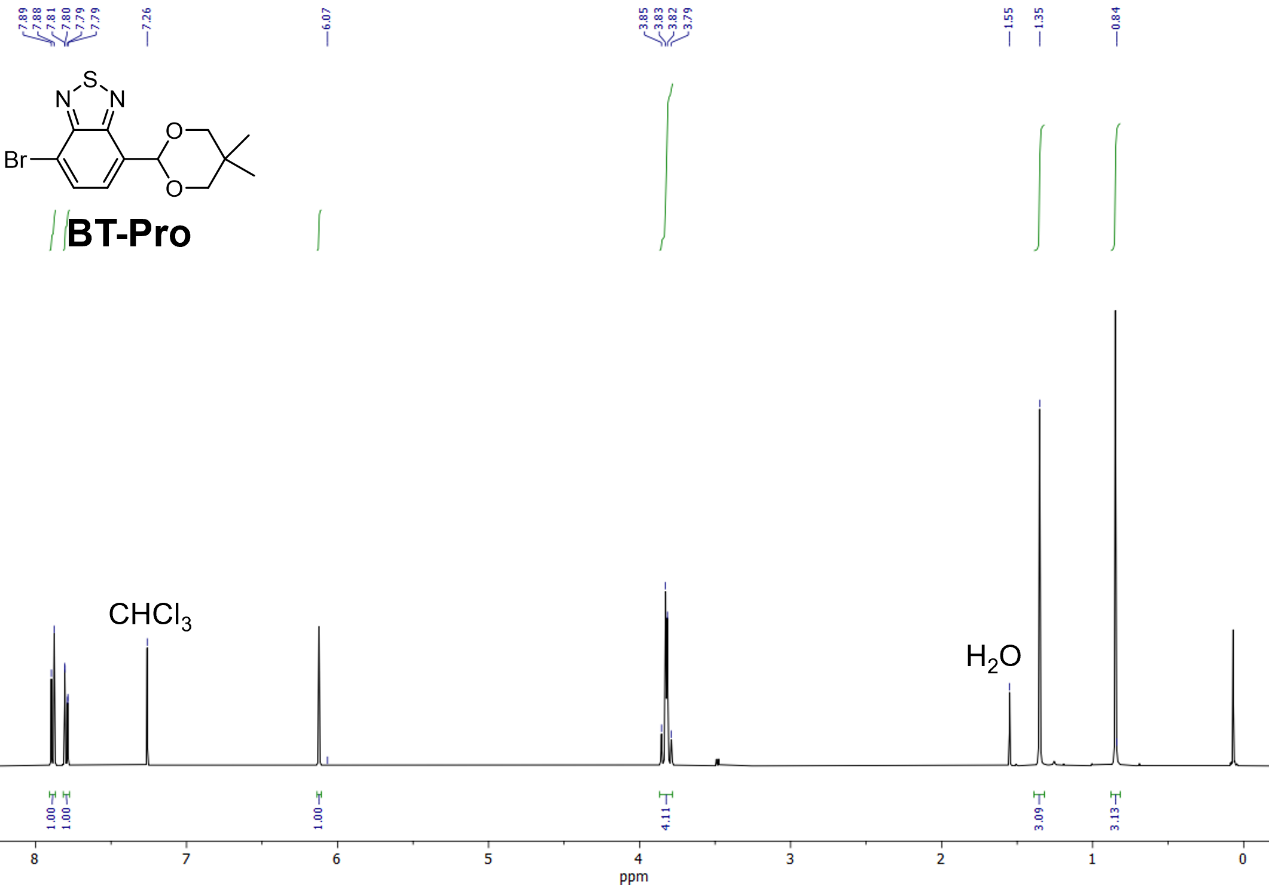
**

**(k)** ^1^H NMR spectra of **BT-Pro** in CDCl_3_.


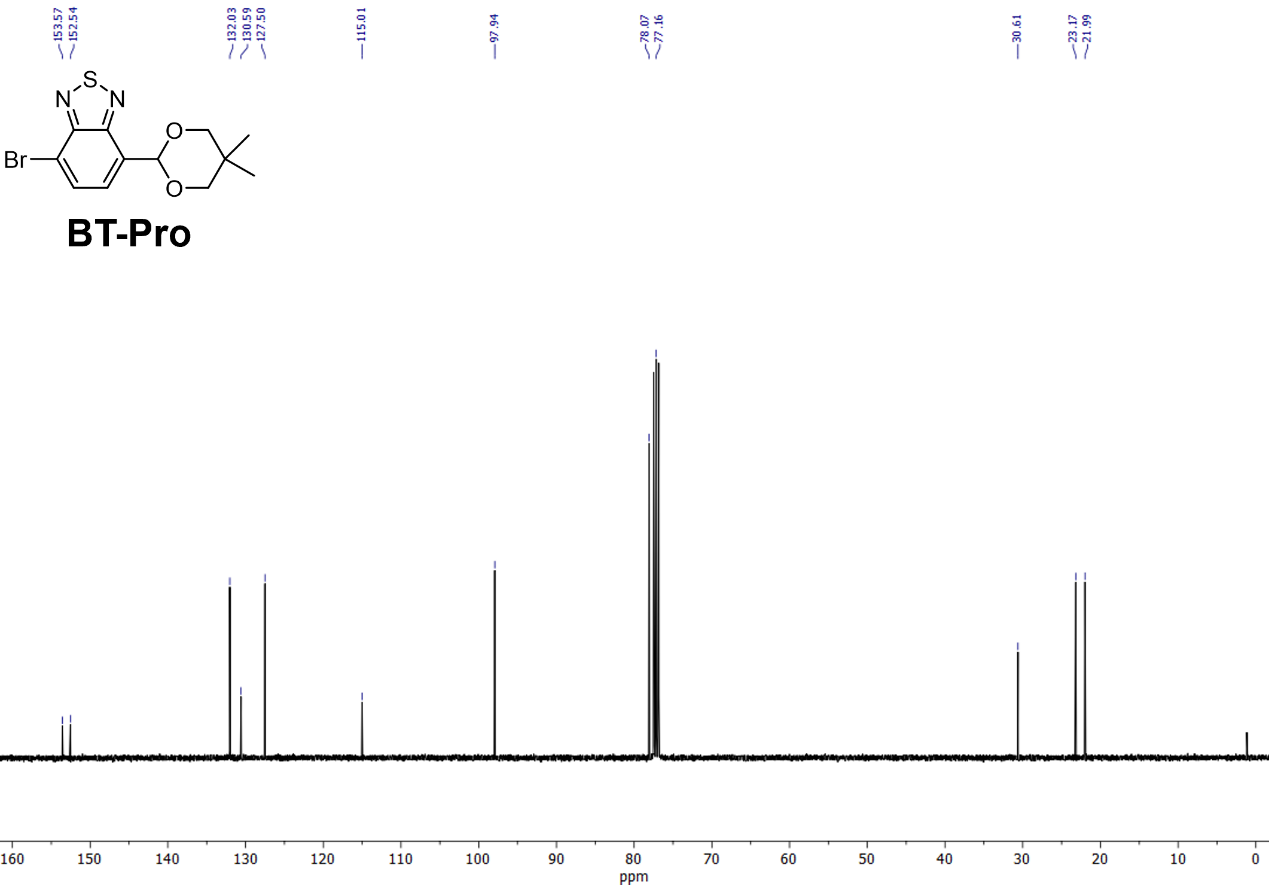


**(l)** ^13^C NMR spectra of **BT-Pro** in CDCl_3_.

**
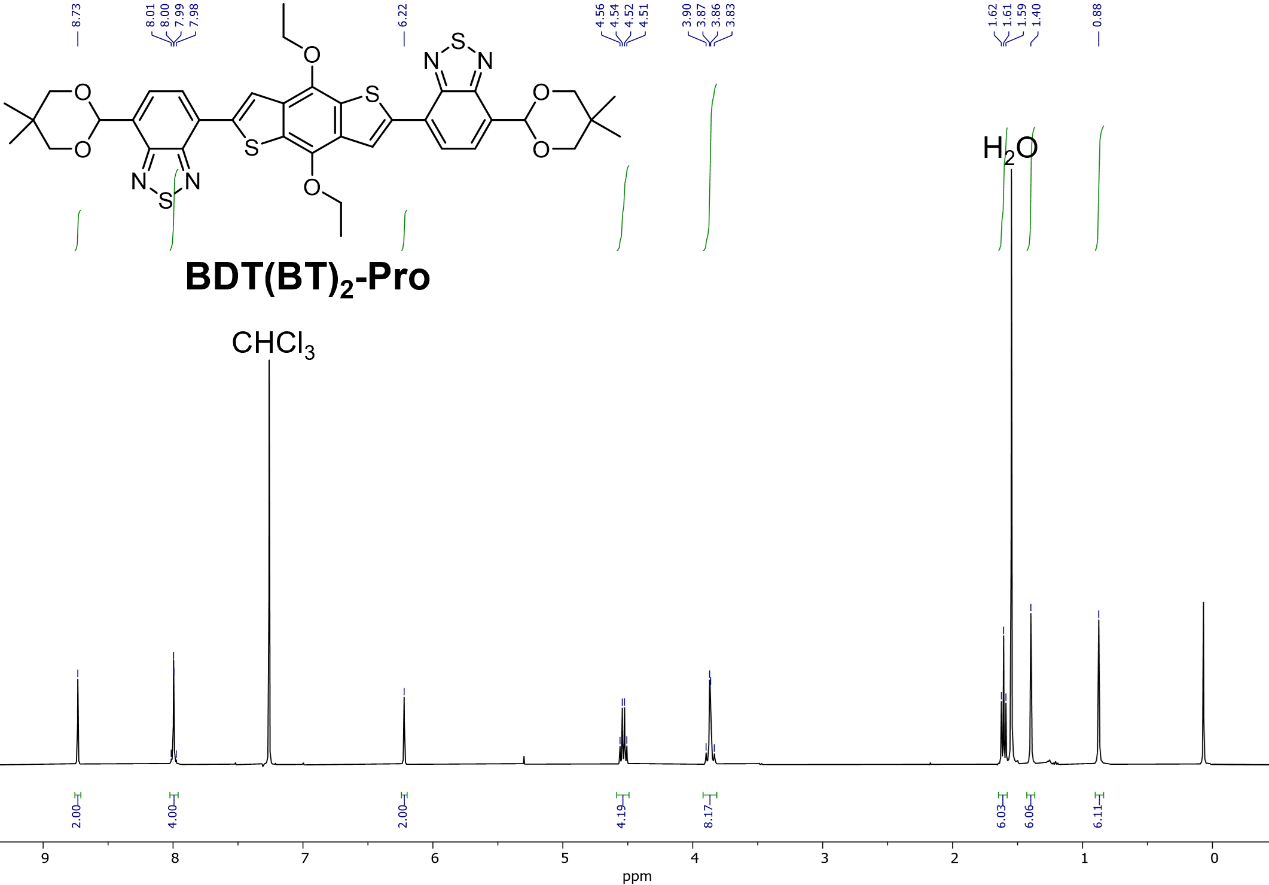
**

**(m)** ^1^H NMR spectra of **BDT(BT)_2_-Pro** in CDCl_3_.

**Figure S1**. NMR spectra of **TTE** (**a**), **TTE-4Br** (**b**, **c**), **TTN-4Br** (**d**), **TTNTA** (**e**, **f**), **BDT-OEt** (**g**, **h**), **BDT-OEt-2SnMe_3_** (**i**, **j**), **BT-Pro** (**k**, **l**) and **BDT(BT)_2_-Pro** (**m**).

3.2 Density Functional Theory (DFT) Calculations and Optical Properties of TTNTA


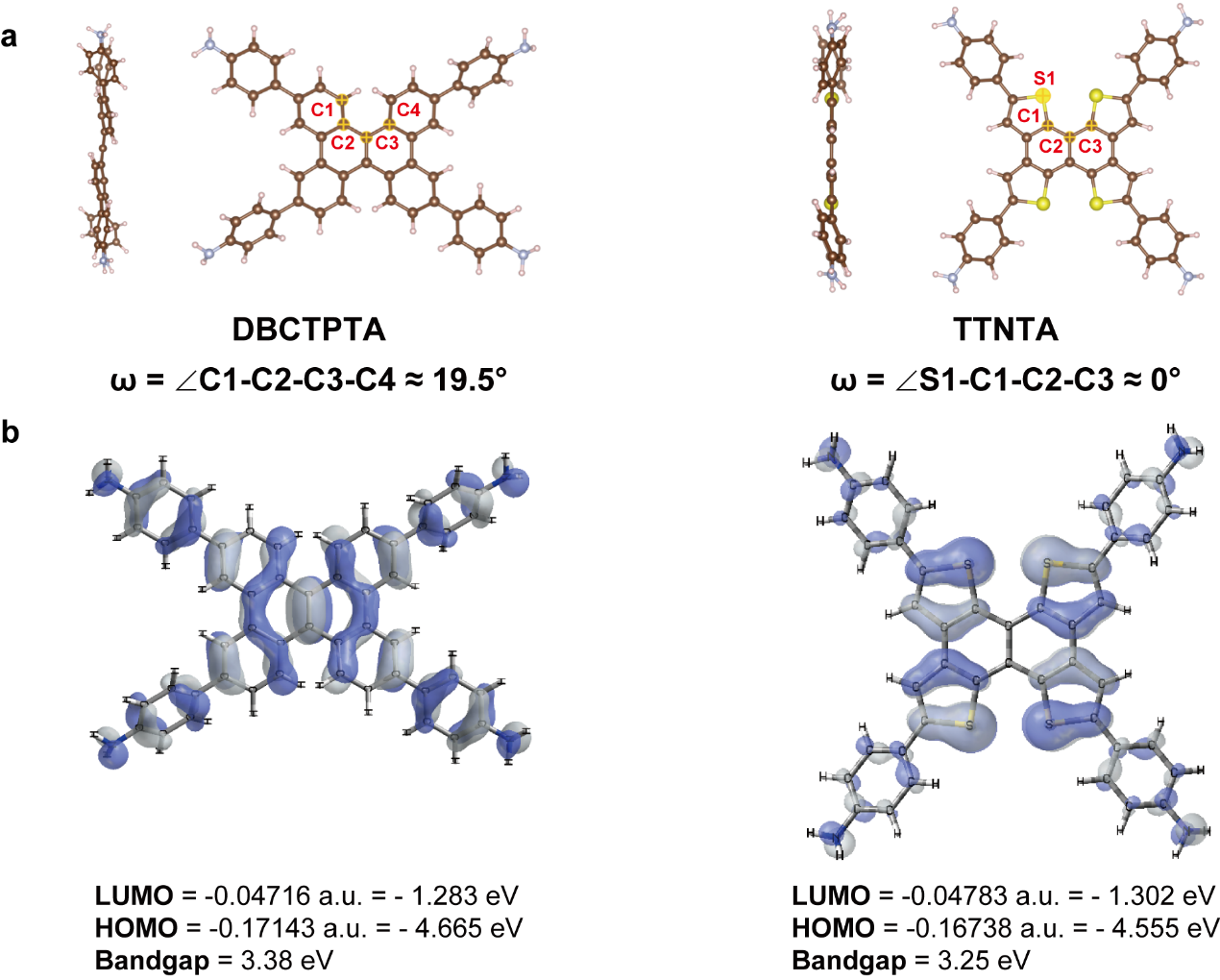


**Figure S2**. DFT calculated structures (**a**) and HOMO/LUMO energy levels (**b**) of the node **DBCTPTA** with a related structure reported in our previous work,^[7]^ and **TTNTA** in this work.

**
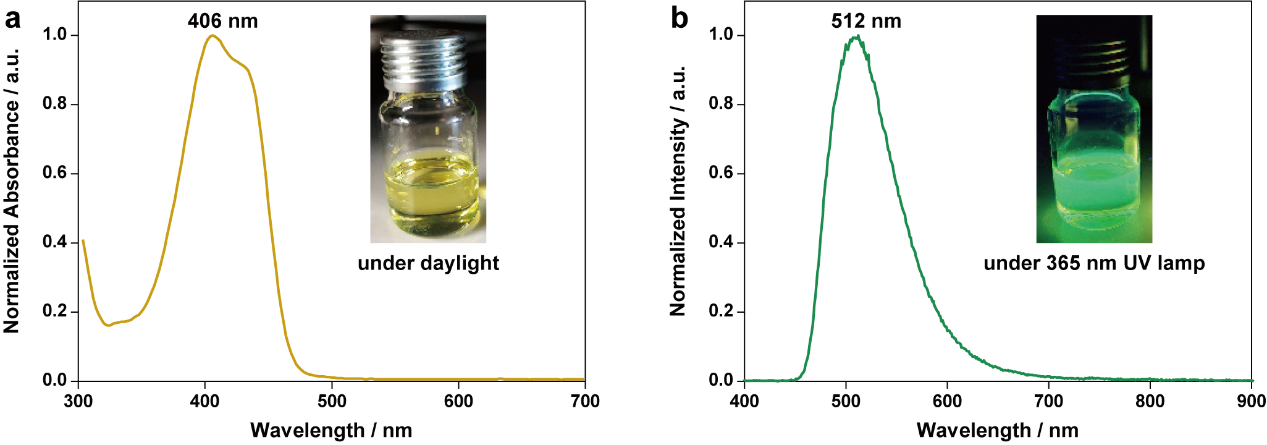
**

**Figure S3**. UV-Vis (**a**) and PL (**b**) spectra of 50 μM **TTNTA** in DMSO (the insets represent photographs of solutions under daylight and under a 365 nm UV lamp).

3.3 Cyclic Voltammograms (CV) of TTNTA

**
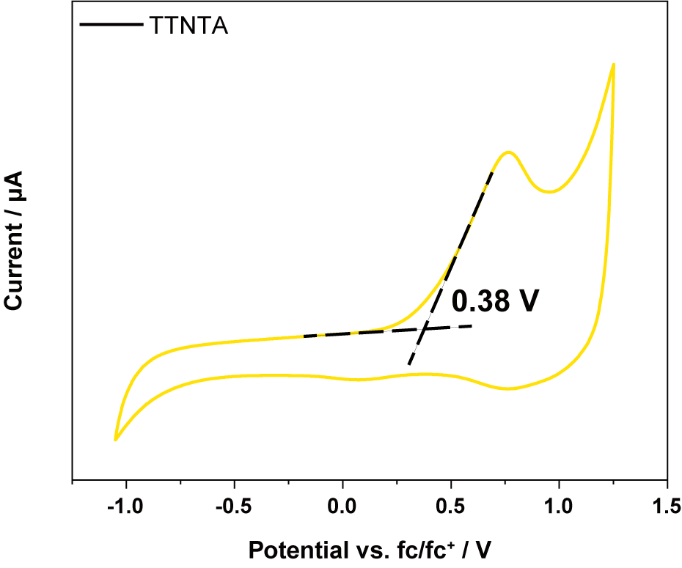
**

**Figure S4.** Cyclic voltammogram (CV) of **TTNTA** showing oxidation onset at 0.38 V against fc/fc^+^.

**
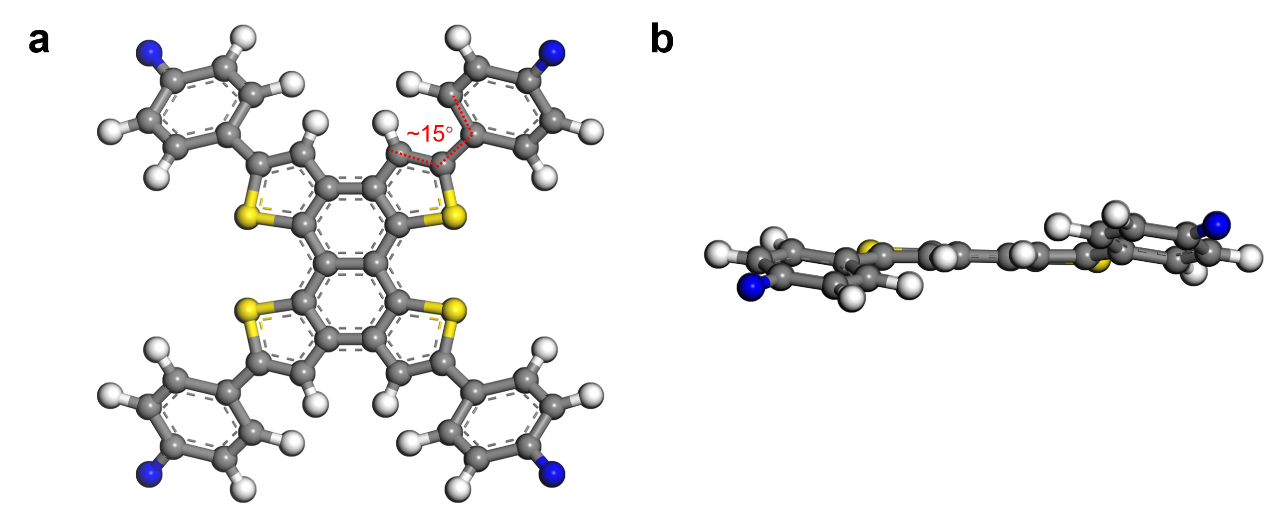
**

**Figure S5**. The conformation of the **TTNTA** node in **TT COF** in top view (**a**) and side view (**b**). The molecular model is based on the structure model of the **TT COF** using the Forcite module of the Accelrys Materials Studio software package. (gray (C), blue (N), yellow (S), white (H)).

3.4 FT-IR Spectra

**
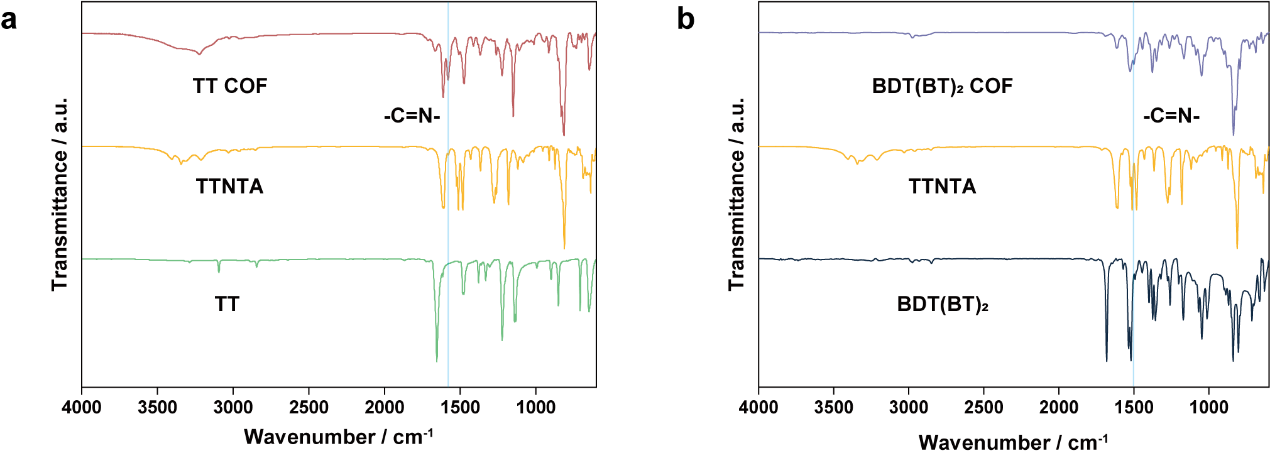
**

**Figure S6.** FT-IR spectra of building blocks and the respective COFs **TT COF** (**a**) and **BDT(BT)_2_ COF** (**b**). The blue lines mark newly appearing vibrational bands, indicating the formation of imine linkages.

3.5 Solid-state ^13^C Cross-Polarization Magic Angle Spinning (CP-MAS) NMR

**
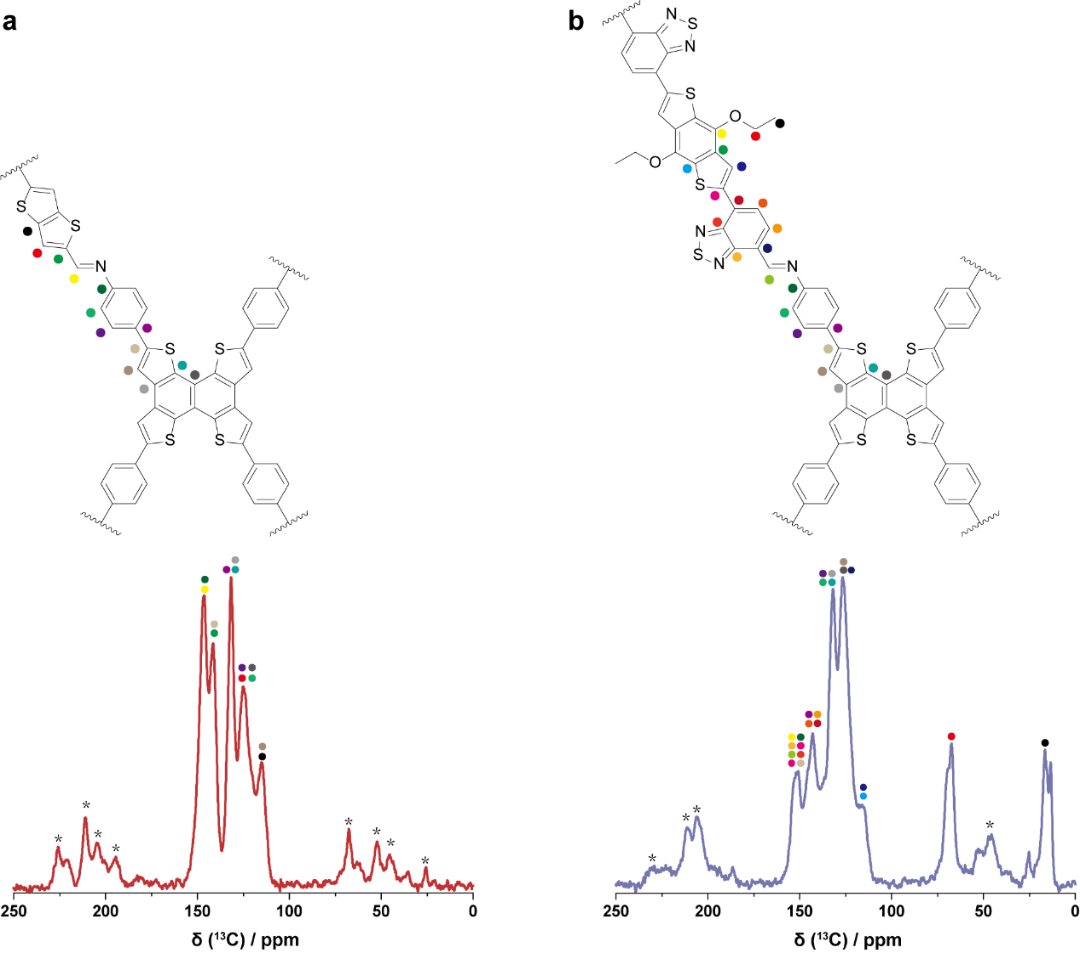
**

**Figure S7**. Solid-state ^13^C CP-MAS NMR spectra of **TT COF** (**a**) and **BDT(BT)_2_ COF** (**b**). The absence of the peak corresponding to the aldehyde functions at approximately 190 ppm as well as the presence of the peak at approximately 150 ppm corresponding to the imine bond confirms the formation of the imine-linked COFs. The signals marked with asterisks correspond to spinning sidebands. The peaks were assigned to the most probable carbon atoms.

3.6 Scanning Electron Microscopy (SEM) of COF Powders

**
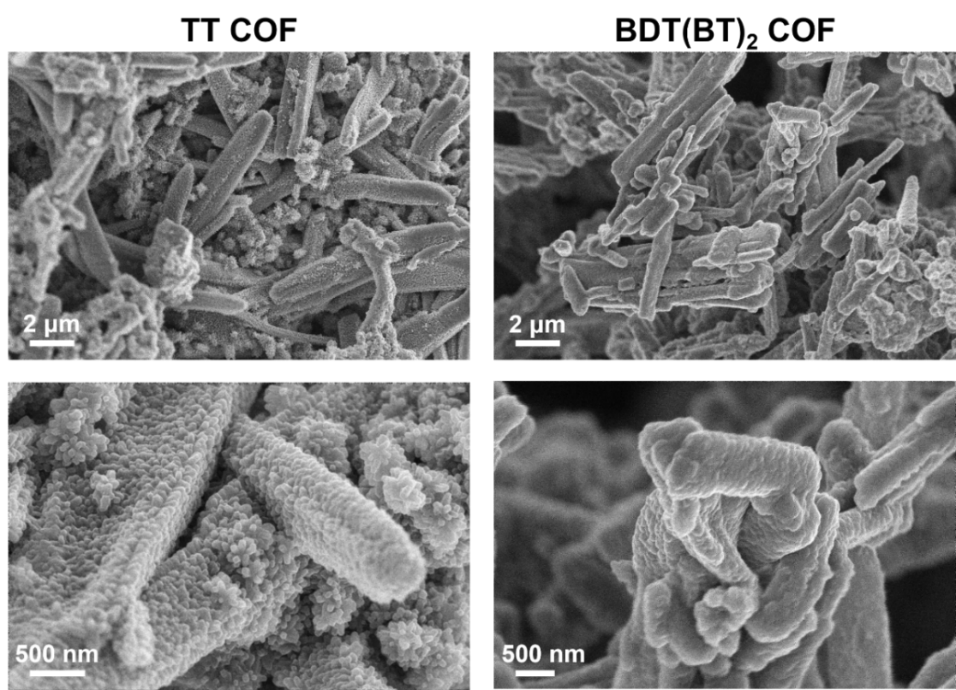
**

**Figure S8**. SEM images of **TT COF** (left) and **BDT(BT)_2_ COF** (right). Both COFs have typical well-defined crystallites forming large intergrown agglomerates.

3.7 High-Resolution Transmission Electron Microscopy (HRTEM) of COF Powders

**
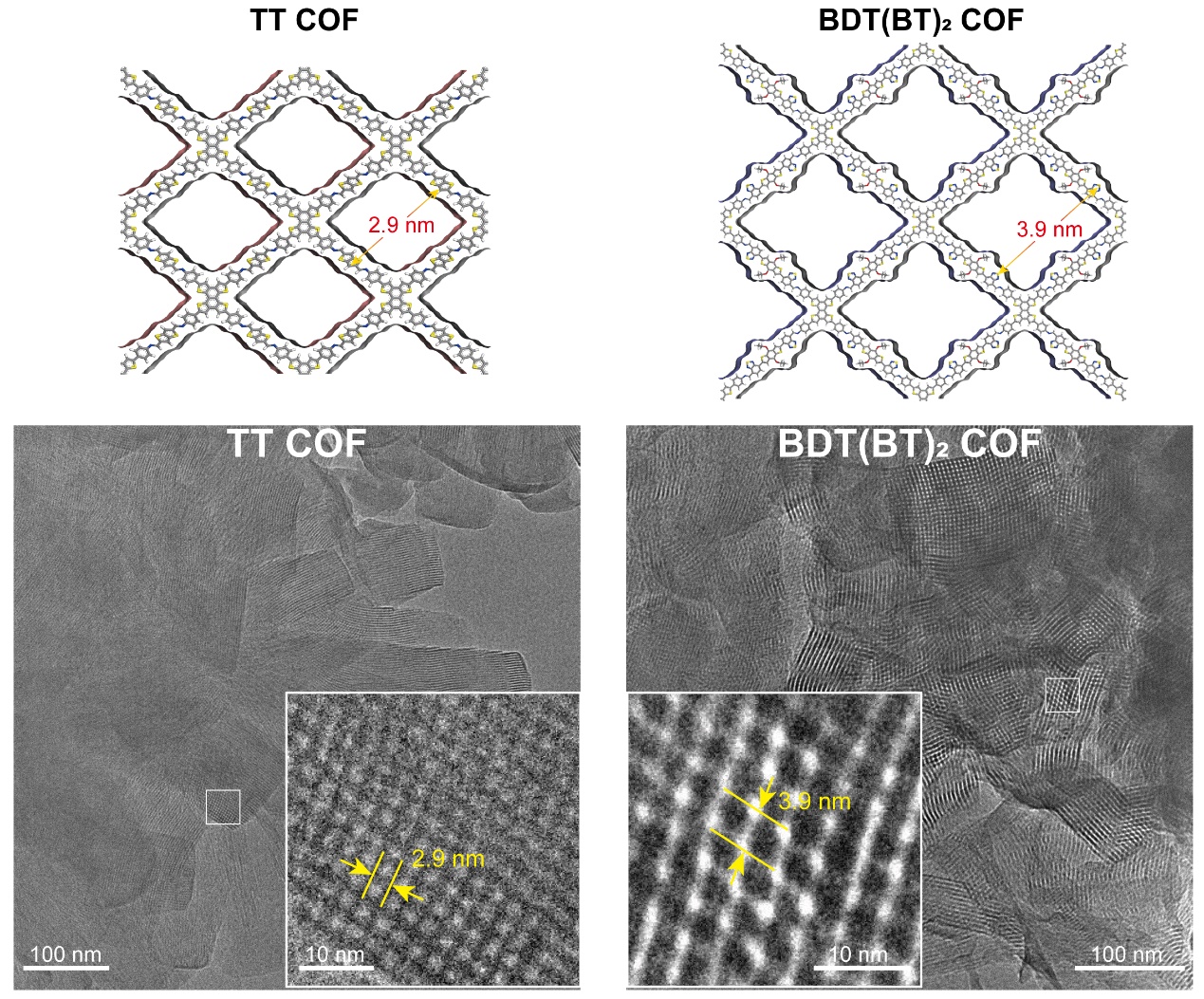
**

**Figure S9**. High resolution transmission electron microscope (HRTEM) images of **TT COF** (left) and **BDT(BT)_2_ COF** (right).

3.8 Solvent Stability Evaluation of COF Powders

**
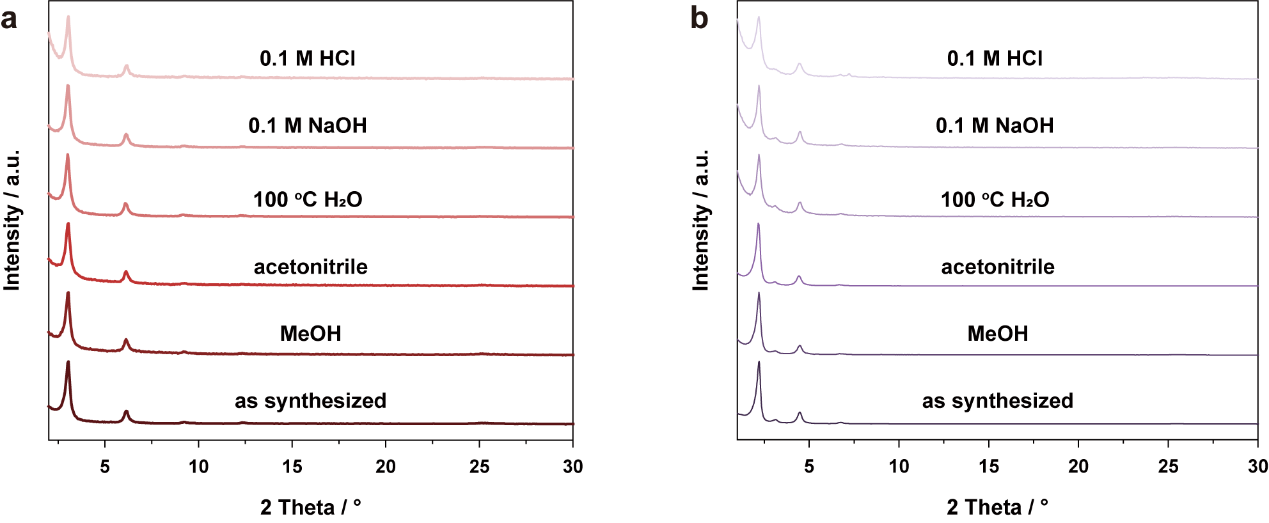
**

**Figure S10**. PXRD patterns of **TT** (**a**) and **BDT(BT)_2_** (**b**) **COF** after 12 hours of exposure to various solvent conditions. If no temperature is noted, the experiments were performed at room temperature. The samples were vacuum-dried after solvent exposure prior to measurement.

3.9 Thermogravimetric Analysis


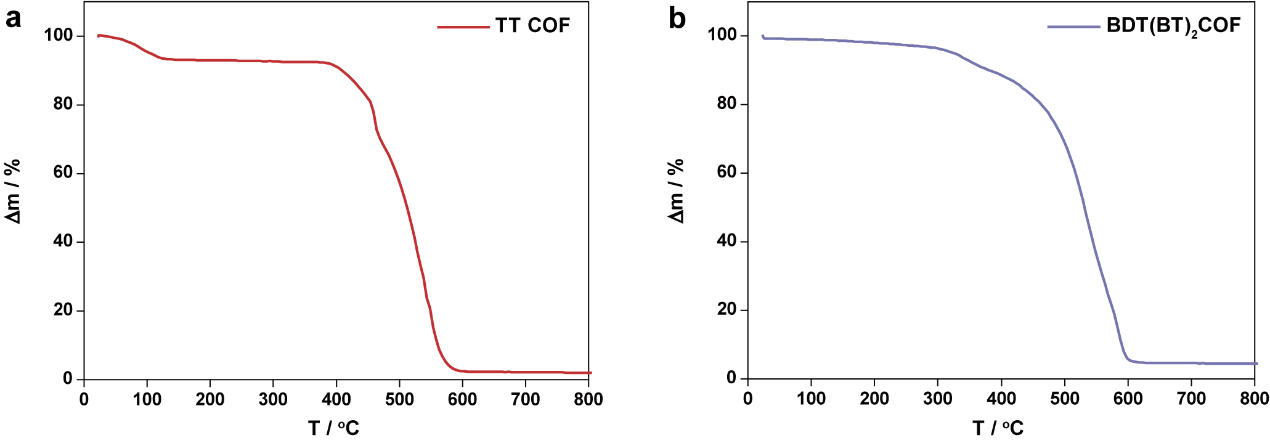


**Figure S11**. The thermogravimetric analysis (TGA) analysis of **TT** (**a**) and **BDT(BT)_2_** (**b**) **COF** measured as bulk materials.

3.10 Grazing-Incidence Wide-Angle X-ray Scattering (GIWAXS) and SEM of COF Films


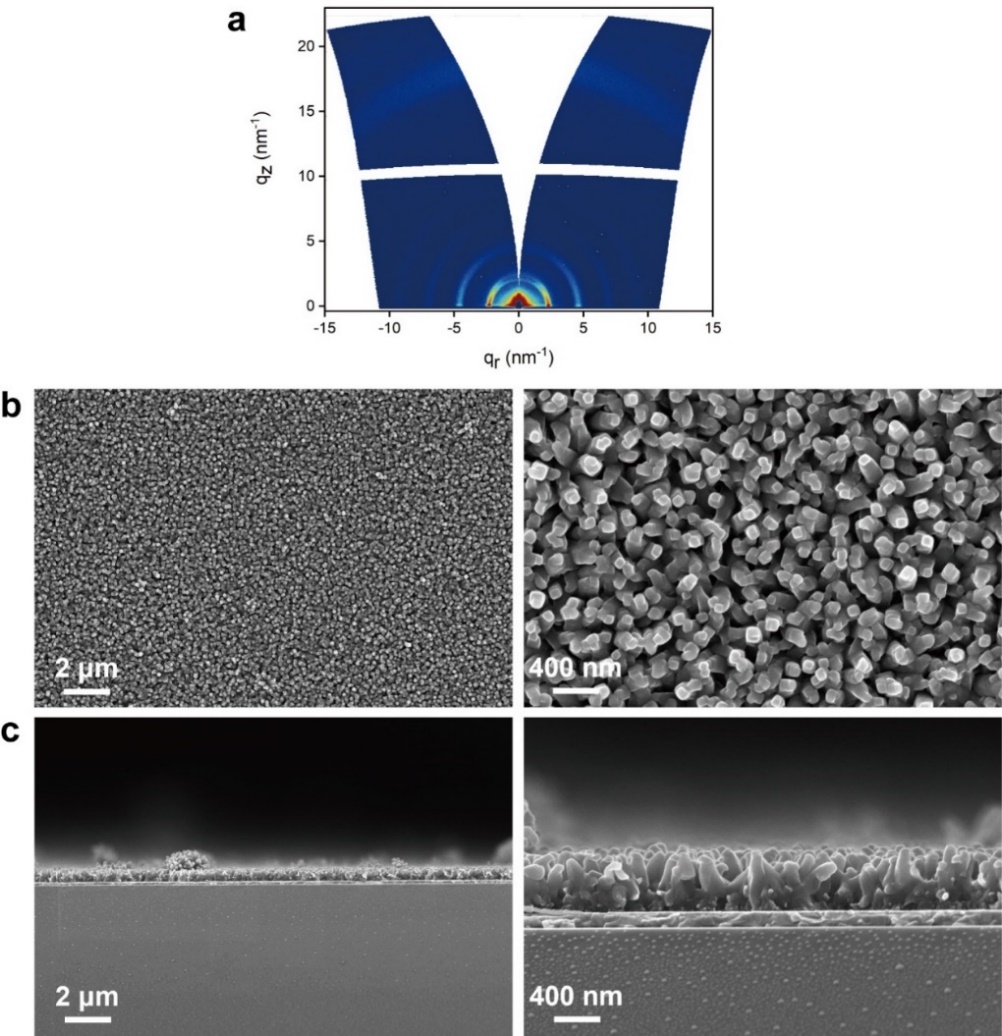


**Figure S12**. (**a**) GIWAXS 2D pattern of a **TT COF** thin film grown on an ITO-coated glass substrate by using the solvothermal synthesis method. SEM top-view (**b)** and cross section (**c**) images of **TT COF** thin film at different magnifications, respectively.


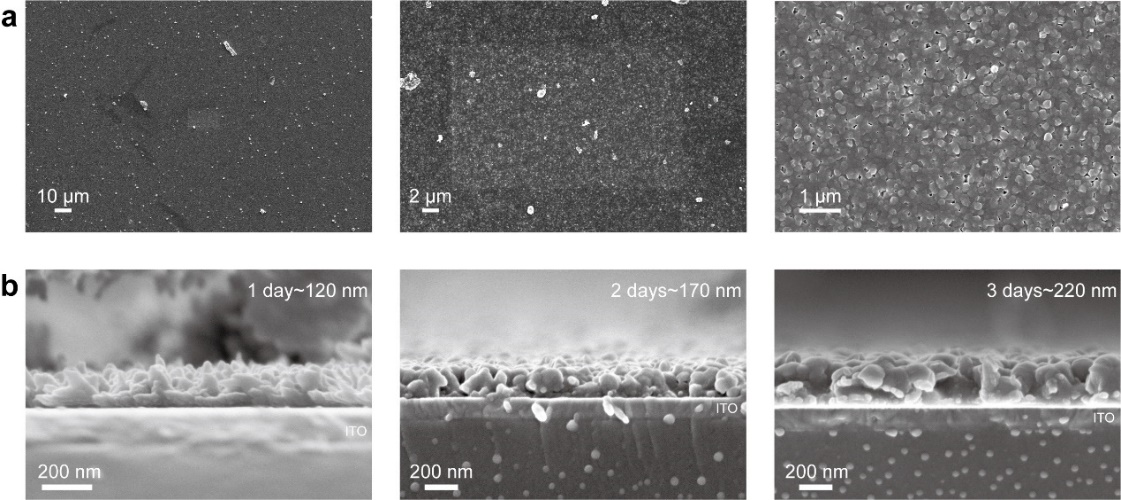


**Figure S13**. (**a**) SEM top-view images, at different magnifications, of **BDT(BT)_2_ COF** thin film after growing for 3 days on ITO-coated glass substrate. (**b**) SEM cross-section images of thin films of **BDT(BT)_2_ COF** grown by the solvothermal procedure on ITO-coated glass substrates, with different film synthesis times from 1 to 3 days.


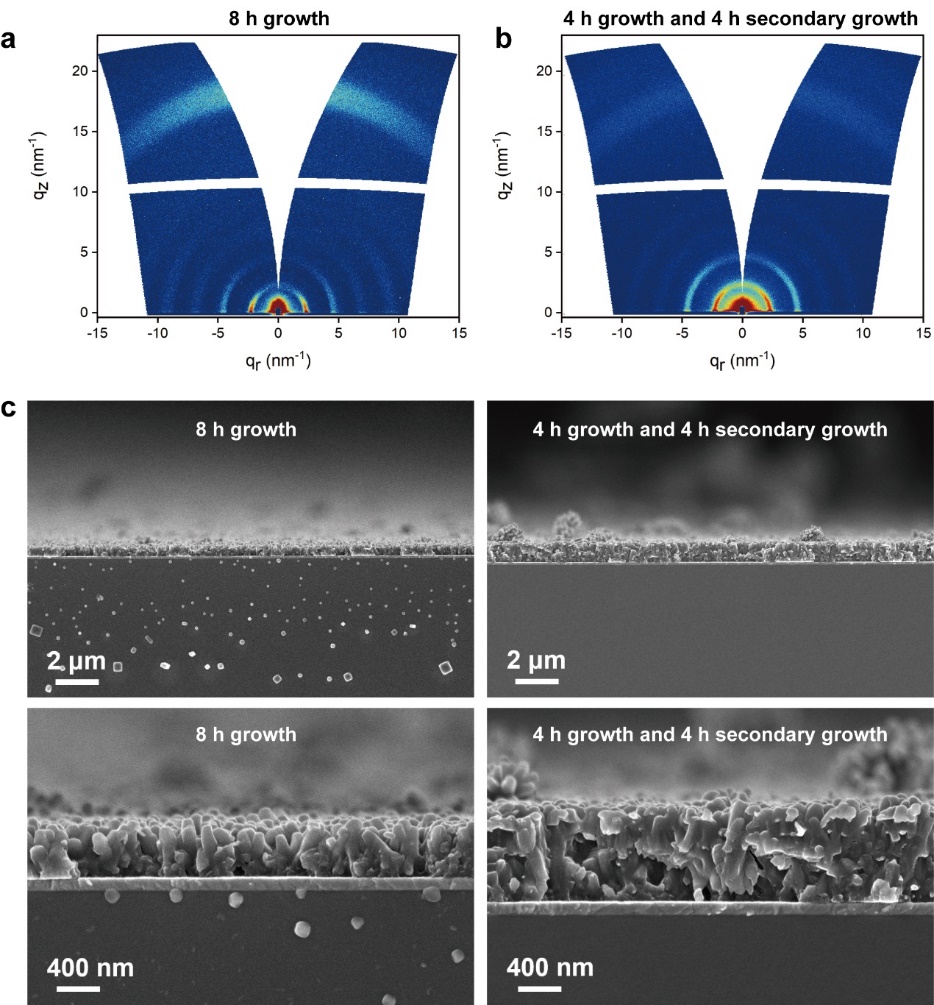


**Figure S14**. GIWAXS 2D patterns of **TT COF** thin films grown on an ITO-coated glass substrate after 8 h synthesis (**a**) and by using a secondary growth method (4 h growth followed by 4 h secondary growth) (**b**). (**c**) SEM cross section images at different magnifications of TT COF thin films grown according to the two methods shown in (a). For the secondary growth, the film was cleaned and dried as described in the film synthesis section after the first 4 h synthesis, after which the resulting film was placed into a freshly prepared precursor solution for the secondary growth. The thickness of the film could be increased to around 800 nm after secondary growth with a more tightly covered COF layer. Importantly, the secondary growth method did not significantly affect the preferred orientation of the COF as shown in the GIWAXS 2D pattern.

3.11 HRTEM of TT and BDT(BT)_2_ COF Films


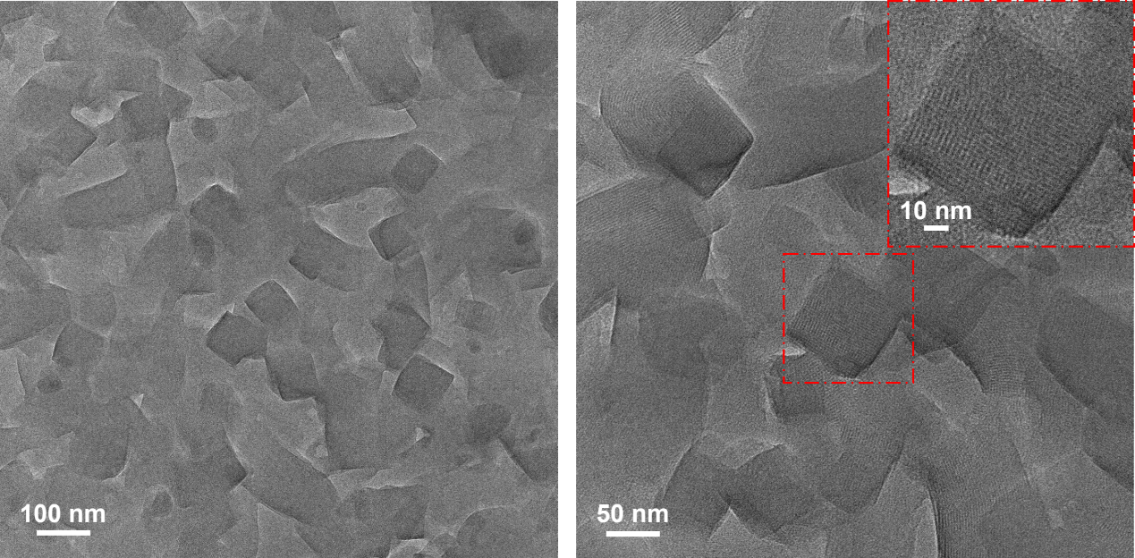


**Figure S15**. HRTEM images at different magnifications of a **TT COF** film removed from an ITO-coated glass substrate.


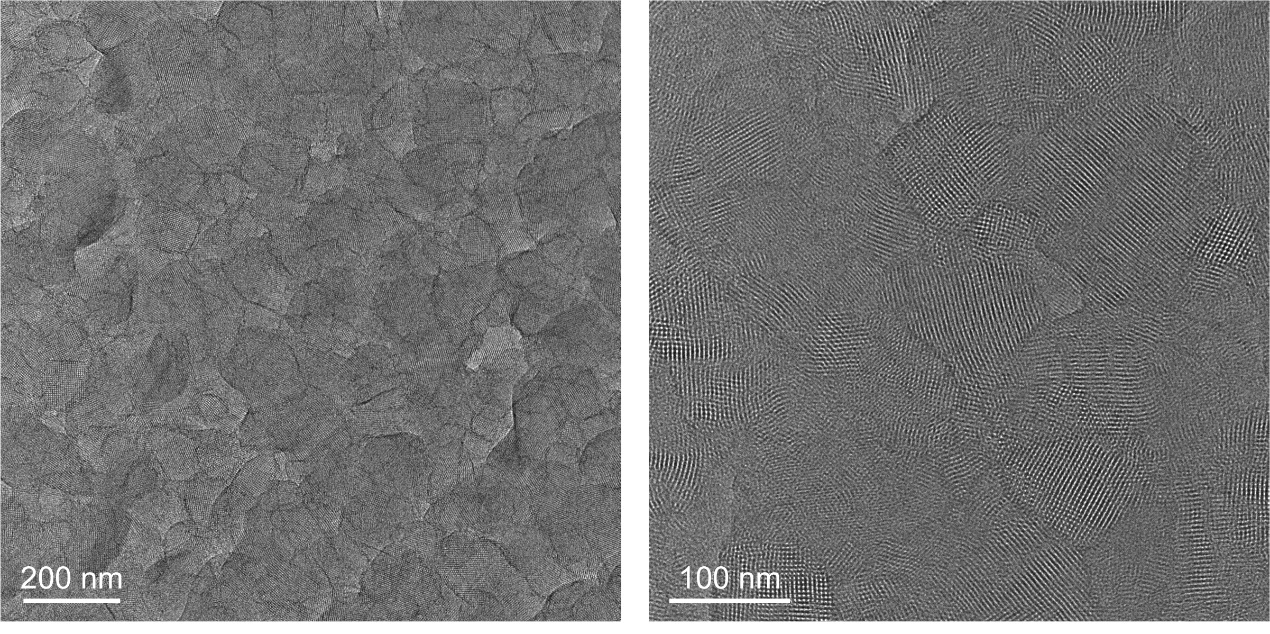


**Figure S16**. HRTEM images at different magnifications of a **BDT(BT)_2_ COF** film removed from an ITO-coated glass substrate.

3.12 Optical Properties of COF Films Characterized by UV-Vis and PL Spectroscopy


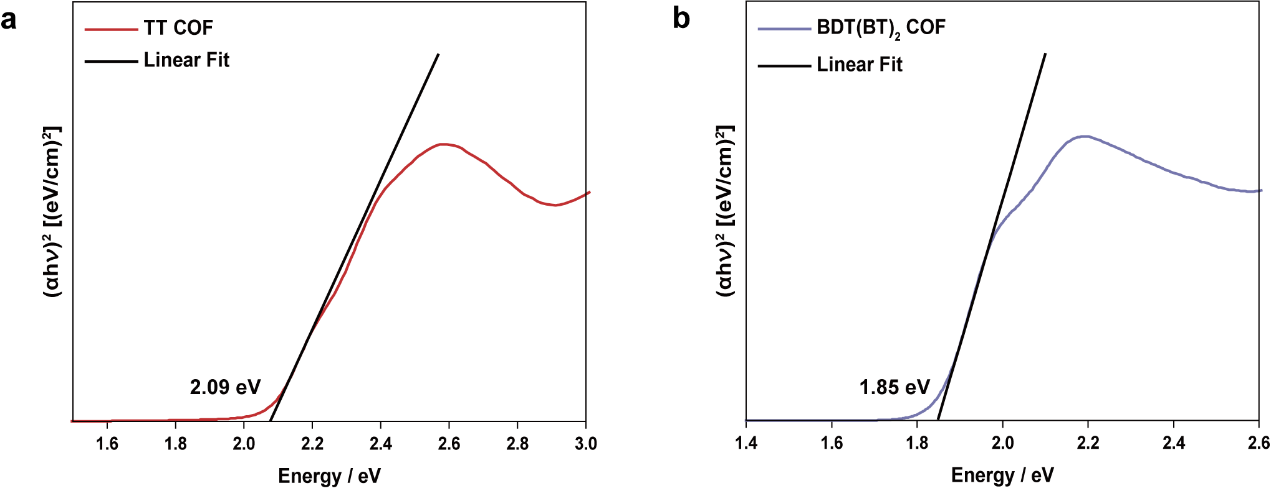


**Figure S17**. Tauc plots of the UV-Vis absorbance spectra calculated for a direct band gap transition model for **TT** (**a**) and **BDT(BT)_2_ COF** (**b**), respectively.


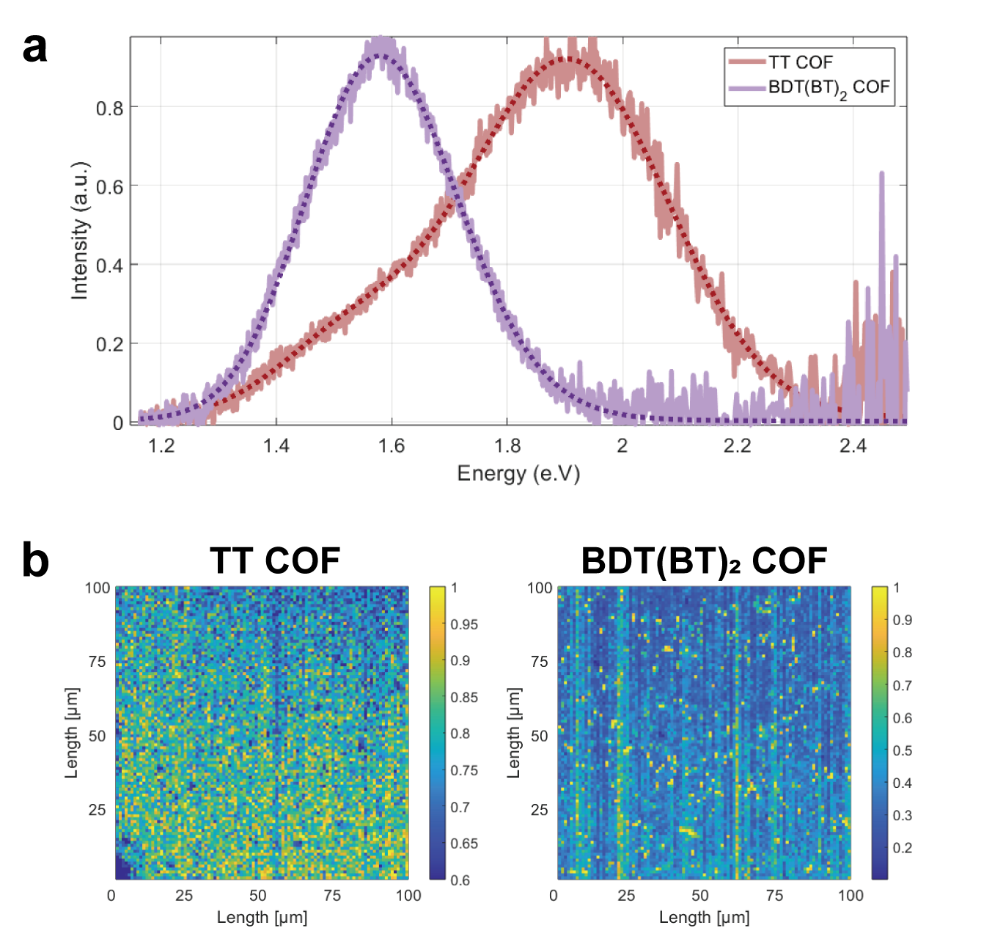


**Figure S18**. (**a**) Photoluminescence spectra of the **TT** and **BDT(BT)_2_ COF** films measured with 476 nm excitation. Both films were prepared on glass substrates *via* solvothermal procedure. (**b**) PL mapping of **TT** and **BDT(BT)_2_ COF** films measured on an area of 100 μm × 100 μm on glass. Both COF films were measured with 476 nm excitation. The observed rather uniform PL intensity distributions in both films with 476 nm excitation confirm the good homogeneity of both COF films.


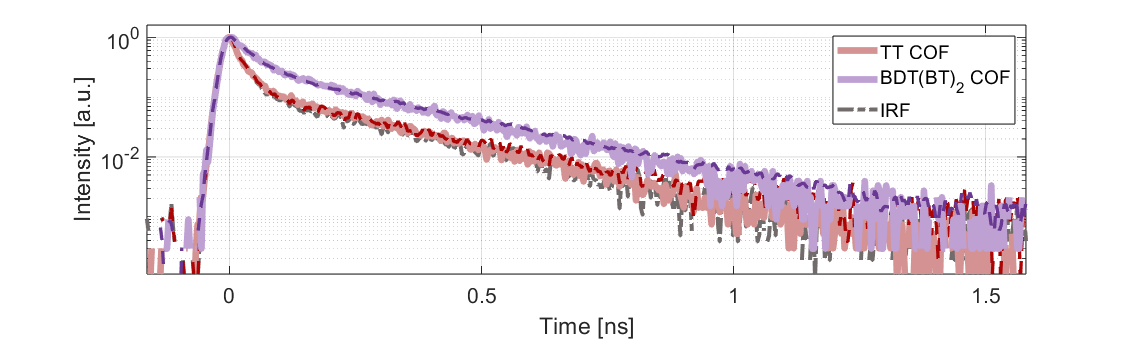


**Figure S19**. PL transients at room temperature for **TT COF** film (red line), BDT(BT)_2_ COF film (purple line), and the instrument response function (IRF) of the setup (black line). The transients are fitted using the stretched-exponential model function convoluted with the IRF, shown as the corresponding dashed lines. The **BDT(BT)_2_ COF** film exhibits a much longer average lifetime than the **TT COF** film. The average lifetimes of << 9 ps for **TT COF** film and 9 ps for **BDT(BT)_2_ COF** film were obtained from a stretched-exponential fit.

3.13 In-Plane Electrical Conductivity Measurement of TT COF Film


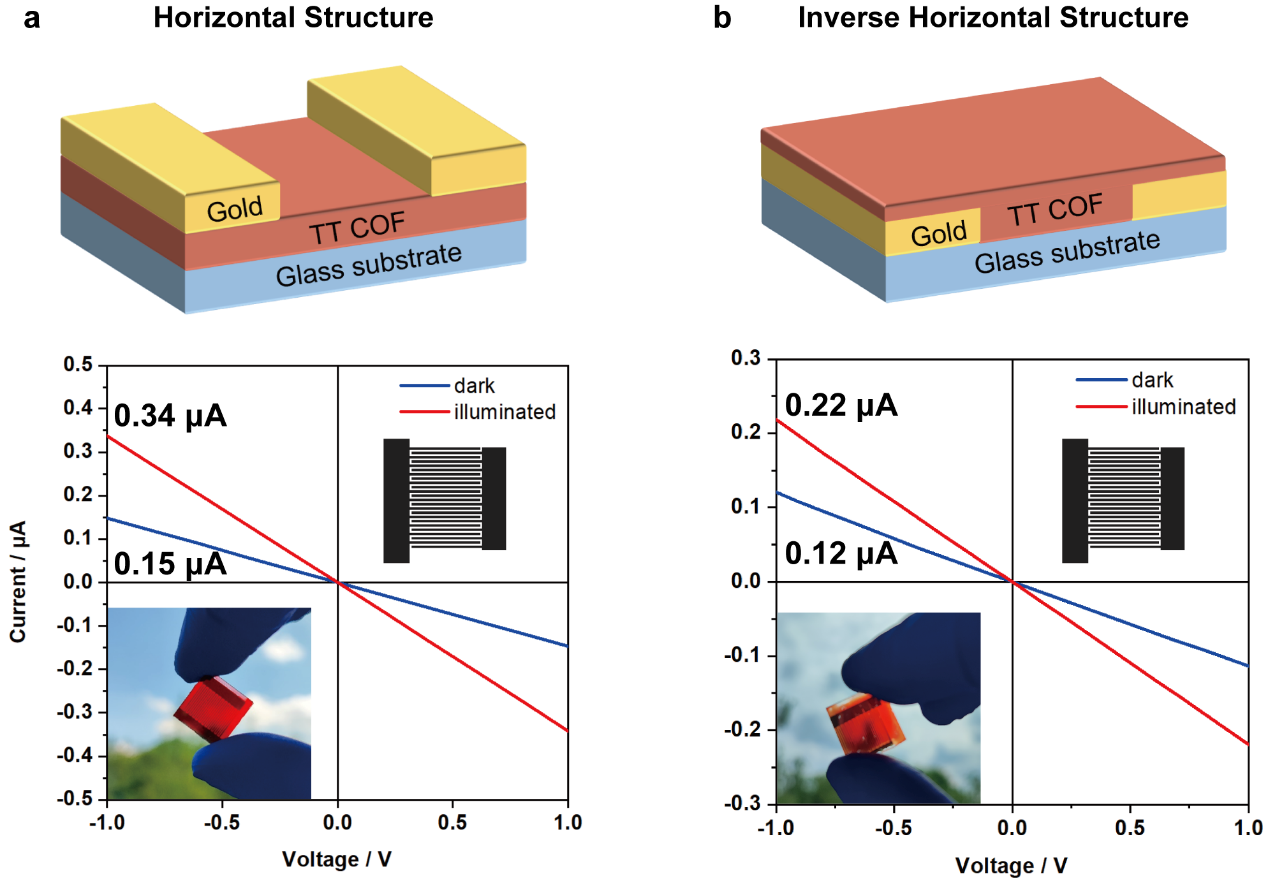


**Figure S20**. (**a**) Current-voltage (*I-V*) characteristics in the voltage range between -1 and 1 V of the **TT COF** thin film measured in-plane under dark conditions (blue trace) and under AM 1.5 G illumination (red trace), using gold electrodes thermally evaporated directly onto the COF film. The corresponding device structure is illustrated above (left). (**b**) *I-V* characteristics in the voltage range between -1 and 1 V of the **TT COF** film measured in-plane, where the COF was synthesized directly on top of pre-patterned gold electrodes evaporated onto the substrate. The corresponding device structure is shown top-right. In both electrode configurations, the channel length and width were maintained at 200 mm × 0.25 mm, respectively. The light/dark ratios were calculated from the I-V curve slopes.

| **Device** | **I_max_ / μA** | **slope** | **light/dark ratio** |
| --- | --- | --- | --- |
| Horizontal Structure | 0.34 (light) | -3.42×10^-1^ | 2.34 |
|  | 0.15 (dark) | -1.46×10^-1^ |  |
| Inverse Horizontal Structure | 0.22 (light) | -2.17×10^-1^ | 1.85 |
|  | 0.12 (dark) | -1.17×10^-1^ |  |

**Table S1**. Maximum electrical current obtained at a -1 V applied bias under dark conditions and under 1 sun illumination, along with the light/dark current ratio calculated from the slopes of the I–V curves.

3.14 Extraction of charge-carrier mobility from OPTP measurements

The effective charge-carrier mobility values reported for COF thin films were extracted here from fluence-dependent OPTP measurements, following the method developed by Wehrenfennig et al.^[8]^ By assuming semiconductor thin film materials with thicknesses smaller than the wavelength of the incident THz radiation, we estimated the sheet photoconductivity from the fractional change in the transmitted THz electric field ${\Delta T}/T$ as

| $\Delta S= -\epsilon_{0}c(n_{1}+n_{3})\left( \frac{\Delta T}{T} \right)$ | (Eq. S1) |
| --- | --- |

where *n_1_* = 2.13 and *n_3_* = 1 are the refractive indexes of quartz and vacuum,^[9]^ respectively.

To extract the effective charge-carrier mobility from the sheet photoconductivity, we estimated the number of initially photogenerated carriers as

| $N= \varphi\frac{E\lambda}{hc}\left( 1-R_{pump}-T_{pump} \right)$ | (Eq. S2) |
| --- | --- |

Where $\varphi$ is the photon-to-charge branching ratio (i.e., the fraction of generated charges per photon absorbed), *E* is the pump pulse energy, $\lambda$ is the excitation wavelength, and *R_pump_* and *T_pump_* are the reflectance and transmittance of the sample at the excitation wavelength (400 nm). The charge-carrier mobility *μ* can be calculated as

| $\mu=\frac{\Delta S A_{eff}}{Ne}$ | (Eq. S3) |
| --- | --- |

Where *A_eff_* is the effective overlap area between THz and pump beam and *e* is the elementary charge.^[8]^ By combining Equation S1 and Equation S2 into Equation S3, we extract the effective charge-carrier mobility value as:

| $\varphi\mu= -\epsilon_{0}c(n_{1}+n_{3})\frac{A_{eff}hc}{eE\lambda(1-R_{pump}-T_{pump})}\left( \frac{\Delta T}{T} \right)$ | (Eq. S4) |
| --- | --- |

Crucially, the sheet photoconductivity signal measured here is contributed by both photogenerated free electrons and holes. Therefore, the extracted charge-carrier mobility is the effective electron-hole sum mobility.

To capture the photoconductivity dynamics in COFs, we fitted the OPTP traces with a biexponential decay function convoluted with a Gaussian instrumental response function ($IRF \approx200 fs$).^[10]^ The OPTP signal is then expressed as

| $\left( \frac{\Delta T}{T} \right)\left( t \right)=\left[ \alpha_{1}\exp\left( {-k}_{exc}t \right)+\alpha_{2}\exp\left( {-k}_{decay}t \right) \right] \bigotimes g(t,t_{0},\sigma)$ | (Eq. S5) |
| --- | --- |

Where *k_exc_* is the exciton formation rate, *k_decay_* is the subsequent remnant charge-carrier recombination rate, with $\alpha$_1_ and $\alpha$_2_ being the respective fractional amplitudes. $g(t,0,\sigma)$ is a Gaussian function centered at *t_0_* with broadening *σ*. The resulting fits are reported in Figure 3a and Figure S20 and fitting parameters are reported in Table S2.

| **Sample** | $\alpha$**_1_** | *k_exc_* [ps^-1^] | $\alpha$**_2_** | *k_decay_* [ps^-1^] |
| --- | --- | --- | --- | --- |
| BDT(BT)_2_ COF | 0.91 | 1.9 | 0.09 | 0.008 |
| TT COF | 0.89 | 2.4 | 0.11 | 0.075 |

**Table S2**. Biexponential fitting parameters for photoconductivity transients measured for COF thin films following 3.1-eV pulsed excitation.

**Drude Factor.** The Drude model is a simple classical model describing the frequency-dependent conductivity in semiconductors and metals. For photogenerated electrons and holes in an emerging semiconductor, generally exhibiting charge-carrier scattering times at the order of a few femtoseconds,^[11]^ the Drude model predicts zero-valued imaginary and frequency-independent real components of the photoconductivity in the THz range investigated in this work (0.5-2.5 THz).^[12, 13]^ Milot et al. introduced a *Drude factor* $f_{D}$ to quantify deviations from it, defined as:^[14]^

| $f_{D}=\frac{1}{n} \sum_{n} \frac{\sqrt{Re\left( {\Delta T}/T \right)^{2}}}{\sqrt{Re\left( {\Delta T}/T \right)^{2}+Im\left( {\Delta T}/T \right)^{2}}}$ | (S5) |
| --- | --- |

Where *n* is the number of frequency points measured in the THz spectrum, and ${\Delta T}/T$ is the measured fractional change in THz transmission at each frequency point. The Drude factor values can range between 0 and 1, where 1 indicates the ideal Drude conductivity model, while deviations from it can originate from several effects ranging from localization to disorder-induced dispersive transport, as well as exciton formation and phonon resonances.


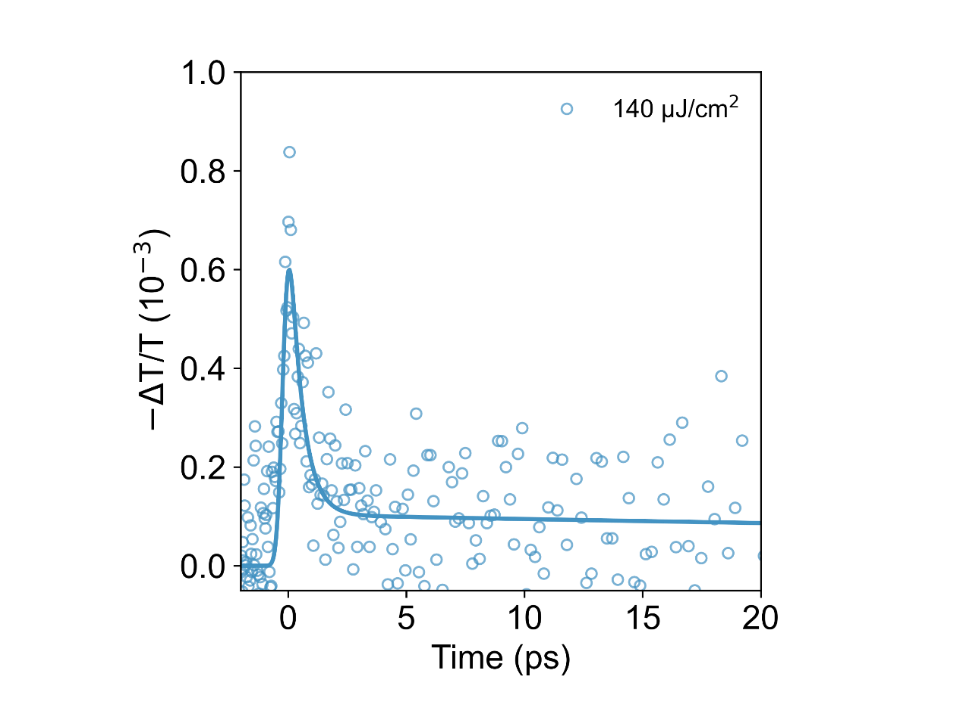


**Figure S21**. Fluence-dependent OPTP dynamics in **BDT(BT)_2_ COF** film following an excitation photon energy of 3.10 eV, with a pump fluence of 140 μJ cm^-2^. Blue circles represent experimental data, solid blue line represents the fit to a biexponential model.

3.15 CV Analysis of COF Films


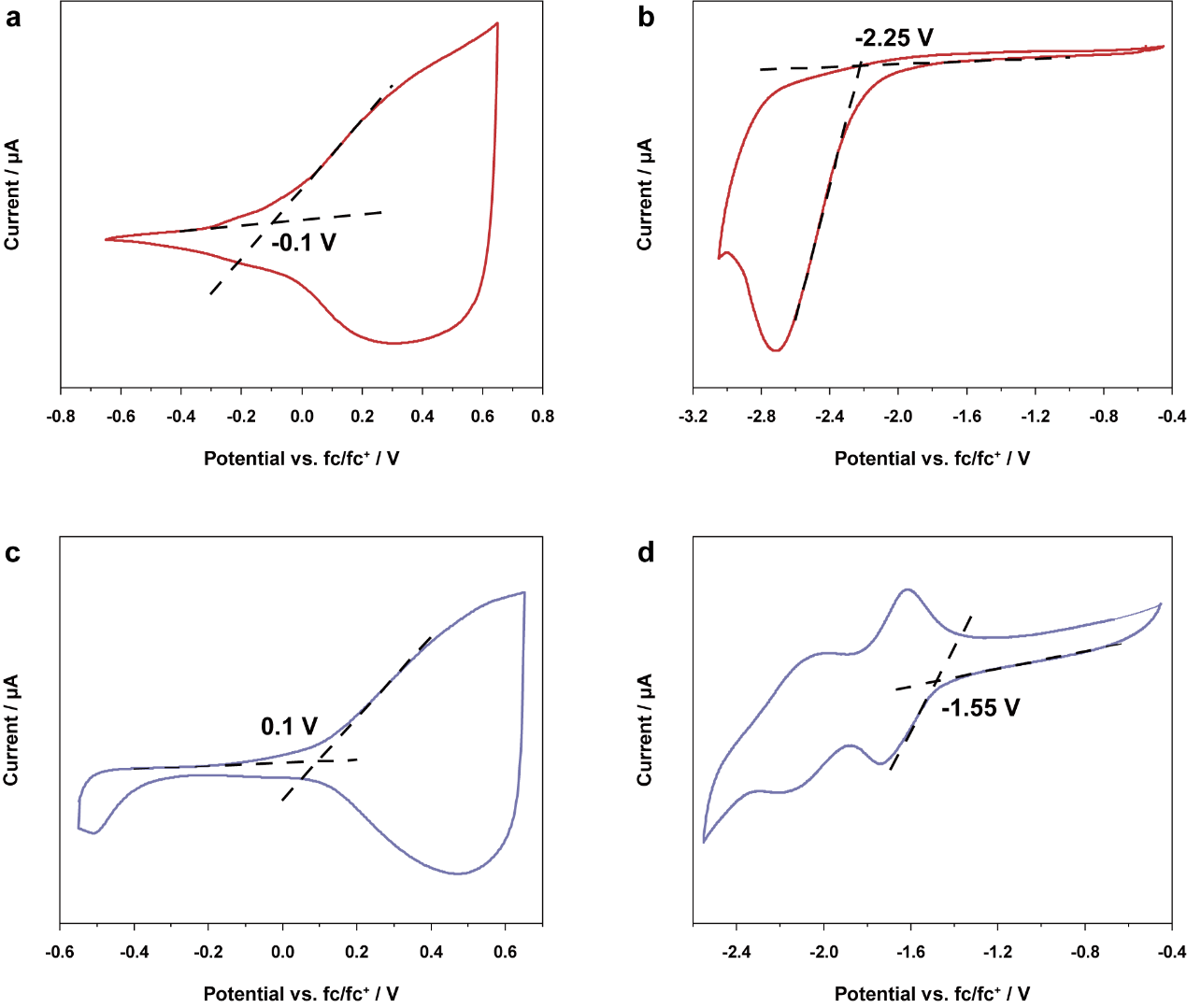


**Figure S22**. Cyclic voltammograms of (**a**, **b**) **TT** and (**c**, **d**) **BDT(BT)_2_ COF** films with 0.1 M tetrabutylammonium hexafluorophosphate in anhydrous acetonitrile as electrolyte. The potential was calibrated against the fc/fc^+^ redox couple (−5.10 eV versus vacuum level).

3.16 GIWAXS Measurement of COF Films After Illumination


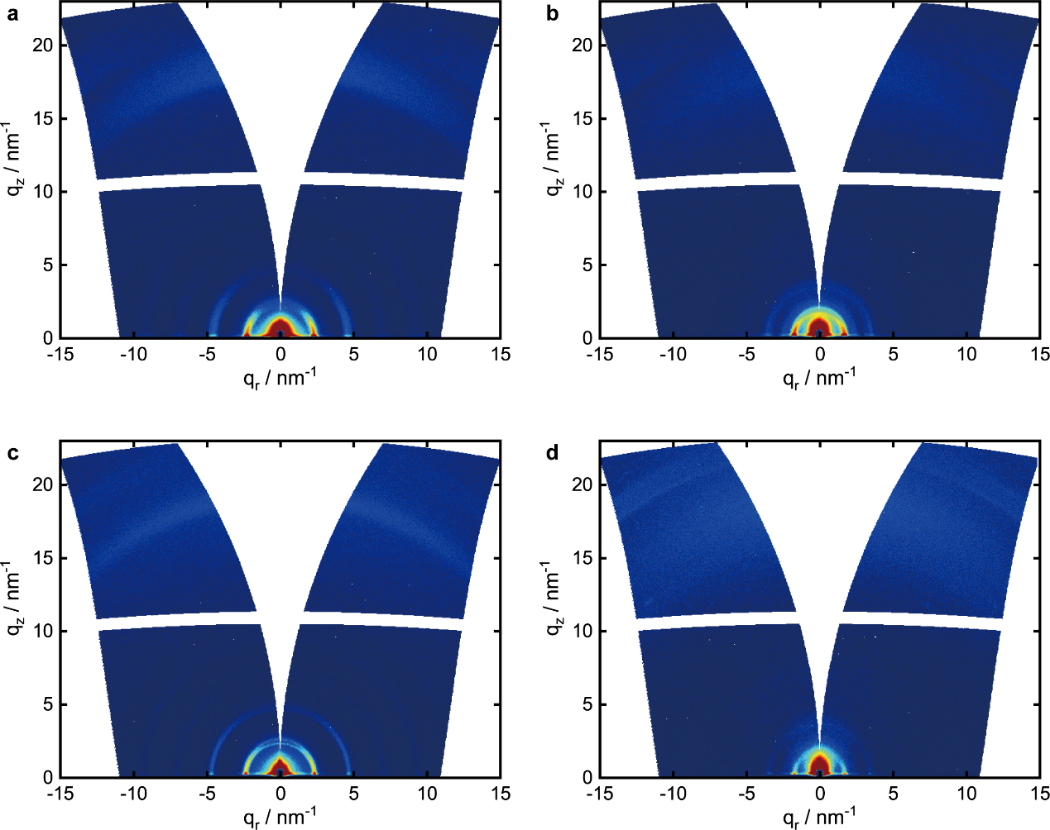


**Figure S23**. GIWAXS 2D patterns of **TT** and **BDT(BT)_2_** thin films on ITO before (**a**, **b**) and after (**c**, **d**) chronoamperometric measurements after chopped light (1.5 AM) illumination over a total duration of one hour. Both films were washed with distilled water after the chronoamperometric measurement.

3.17 Electrochemical Impedance Spectroscopy (EIS) Analyses of COF Films


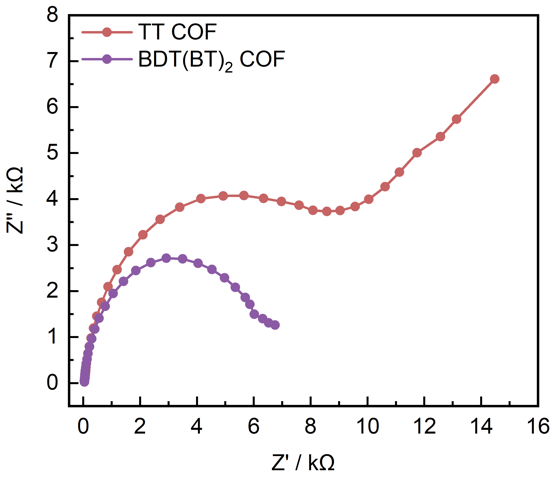


**Figure S24**. Impedance analyses for **TT** (red trace) and **BDT(BT)_2_ COF** (purple trace) films.

3.18 Surface Wettability of COF Films Assessed via Water Contact Angle Measurements


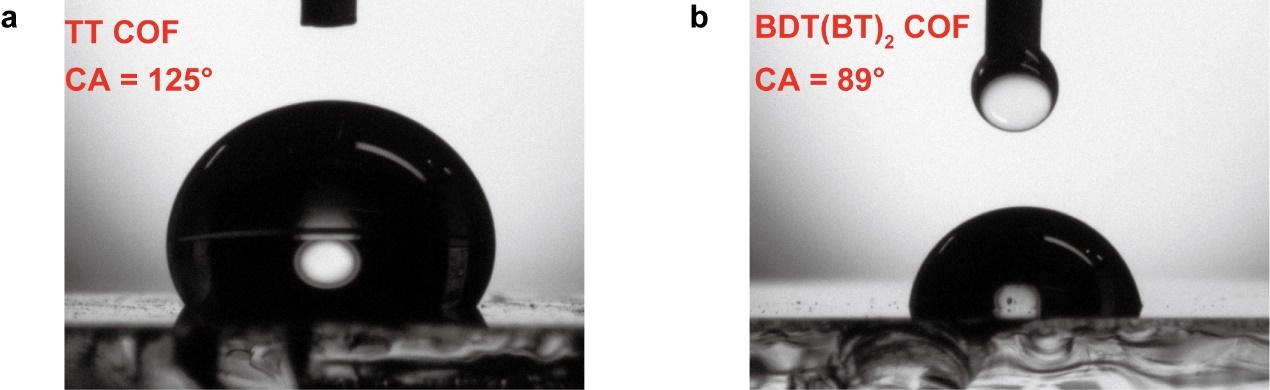


**Figure S25**. Water contact angles measured at the solid/air interface of (**a**) **TT** and (**b**) **BDT(BT)_2_ COF** films.

3.19 Structural analysis

**Unit cell parameters (*C2/m*) and atomic coordinates for TT COF**

**a = 46.0, b = 37.9, c = 3.9 Å**

**α = 90.0°, β = 64.1°, γ = 90.0°**

| **Atom** | **a/x** | **b/y** | **c/z** |
| --- | --- | --- | --- |
| \| C1 \| \| --- \| \| C2 \| \| S3 \| \| C4 \| \| C5 \| \| C6 \| \| C7 \| \| C8 \| \| C9 \| \| C10 \| \| C11 \| \| N12 \| \| C13 \| \| C14 \| \| C15 \| \| C16 \| \| S17 \| \| H18 \| \| H19 \| \| H20 \| \| H21 \| \| H22 \| \| H23 \| \| H24 \| \| C25 \| | \| 0.46173 \| \| --- \| \| 0.48127 \| \| 0.58375 \| \| 0.57453 \| \| 0.53923 \| \| 0.59983 \| \| 0.58057 \| \| 0.60259 \| \| 0.64425 \| \| 0.66398 \| \| 0.64205 \| \| 0.66359 \| \| 0.7066 \| \| 0.72103 \| \| 0.76717 \| \| 0.77056 \| \| 0.68247 \| \| 0.52838 \| \| 0.54783 \| \| 0.58721 \| \| 0.69513 \| \| 0.65759 \| \| 0.73311 \| \| 0.79641 \| \| 0.48009 \| | \| 1.53442 \| \| --- \| \| 1.56693 \| \| 1.54187 \| \| 1.58808 \| \| 1.59737 \| \| 1.61455 \| \| 1.65003 \| \| 1.67354 \| \| 1.6625 \| \| 1.62724 \| \| 1.60364 \| \| 1.6872 \| \| 1.6847 \| \| 1.71196 \| \| 1.71076 \| \| 1.73936 \| \| 1.74805 \| \| 1.62504 \| \| 1.65958 \| \| 1.70065 \| \| 1.61722 \| \| 1.57644 \| \| 1.66325 \| \| 1.6907 \| \| 0.5 \| | \| 0.58434 \| \| --- \| \| 0.53853 \| \| 0.32472 \| \| 0.3683 \| \| 0.43312 \| \| 0.36903 \| \| 0.5536 \| \| 0.58388 \| \| 0.4293 \| \| 0.24102 \| \| 0.21052 \| \| 0.49122 \| \| 0.29747 \| \| 0.3995 \| \| 0.19125 \| \| 0.34638 \| \| 0.78401 \| \| 0.46545 \| \| 0.68617 \| \| 0.72945 \| \| 0.13113 \| \| 0.07165 \| \| 0.05039 \| \| -0.06057 \| \| 0.54539 \| |
|  |  |  |  |
|  |  |  |  |

**Unit cell parameters (*C2/m*) and atomic coordinates for BDT(BT)_2_ COF**

**a = 60.8, b = 53.0, c = 3.9 Å**

**α = 90.0°, β = 63.6°, γ = 90.0°**

| **Atom** | **a/x** | **b/y** | **c/z** |
| --- | --- | --- | --- |
| \| C1 \| \| --- \| \| C2 \| \| S3 \| \| C4 \| \| C5 \| \| C6 \| \| C7 \| \| C8 \| \| C9 \| \| C10 \| \| C11 \| \| N12 \| \| C13 \| \| C14 \| \| C15 \| \| C16 \| \| C17 \| \| C18 \| \| C19 \| \| N20 \| \| S21 \| \| N22 \| \| C23 \| \| S24 \| \| C25 \| \| C26 \| \| C27 \| \| C28 \| \| O29 \| \| C30 \| \| C31 \| \| H32 \| \| H33 \| \| H34 \| \| H35 \| \| H36 \| \| H37 \| \| H38 \| \| H39 \| \| H40 \| \| H41 \| \| H42 \| \| H43 \| \| H44 \| \| H45 \| \| C46 \| | \| 0.47463 \| \| --- \| \| 0.4876 \| \| 0.55607 \| \| 0.55031 \| \| 0.52627 \| \| 0.56921 \| \| 0.56217 \| \| 0.58004 \| \| 0.6049 \| \| 0.61222 \| \| 0.5945 \| \| 0.62161 \| \| 0.64508 \| \| 0.65976 \| \| 0.68644 \| \| 0.70121 \| \| 0.68962 \| \| 0.66266 \| \| 0.64829 \| \| 0.62294 \| \| 0.6164 \| \| 0.64809 \| \| 0.70561 \| \| 0.6931 \| \| 0.72401 \| \| 0.74241 \| \| 0.73213 \| \| 0.76849 \| \| 0.71481 \| \| 0.69452 \| \| 0.66739 \| \| 0.51905 \| \| 0.54291 \| \| 0.57453 \| \| 0.63126 \| \| 0.60069 \| \| 0.6547 \| \| 0.69608 \| \| 0.7217 \| \| 0.74403 \| \| 0.69374 \| \| 0.69906 \| \| 0.66263 \| \| 0.65177 \| \| 0.66684 \| \| 0.48681 \| | \| 0.52421 \| \| --- \| \| 0.54713 \| \| 0.52934 \| \| 0.56179 \| \| 0.5685 \| \| 0.58005 \| \| 0.60577 \| \| 0.62222 \| \| 0.61337 \| \| 0.58786 \| \| 0.57141 \| \| 0.63083 \| \| 0.6264 \| \| 0.64682 \| \| 0.64276 \| \| 0.66207 \| \| 0.68602 \| \| 0.68947 \| \| 0.67051 \| \| 0.67717 \| \| 0.70732 \| \| 0.71061 \| \| 0.70655 \| \| 0.73622 \| \| 0.74607 \| \| 0.72626 \| \| 0.70402 \| \| 0.72901 \| \| 0.79294 \| \| 0.79745 \| \| 0.79929 \| \| 0.58797 \| \| 0.61329 \| \| 0.64196 \| \| 0.58025 \| \| 0.55178 \| \| 0.60802 \| \| 0.62478 \| \| 0.65786 \| \| 0.68698 \| \| 0.78239 \| \| 0.81593 \| \| 0.78094 \| \| 0.80234 \| \| 0.81596 \| \| 0.5 \| | \| 0.58505 \| \| --- \| \| 0.53385 \| \| 0.3118 \| \| 0.37846 \| \| 0.45096 \| \| 0.37771 \| \| 0.52456 \| \| 0.53385 \| \| 0.40928 \| \| 0.25688 \| \| 0.24516 \| \| 0.45301 \| \| 0.36241 \| \| 0.42156 \| \| 0.25885 \| \| 0.29597 \| \| 0.50609 \| \| 0.66858 \| \| 0.63304 \| \| 0.81722 \| \| 1.03414 \| \| 0.87584 \| \| 0.5435 \| \| 0.78467 \| \| 0.62713 \| \| 0.43495 \| \| 0.38396 \| \| 0.31513 \| \| 0.87152 \| \| 0.79659 \| \| 1.24406 \| \| 0.49476 \| \| 0.63608 \| \| 0.64616 \| \| 0.15394 \| \| 0.13241 \| \| 0.23158 \| \| 0.09417 \| \| 0.15802 \| \| 0.22936 \| \| 0.58966 \| \| 0.60802 \| \| 1.43687 \| \| 1.18023 \| \| 1.44346 \| \| 0.54771 \| |

4. References

[1] M. Righetto, Y. Wang, K. A. Elmestekawy, C. Q. Xia, M. B. Johnston, G. Konstantatos, L. M. Herz, *Adv. Mater.*﻿ **2023**, *35*, 2305009.

[2] F. M. Wagner, S. Melnikas, J. Cramer, D. A. Damry, C. Q. Xia, K. Peng, G. Jakob, M. Kläui, S. Kičas, M. B. Johnston, *J. Infrared Millim. Terahertz Waves.* **2023**, *44*, 52-65.

[3] T. Sick, A. G. Hufnagel, J. Kampmann, I. Kondofersky, M. Calik, J. M. Rotter, A. Evans, M. Döblinger, S. Herbert, K. Peters, D. Böhm, P. Knochel, D. D. Medina, D. Fattakhova-Rohlfing, T. Bein, *J. Am. Chem. Soc.* **2017**, *140*, 2085-2092.

[4] A. Yamamoto, Y. Matsui, T. Asada, M. Kumeda, K. Takagi, Y. Suenaga, K. Nagae, E. Ohta, H. Sato, S. Koseki, H. Naito, H. Ikeda, *J. Org. Chem.* **2016**, *81*, 3168-3176.

[5] C. Shen, Y. Wu, H. Zhang, E. Li, W. Zhang, X. Xu, W. Wu, H. Tian, W. H. Zhu, *Angew. Chem. Int. Ed.* **2019**, *58*, 3784-3789.

[6] T. Sick, J. M. Rotter, S. Reuter, S. Kandambeth, N. N. Bach, M. Döblinger, J. Merz, T. Clark, T. B. Marder, T. Bein, D. D. Medina, *J. Am. Chem. Soc.* **2019**, *141*, 12570-12581.

[7] T. Xue, R. Guntermann, A. Biewald, D. Blätte, D. D. Medina, A. Hartschuh, T. Bein, *ACS Appl. Mater. Interfaces* **2024**, *16*, 48085-48093.

[8] C. Wehrenfennig, G. E. Eperon, M. B. Johnston, H. J. Snaith, L. M. Herz, *Adv. Mater. (Deerfield Beach, Fla.)* **2013**, *26*, 1584.

[9] H. J. Joyce, J. L. Boland, C. L. Davies, S. A. Baig, M. B. Johnston, *Semicond. Sci. Technol.* **2016**, *31*, 103003.

[10] L. Spies, A. Biewald, L. Fuchs, K. Merkel, M. Righetto, Z. Xu, R. Guntermann, R. Hooijer, L. M. Herz, F. Ortmann, J. Schneider, T. Bein, A. Hartschuh, *J. Am. Chem. Soc.* **2025**, *147*, 1758-1766.

[11] H. Hempel, T. J. Savenjie, M. Stolterfoht, J. Neu, M. Failla, V. C. Paingad, P. Kužel, E. J. Heilweil, J. A. Spies, M. Schleuning, J. Zhao, D. Friedrich, K. Schwarzburg, L. D. A. Siebbeles, P. Dörflinger, V. Dyakonov, R. Katoh, M. J. Hong, J. G. Labram, M. Monti, E. Butler-Caddle, J. Lloyd-Hughes, M. M. Taheri, J. B. Baxter, T. J. Magnanelli, S. Luo, J. M. Cardon, S. Ardo, T. Unold, *Adv. Energy Mater.* **2022**, 12, 2102776.

[12] A. M. Ulatowski, L. M. Herz, M. B. Johnston, *J. Infrared Millim. Terahertz Waves.* **2020**, *41*, 1431-1449.

[13] R. Ulbricht, E. Hendry, J. Shan, T. F. Heinz, M. Bonn, *Rev. Mod. Phys*. **2011**, *83*, 543-586.

[14] R. L. Milot, G. E. Eperon, H. J. Snaith, M. B. Johnston, L. M. Herz, *Adv. Funct. Mater.* **2015**, *25*, 6218-6227.
